# Dual FLT3/MAPK14 Proteolysis-Targeting Chimera (PROTAC) Induces Potent Acute Myeloid Leukemia Cell Death

**DOI:** 10.3390/ph19050756

**Published:** 2026-05-12

**Authors:** Mohamed Abdelsalam, Melisa Halilovic, Ramy Ashry, Husam Nassar, Frank Erdmann, Matthias Schmidt, Oliver H. Krämer, Wolfgang Sippl

**Affiliations:** 1 Institute of Pharmacy, Martin-Luther University of Halle-Wittenberg, 06120 Halle, Germany; mohamed.abdelsalam@pharmazie.uni-halle.de (M.A.); husam.nassar@student.uni-halle.de (H.N.); frank.erdmann@pharmazie.uni-halle.de (F.E.); matthias.schmidt@pharmazie.uni-halle.de (M.S.); 2 Department of Pharmaceutical Chemistry, Faculty of Pharmacy, Alexandria University, Alexandria 21521, Egypt; 3 Institute of Toxicology, University Medical Center, Johannes Gutenberg-University Mainz, 55131 Mainz, Germany; halilovm@uni-mainz.de (M.H.); relashry@uni-mainz.de (R.A.)

**Keywords:** acute myeloid leukemia, FLT3-ITD mutation, MAPK14 (p38α), PROTACs, synergistic effect

## Abstract

**Background/Objectives**: Acute myeloid leukemia (AML) is a hematological malignancy frequently driven by mutations in the FLT3 gene, particularly internal tandem duplications (FLT3-ITD), which contribute to aberrant cell proliferation and resistance to tyrosine kinase inhibitors (FLT3i). The limitations of current FLT3i therapies, including drug resistance, off-target effects, and poor selectivity, necessitate the development of novel therapeutic strategies. Proteolysis-targeting chimeras (PROTACs) represent a promising approach to achieving degradation of oncogenic proteins. **Methods**: We developed FLT3-targeting PROTACs based on the previously described compound **MA49**, with a focus on linker modifications to improve degradation efficiency and pharmacokinetic properties. **Results**: Among these, compounds **MA190** and **MA191**, containing rigid cyclohexyl-piperidine/piperazine linkers, demonstrate superior degradation of FLT3-ITD in MV4-11 AML cells at nanomolar concentrations, achieving >95% reduction in FLT3-ITD levels, outperforming **MA49**. In addition to improved kinase selectivity, good solubility, and plasma stability, **MA190** and **MA191** also exhibit excellent metabolic stability, whereas the predecessor PROTAC **MA49** was unstable in microsomal assays. In cellular assays, **MA190** and **MA191** induce potent apoptosis in FLT3-ITD^+^ AML cells but have minimal effects on cells with wild-type FLT3. Proteomics reveal that **MA191** also degrades MAPK14 (p38α), a kinase upregulated in leukemia, in addition to FLT3. **Conclusions**: Dual targeting of FLT3-ITD and MAPK14 enhances proapoptotic signaling without any cytotoxic effect on normal human HEK293 cells. The co-inhibition using **MA191** or a combination of doramapimod (a MAPK14 inhibitor) with a non-degrading FLT3 inhibitor result in greater caspase-3 activation than either treatment alone. This synergistic effect can be a therapeutic advantage, as several oncogenic drivers are switched off simultaneously by **MA191**.

## 1. Introduction

Acute myeloid leukemia (AML) is one of the most aggressive hematologic malignancies worldwide and continues to pose a challenge in clinical treatment [1]. The *FLT3* gene (FMS-like tyrosine kinase 3) encodes a class III receptor tyrosine kinase that is expressed in hematopoietic cells and plays a critical role in hematopoiesis [2]. FLT3 mutations are among the most common genetic alterations and occur in approximately 30% of AML patients. The majority of these mutations involve internal tandem duplications (FLT3-ITD) in the juxtamembrane domain of the *FLT3* gene [2]. Other mutations include point mutations in the tyrosine kinase domain (TKD) at the C-terminus. FLT3-ITD and FLT3-TKD mutations lead to constitutive activation of the FLT3 kinase and its downstream signaling pathways, resulting in the proliferation of malignant cells [3]. FLT3 mutations are also associated with resistance to current FLT-3 inhibitors, which often leads to a poor prognosis, and a lower overall survival rate [4,5]. Consequently, mutated FLT3 (especially FLT3-ITD) is considered a clinically validated target for AML treatment. Although several FLT3 inhibitors (FLT3i) have been developed and have shown positive clinical outcomes against AML, they face multiple limitations such as low selectivity, development of resistance, and dose-limiting toxicity [6,7,8]. The FLT3 kinase can adopt either an active or inactive conformation depending on the orientation of the amino acid sequence aspartate–phenylalanine–glycine (DFG motif) in the activation loop. Accordingly, FLT3i are classified into type I and type II inhibitors based on their binding to the active or inactive form of the kinase domain. Type I FLT3i, such as midostaurin, gilteritinib, and sunitinib, preferentially inhibit the active conformation (DFG-in position) [9]. In contrast, type II FLT3i, such as sorafenib and quizartinib, can only bind to the inactive conformation (DFG-out position). This binding also involves hydrophobic interactions with the back pocket of the ATP-binding region, resulting in higher potency and selectivity compared to type I inhibitors [9]. Substitutions of aspartic acid D835 in FLT3 are the most common secondary TKD point mutations and lead to weakened binding of inhibitors [10]. As a result, patients frequently develop rapid resistance after treatment with FLT3i, which ultimately leads to a short response duration to these drugs [11,12,13]. Another challenge in using FLT3i is that high doses are usually required for maximum and sustained inhibition of mutant FLT3, which is potentially associated with numerous off-target effects [14,15]. Although numerous efforts have been made to develop new inhibitors of mutated FLT3 [6], there remains a need to develop new classes of agents that specifically target mutated and hyperactive FLT3 (i.e., FLT3-ITD) and utilize alternative pharmacological mechanisms [16,17,18]. These strategies may overcome the current limitations of conventional FLT3i. 

PROTACs (proteolysis targeting chimeras), which trigger the targeted degradation of proteins (TPD), have aroused great interest in recent years. This innovative therapeutic strategy can target disease-associated proteins that have been difficult to inhibit. A classical PROTAC molecule consists of a ligand for the protein of interest (POI), a ligand for an E3 ubiquitin ligase, and a suitable linker connecting both ligands. PROTACs promote proximity between the POI and the E3 ligase, facilitating ubiquitin transfer to the surface of the POI, thereby marking it for proteasomal degradation [19]. In contrast to conventional inhibition, protein degradation via PROTACs is an event-driven process, and degraders act in a sub-stoichiometric and catalytic manner (i.e., the PROTAC molecule can be recycled, resulting in target protein degradation at lower doses compared to classical inhibitors, thus potentially reducing side effects) [19,20]. Moreover, TPD may overcome resistance mechanisms associated with traditional inhibitors, such as mutation suppression or overexpression of the POI [21]. 

**Figure 1:**
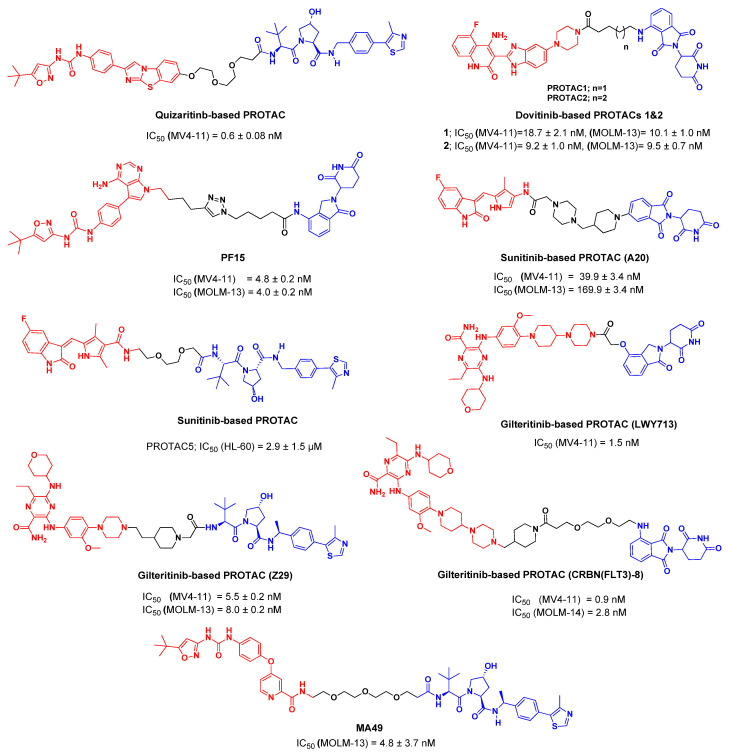
Reported FLT3 PROTACs derived from type-I and type-II FLT3 inhibitors.

Interestingly, degradation of FLT3-ITD through PROTAC technology has proven to be a promising approach to enhance the efficacy of FLT3-ITD inhibitors by maximizing and prolonging the removal of this mutant protein. Several FLT3 PROTACs have been developed based on the structures of different known FLT3i (Figure 1). In 2018, the Crews group reported the first FLT3 PROTAC based on quizartinib, which strongly induced FLT3 degradation in leukemic cell lines [22]. This PROTAC is like quizartinib less active against FLT3-ITD with TKD mutations. In 2021, **PF15** was reported by Chen et al., which showed degradation of FLT3-ITD in transformed BaF3 cells [23]. Furthermore, other FLT3 PROTACs have been recently reported based on dovitinib [24], sunitinib [25,26] and gilteritinib [27,28,29] through tethering these FLT3 warheads to the Von Hippel-Lindau (VHL) or different cereblon E3 ligase ligands using different linkers. Some of them also degrade the structurally similar kinase c-Kit, which should be avoided. Simultaneous inhibition of FLT3 and c-KIT suppresses normal hematopoiesis. Recently, we reported the VHL-based PROTAC (**MA49**), which is derived from the rationally designed type II FLT3i **MA68** (a combination of the pharmacophoric features of sorafenib and quizartinib) [30]. Sorafenib was reported to potently inhibit nine protein kinases, including FLT3 and MAPK14 and thus represents a multi-kinase inhibitor [31]. Nevertheless, we showed that the sorafenib analog–based PROTAC **MA49** induces VHL-dependent degradation of FLT3-ITD in MV4-11 and MOLM-13 cells at nanomolar concentrations, while sparing wild-type FLT3 and the related kinase c-KIT [30]. 

Several studies reported that the linker length and flexibility could significantly affect the bio-degradative efficacy of PROTACs, as both structural parameters could contribute to the formation of stable ternary complex [32,33]. Moreover, the linker composition has an impact on the physicochemical and pharmacokinetic properties of PROTACs [34]. Here we disclose an optimized series of FLT3 PROTACs based on our previously reported FLT3 PROTAC (**MA49**) through modification of the linker part. We have recently generated and validated an in silico model of the ternary complex MA49-FLT3-VHL [35] that showed high stability in molecular dynamics simulations. Using the model of **MA49**, we found that the flexible PEG linker can also be replaced by shorter linkers without changing the binding of the resulting PROTACs or the two warheads (see modeling section for details).

## 2. Results and Discussion

### 2.1. Rational Optimization of the FLT3-ITD PROTAC (MA49)

**Figure 2:**
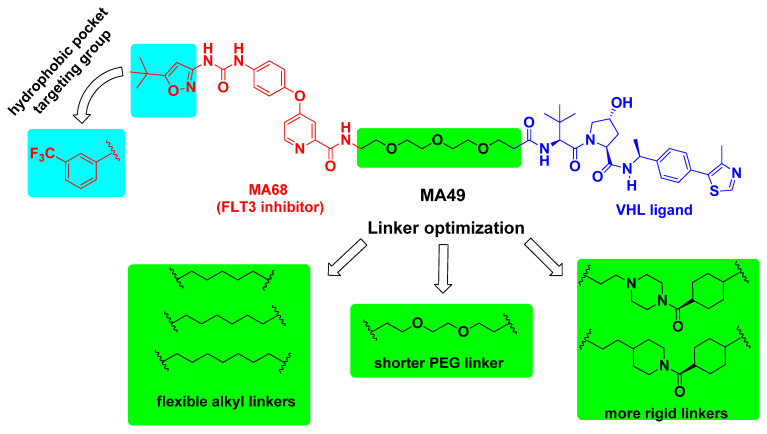
Design of a series of FLT3-ITD/VHL-based PROTACs by modification of PROTAC MA49.

Influenced by the description of effective VHL-targeting kinase PROTACs in which a relatively short distance between the VHL ligand and kinase inhibitor was used, we reduced the linker length of **MA49**. This was achieved on the one hand by using a shorter PEG-2 linker or correspondingly shorter alkyl chains (Figure 2). On the other hand, this included the use of stiffer cycloalkane-based linkers with similar distances between the two ligands (Figure 2). The use of more rigid linkers with fewer degrees of freedom can lead to greater effectiveness by reducing entropy effects and improving the drug-like properties of PROTACs [16,29]. The linker design was supported by modeling of the ternary complexes of VHL-FLT3, as described in detail in Section 2.11. In addition, the *tert*-butyl isoxazole moiety was replaced with a more hydrophobic 3-trifluoromethylphenyl ring to enhance hydrophobic interactions within the DFG-out back pocket of the kinase, with the aim of enhancing binding affinity and stabilizing the inactive kinase conformation (Figure 2). 

### 2.2. Chemistry

**Scheme 1:**
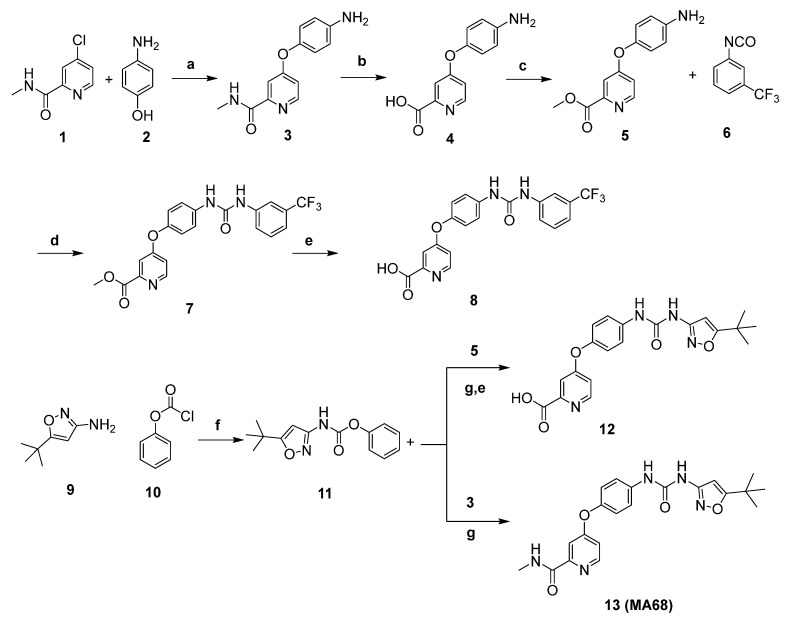
Synthesis of carboxylic acid derivatives **8** and **12** and the FLT3 inhibitor **13** (**MA68**). Reagents and conditions: (**a**) t-BuOK, K_2_CO_3_, DMF 80 °C, 3 h; (**b**) aq. KOH, reflux, 5 h; (**c**) SOCl_2_, MeOH, reflux, 8 h; (**d**) DCM, RT, 3 h; (**e**) LiOH·H_2_O, THF, H_2_O, RT, 3 h; (**f**) pyridine, THF, RT, 3 h; and (**g**) DMAP, DMF, 50 °C, overnight.

Generally, the synthesis of the planned PROTACs involved three main steps. First, the FLT3 inhibitor warheads were synthesized and functionalized with a free carboxylic acid group. The second step involved the amide coupling reaction between the inhibitor part and the appropriate linkers. Finally, the coupling of the inhibitor-linker intermediates and the VHL ligand afforded the desired degraders. The synthetic procedures for the key carboxylic acid intermediates **8** and **12** are outlined in Scheme 1. First, 4-chloro-*N*-methylpicolinamide (**1**) was reacted with 4-aminophenol (**2**) under basic conditions, followed by alkaline aqueous hydrolysis to obtain the carboxylic acid intermediate **4**. Esterification of intermediate **4** using thionyl chloride in methanol afforded the corresponding methyl ester **5**, which was treated with 3-(trifluormethyl)phenylisocyanate (**6**) to yield the corresponding urea derivative **7**. The methyl ester group of intermediate **7** was hydrolyzed using lithium hydroxide to afford the key carboxylic acid intermediate **8**. The carboxylic acid intermediate **12** was synthesized by activation of the 3-amino-5-*tert*-butylisoxazole (**9**) using phenyl chloroformate (**10**) to get the corresponding carbamate derivative **11**. Heating the carbamate derivative **11** with intermediate **5** in the presence of 4-dimethylaminopyridine (DMAP), followed by ester hydrolysis afforded the carboxylic acid intermediate **12** [30]. Similarly, reacting intermediates **3** and **11** under the same reaction condition afforded the reference inhibitor **13** (**MA68**) [30]. 

**Scheme 2:**
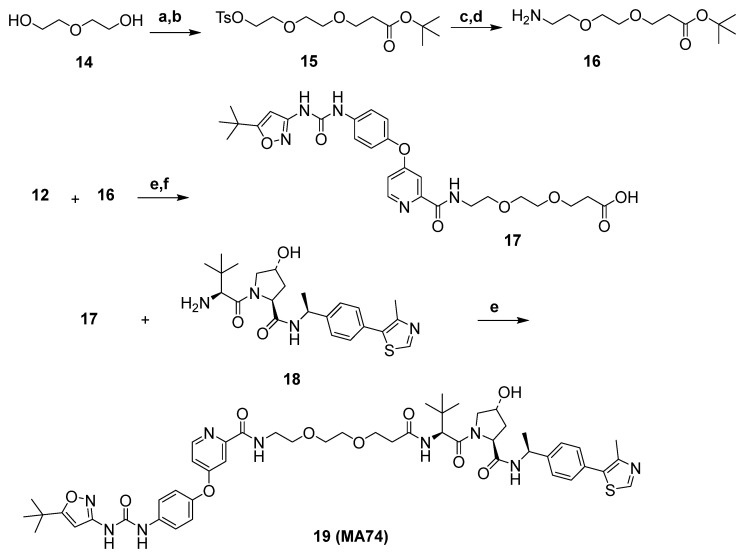
Synthesis of PROTAC **19** (**MA74**). Reagents and conditions: (**a**) Sodium metal, tert-butyl acrylate, THF, RT, overnight; (**b**) tosyl chloride, Et_3_N, DCM, 0 °C, overnight; (**c**) sodium azide, DMF, RT, overnight; (**d**) PPh_3_, H_2_O, THF, RT, overnight; (**e**) HATU, DIPEA, DMF, RT, 4 h; and (**f**) TFA, DCM, 0 °C to RT, 3 h.

The synthesis of PROTAC **19** (**MA74**), which consists of PEG-2 units linker is illustrated in Scheme 2. First, the linker part was prepared as previously reported [36], starting from the reaction of diethylene glycol and *tert*-butyl acrylate in the presence of sodium metal, followed by tosylation reaction to obtain intermediate **15**. The tosylate intermediate was subjected to a nucleophilic substitution reaction using sodium azide in DMF to afford the azido ester, followed by reduction of azide to the corresponding amine using triphenylphosphine and water to obtain the amino ester containing PEG linker **16**. The obtained linker was coupled with carboxylic acid **12** using HATU as a coupling agent, followed by *tert*-butyl ester hydrolysis using trifluoroacetic acid to get intermediate **17**. The latter was reacted with the VHL amino building block (S,R,S)-AHPC-Me **18** which was prepared following the previously reported procedure [37] to obtain PROTAC **19** (**MA74**). 

**Scheme 3:**
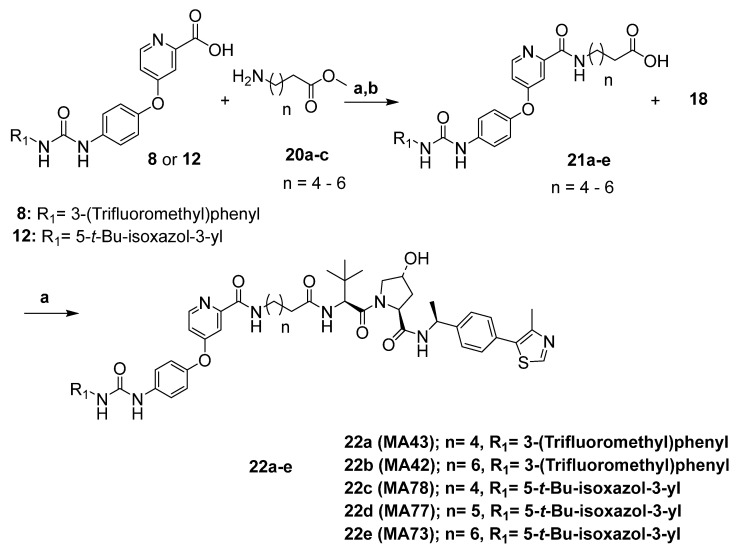
Synthesis of the VHL-based PROTACs **22a**–**e**. Reagents and conditions: (**a**) HATU, DIPEA, DMF, RT, 5 h; and (**b**) LiOH·H_2_O, THF, H_2_O, RT, 5 h.

Then, the series of VHL-based PROTACs containing alkyl chain linkers, were synthesized as shown in Scheme 3. The appropriate carboxylic acid derivative **8** or **12** was reacted through the amide coupling with the appropriate linkers **20a**–**c**, followed by hydrolysis of methyl ester to afford intermediates **21a**–**e**. Finally, the amide coupling reaction of intermediates **21a**–**e** and the VHL amine **18** afforded the final compounds **22a**–**e** (**MA43**, **MA42**, **MA78**, **MA77**, **MA73**).

**Scheme 4:**
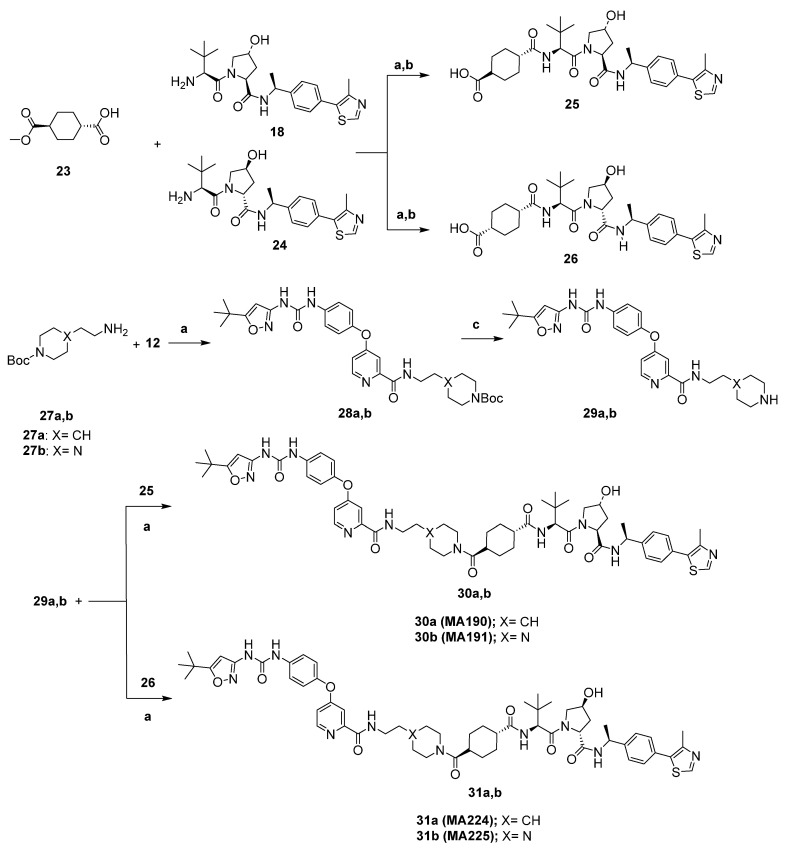
Synthesis of the VHL-based PROTACs **30a**,**b** (**MA190** and **MA191**) and negative controls **31a**,**b** (**MA224** and **MA225**). Reagents and conditions: (**a**) HATU, DIPEA, DMF, RT, 5 h; (**b**) LiOH·H_2_O, THF, H_2_O, RT, 4 h; and (**c**) TFA, DCM, 0 °C to RT, 30–60 min.

The third group of VHL-based PROTACs, containing more rigid linkers, **30a**,**b** (**MA190** and **MA191**) and their corresponding VHL negative controls **31a**,**b** (**MA224** and **MA225**), were prepared as outlined in Scheme 4. First, trans-1,4-cyclohexanedicarboxylic acid monomethyl ester (**23**) was coupled with the VHL amine **18** or its inactive diastereomeric analog **24** with inverted stereocenters on the hydroxyproline moiety, which causes loss of binding affinity to VHL [38,39,40], followed by methyl ester hydrolysis to yield intermediates **25** and **26**, respectively. The second part of the synthesis involved the amide coupling between the *N*-Boc-protected piperidine/piperidine analogs **27a**,**b** and intermediate **12**, followed by Boc-deprotection to get intermediates **29a**,**b**. Finally, the amide coupling of the carboxylic acid intermediate **25** or **26** and the appropriate amine derivative **27a**,**b** afforded the final compounds **30a**,**b** (**MA190**, **MA191**) and **31a**,**b** (**MA224**, **MA225**), respectively. The structures of the synthesized VHL-PROTACs are outlined in Table 1.

### 2.3. Kinase Binding Using KINOMEscan Assay

Binding to FLT3-ITD was determined using the KINOMEscan^TM^ assay (Eurofins DiscoverX Corporation, San Diego, CA, USA) [41] (Table 1) and showed that the tested PROTACs exhibit strong binding at 1 µM concentration. PROTACs **MA190**/**MA191** and their negative control analog **MA225** showed in vitro inhibition of FLT3-ITD in the same range as observed before for **MA49** [27].

### 2.4. Kinase Selectivity Profiling of MA68 and MA191

**Figure 3:**
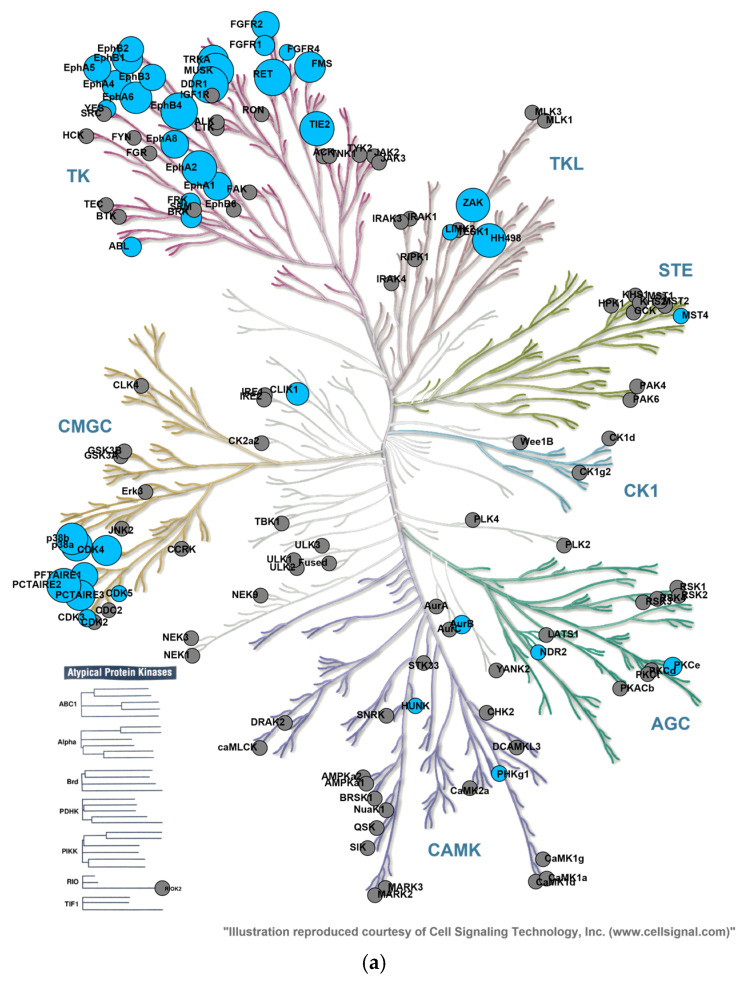

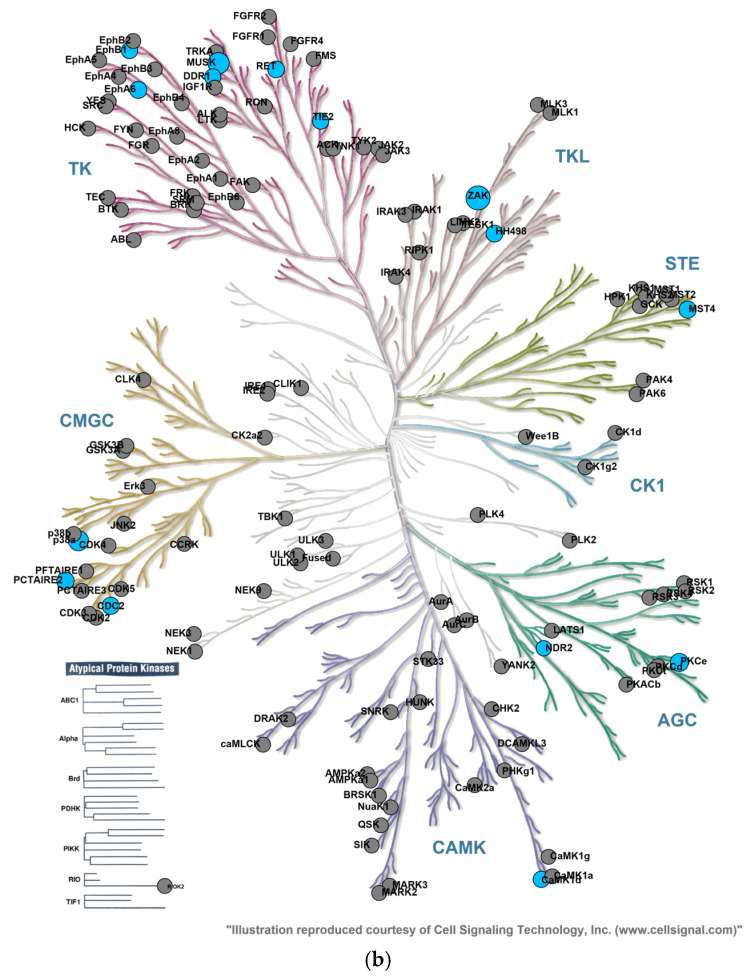
Kinase selectivity profiling of (**a**) MA68 and (**b**) MA191. (Exact binding values are listed in Table S1, Supplementary Information). Depicted is the tracer bound [%] where a smaller value equals more competition by the test compounds which equals more potency and a bigger circle, respectively. The blue circle size corresponds to the inversed respective Tracer bound [%] value measured which corresponds to a bigger circle meaning higher potency. A gray circle is depicted if the tracer bound value is above 91% indicating weak test compound binding.

To get an impression about the binding strength of the PROTAC **MA191** and the reference inhibitor **MA68** to other kinases besides FLT3-ITD the binding on a kinase panel of further 128 representative protein kinases at 1 μM concentration was tested using the NanoBRET^TM^ cellular target engagement assay (CELLinib128 assay, CELLinib GmbH, Frankfurt, Germany) [42,43]. Interestingly, at this concentration, **MA191** showed very weak binding to most of the kinases used in the test set. In none of the 128 kinases **MA191** was able to displace the tracer (inhibitor) used by more than 50%, Table S1, Supplementary Information). The kinase selectivity of the PROTAC **MA191** was significantly improved compared to its parent inhibitor **MA68**, which showed >50% displacement of the kinase inhibitor (tracer) used for 25 of the tested kinases in the cellular NanoBret assay (e.g., MAPK, CDK and Eph tyrosine kinase families) (Figure 3, Table S1, Supplementary Information). Furthermore, **MA191** showed minimal interaction with most kinases, with no detectable binding (<10%) observed in 93.7% of the kinases. 

### 2.5. Cellular Studies

#### 2.5.1. Degradation of FLT3-ITD in AML Cells 

We treated MV4-11 cells with the synthesized degraders of FLT3. These human AML cells carry two copies of FLT3-ITD. The FLT3 band that appears at 130 kDa in Western blot analyses corresponds to the oncogenic, hypoglycosylated ER-bound FLT3-ITD. The band at 160 kDa is the plasma membrane-located FLT3-ITD, resembling FLT3. In our previous study, we identified the VHL-addressing compound **MA49** [30] as the most anti-leukemic FLT3 PROTAC. **MA49** depletes preferentially the oncogenic, ER-bound FLT3-ITD and is more effective than its cognate inhibitor that cannot recruit VHL. This is associated with an accumulation of the apoptosis-promoting cleaved form of caspase-3 [30].

**Figure 4:**
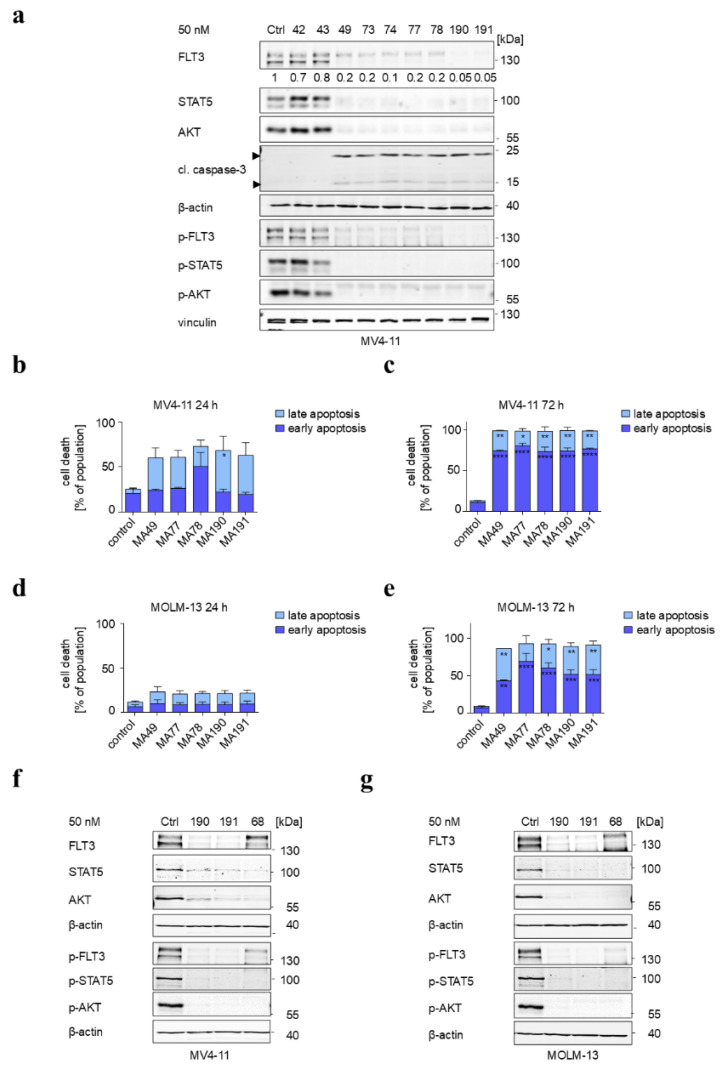
Biological and biochemical characterization of the FLT3 PROTACs. (**a**) MV4-11 cells were incubated for 24 h with 50 nM of the indicated compounds. Immunoblotting was performed for the indicated proteins and their phosphorylation (p-) or cleavage (cl.; indicated by arrows); β-actin was used to ensure equal sample loading. (**b**) MV4-11 cells were treated with the indicated agents and analyzed for early and late apoptosis by flow cytometry. (**c**) Same as in (**b**) but treatment for 72 h. (**d**) MOLM-13 cells were treated with the indicated agents and analyzed for early and late apoptosis by flow cytometry. (**e**) Same as in (**b**) but treatment for 72 h. (**f**,**g**) MV4-11 and MOLM-13 cells were incubated for 24 h with 50 nM MA190 or MA191. Immunoblots show the expression of the indicated proteins and their phosphorylation (p-); β-actin was used to ensure equal sample loading. All immunoblots show data from at least two independent repetitions. All immunoblots show data from at least two independent repetitions and flow cytometry analyses were conducted thrice independently; * *p* < 0.1, ** *p* < 0.01, *** *p* < 0.001, **** *p* < 0.0001.

The specificity of **MA49** for the hyperactive FLT3-ITD encouraged us to develop additional, optimized degraders of this oncoprotein. We applied **MA49** and newly synthesized, putative FLT3 degraders to MV4-11 cells. **MA42** and **MA43** containing the trifluorophenyl instead of the 5-*tert*-butylisoxazole group in the FLT3 inhibitor part evoked no significant degradation (Figure 4a). Compared to **MA49**, the new PROTACs with the *tert*-butylisoxazole group and a shorter linker between VHL and FLT3 ligand showed stronger degradation at 50 nM (**MA73**, **MA74**, **MA77**, and **MA78**). Of these compounds, PROTACs **MA190** and **MA191**, which contain more rigid linkers than the other degraders, reduced FLT3-ITD (130 kDa) to 5% of the levels in untreated cells at 50 nM concentration. These agents were also superior to our other PROTACs including the previously developed **MA49** which eliminated 80% of FLT3-ITD under like conditions (Figure 4a).

The elimination of FLT3-ITD was associated with a reduced expression of its target protein STAT5 and AKT, and the activation of caspase-3 by limited proteolysis (Figure 4a). These findings correspond to our previous data showing a loss of STAT5 and AKT by caspase-dependent and transcriptional repression mechanisms, [44,45] respectively. When we analyzed the phosphorylation-dependent activation of STAT5 and AKT by FLT3-ITD and its auto-phosphorylation, we found that the PROTAC-induced elimination of FLT3-ITD stalled the constitutive phosphorylation of these cancer-associated proteins in MV4-11 cells.

#### 2.5.2. Apoptosis Induction in AML Cells 

To further assess the biological relevance of these findings, we treated MV4-11 cells with the new PROTACs, included **MA49** as control, and analyzed them for early (annexin-V positive) and late apoptosis (annexin-V and propidium iodine positive) by flow cytometry. Cells in late apoptosis are more severely damaged than cells in early apoptosis. After a 24 h incubation period with the most effective new degraders, **MA190** and **MA191** were most effective in inducing late apoptosis (Figure 4b). After 72 h, all degraders evoked equal levels of apoptosis (Figure 4c). We additionally tested these degraders in MOLM-13 cells which carry FLT3-ITD and FLT3 from a patient with AML after initial myelodysplastic syndrome. These were hardly affected by the degraders after 24 h (Figure 4d). After 72 h, **MA49**, **MA190**, and **MA191** were the most potent inducers of late apoptosis (Figure 4e). Within our novel PROTAC panel, the most potent elimination of FLT3-ITD by **MA190** and **MA191** (Figure 4a) translates into the most effective induction of late apoptosis in AML cells (Figure 4b–e).

To verify that **MA190** and **MA191** trigger the depletion of FLT3-ITD and a breakdown of its downstream signaling in both MV4-11 and MOLM-13 cells, we used immunoblotting. Furthermore, we evaluated if the elimination of FLT3-ITD is inherent to the PROTAC activities of **MA190** and **MA191**, or due to their kinase inhibitor moieties using **MA68**. We could verify that **MA190** and **MA191**, but not their underlying kinase inhibitor **MA68**, eliminate FLT3-ITD in MV4-11 cells (Figure 4f) and in MOLM-13 cells (Figure 4g). The loss of FLT3-ITD was linked to a reduction of STAT5 and AKT, their phosphorylation, and the auto-phosphorylation of FLT3-ITD in both AML cell systems (Figure 4f,g).

These findings on superior activities of two novel PROTACs for FLT3-ITD encourage additional analyses of these agents.

### 2.6. Proteomic Analysis of PROTAC MA191

#### 2.6.1. Proteomic Profiling of MA191 in MOLT-4 Cells

**Figure 5:**
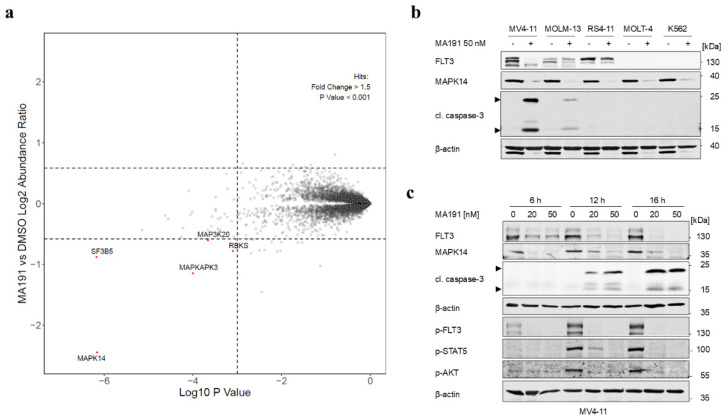
(**a**) Volcano blot for the global protein expression profiles of MOLT-4 cells treated with 50 nM MA191 for 5 h. (**b**) Immunoblot shows FLT3, MAPK14, cleaved caspase-3, and β-actin as loading control (cl., cleaved; indicated as arrows; −, untreated; +, treated with 50 nM MA191 for 24 h). (**c**) MV4-11 cells were incubated with 20–50 nM MA191 for 6–16 h. Immunoblots were performed as indicated. Immunoblot data are representative for at least two independent repetitions.

Proteome analyses can uncover unexpected targets of PROTACs. Since AML cells with FLT3-ITD are massively damaged by **MA191**, we applied it to MOLT-4 cells and analyzed them by proteomics. MOLT-4 leukemia cells express low levels of wild-type FLT3 [46], which can explain why FLT3 does not appear as a hit in this assay. The proteome analyses unraveled a striking downregulation of MAPK14 (Figure 5a).

#### 2.6.2. Dual Degradation of FLT3-ITD and MAPK14 by MA191 in AML Cells 

We could verify that **MA191** induces the degradation of MAPK14 in immunoblot analyses (Figure 5b). Moreover, **MA191** also depleted MAPK14 in FLT3 negative K562 chronic myeloid leukemia cells [45], in RS4-11 acute T cell lymphatic leukemia cells with wild-type FLT3, and in MV4-11 and MOLM-13 cells (Figure 5b). These data show that the downregulation of hyperactive FLT3-ITD and MAPK14 by **MA191** are independent processes. Analysis of activated caspase-3 demonstrated that the reduction of MAPK14 by **MA191** did not induce apoptosis in cells with wild-type FLT3. This biochemical marker of apoptosis was specifically seen in AML cells with FLT3-ITD (Figure 5b). 

A time course analysis of MV4-11 cells that were exposed to 20–50 nM **MA191** for 6 h, 12 h, and 16 h showed a time- and concentration-dependent depletion of FLT3-ITD and MAPK14, and an associated activation of caspase-3 (Figure 5c). The rapid elimination of FLT3-ITD by **MA191** after 6 h (Figure 5c) shows that **MA191** acts more rapidly than **MA49** which requires longer time periods to deplete FLT3-ITD [30]. 

**Figure 6:**
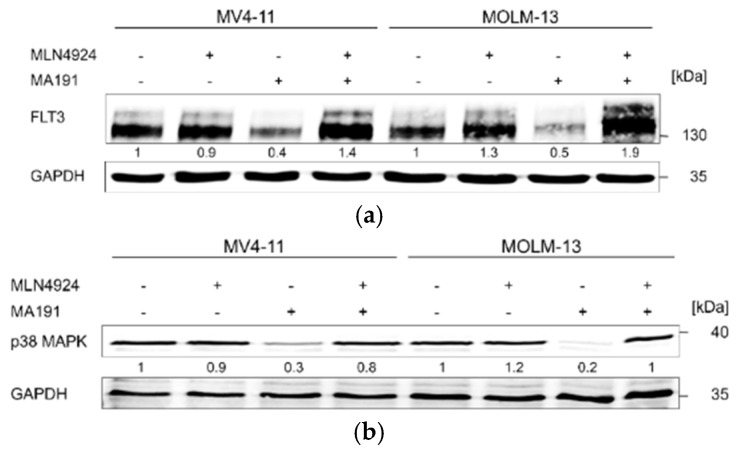
Rescuing protein degradation by the neddylation inhibitor MLN4924**.** MV4-11 and MOLM-13 cells were treated with 50 nM MA191 ± 3 µM MLN4924 for 6 h and analyzed by immunoblot for FLT3 (**a**) as well as for MAPK14 (**b**). The protein levels of GAPDH were determined to verify equal loading of samples. The data are representative for the outcome of two independent experiments in each cell line; +, treated; −, untreated; and kDa, molecular weight in kilodalton.

We then investigated whether the degradation is regulated via the CRL2^VHL^ E3 ubiquitin ligase complex [47]. Neddylation is a process in which the ubiquitin-like protein NEDD8 is covalently attached to a target protein. This post-translational modification leads to conformational changes and multivalent interactions of the CRL2^VHL^ complex, which accelerate the ubiquitination of substrate proteins [47]. The role of CRL2^VHL^ can therefore be examined using the neddylation inhibitor MLN4924. We applied MLN4924 in combination with **MA191** to MV4-11 and MOLM-13 cells. MLN4924 prevented the **MA191**-mediated degradation of FLT3-ITD as well as MAPK14 in both AML cell types (Figure 6a,b). These data confirm the anticipated proteasomal degradation of FLT3-ITD through VHL.

**Figure 7:**
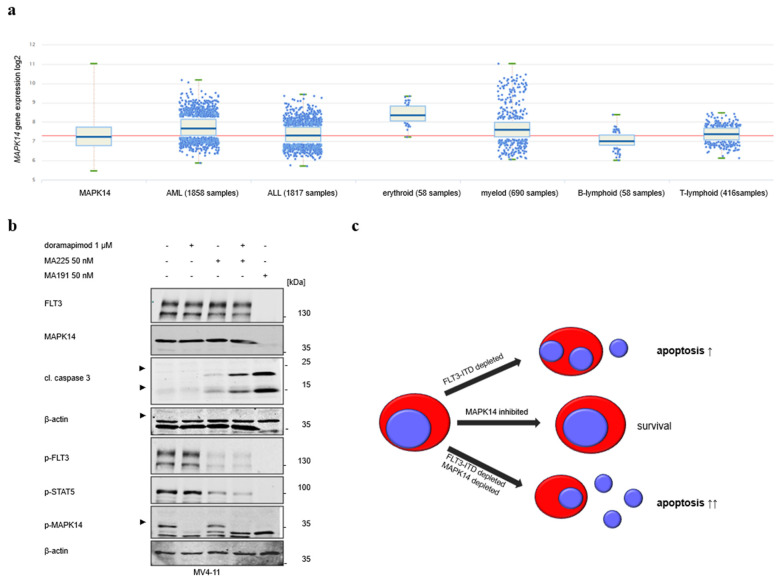
Targeting MAPK14 augments anti-leukemic effects of an inhibition of FLT3-ITD. (**a**) The graphs show the mRNA levels of MAPK14 in leukemia cells and normal blood cells. (**b**) Immunoblotting revealed the expression of the indicated proteins and their phosphorylation (p-) or cleavage (cl.; indicated by arrows); β-actin was used to ensure equal sample loading. All immunoblots show data from at least two independent repetitions. (**c**) Model for how inhibition of various signaling pathways affects AML cells (red, cytoplasm, blue, nucleus). The blue small circles represent fragmented DNA that is released from apoptotic cells; ↑, increased; ↑↑, more strongly increased.

We asked if the depletion of MAPK14 by **MA191** depletes a factor that is upregulated in leukemia cells. Analyzing the Hemap database (http://hemap.uta.fi/), we noted on average higher levels of MAPK14 in leukemia cells compared to normal myeloid cells and B-cells (Figure 7a).

We aimed to delineate if the FLT3 inhibitor part of **MA191** acts as a MAPK14 inhibitor in MV4-11 cells. We used the phosphorylation of FLT3-ITD and the phosphorylation of MAPK14 as markers for their activation. As a negative control for **MA191**, we used the negative control **MA225**, which contains the inactive stereoisomer of the VHL ligand. Consistent with them having the same FLT3 inhibitor backbone, both **MA191** and **MA225** inhibited the phosphorylation of FLT3-ITD and its downstream signaling to STAT5 strongly. Unlike **MA191**, **MA225** did not reduce the levels of FLT3-ITD and MAPK14. This translated into a weak inhibition of phosphorylated MAPK14 by **MA225** and a loss of p-MAPK14 together with MAPK14 in cells that were exposed to **MA191** (Figure 7b). This finding is consistent with the detection of MAPK14 in the kinase selectivity profiling approach (Figure 3; MAPK14 is termed p38α therein).

We then evaluated if the elimination of FLT3-ITD and MAPK14 has superior effects to a sole inhibition of FLT3-ITD in MV4-11 cells. We used the MAPK14 inhibitor doramapimod in combination with **MA225** to mimic the effects of **MA191** (i.e., inhibition of FLT3-ITD and MAPK14). Doramapimod inhibited p-MAPK14 without depleting MAPK14 and doramapimod did not affect FLT3-ITD and its activity (Figure 7b). Immunoblotting for activated caspase-3 as an apoptosis marker disclosed that the co-inhibition of FLT3-ITD and MAPK14 was superior to the sole inhibition of FLT3-ITD or MAPK14. Since doramapimod did not induce caspase-3 cleavage (Figure 7b), this observation suggests that the inhibition of MAPK14 augments pro-apoptotic effects of FLT3-ITD inhibition, and that we do not only observe combined toxicity. Furthermore, the lack of caspase-3 activation by doramapimod is consistent with the lack of this apoptosis marker in leukemia cells without FLT3-ITD (Figure 7b). The usefulness of a combined inhibition of FLT3-ITD and MAPK14 through depletion may also be effective against leukemia stem cells that require cytokines signaling through MAPK14 [48]. Further studies are necessary to test this idea.

### 2.7. Cytotoxicity Against HEK239 Cells

The most promising PROTACs were screened for their potential cytotoxicity in the human epithelial kidney cell line (HEK293) and were compared to their parent inhibitor **MA68** and the reference PROTAC **MA49**. Whereas the inhibitor **MA68** and the PROTAC **MA49** showed cytotoxic effects at a high concentration of 50 µM the novel PROTACs **MA190** and **MA191** showed no cytotoxic effects against HEK293 cells (Table 2). 

### 2.8. Physicochemical Properties

In addition to the degradation and determination of the effects on AML cancer cells, we also determined some physicochemical parameters to assess whether the compounds are suitable for further in vivo studies. Nephelometric measurements were performed to determine the water solubility of some compounds. We selected the inhibitor **MA68** and the PROTAC **MA191** as references. The nephelometric technique has been previously described as a high-throughput method for the solubility determination of compounds in the solvent of interest [49,50]. This technique measures the intensity of scattered light when visible light passes through a suspension of the tested compound. Relative turbidity is an indication of the insolubility of compounds. The inhibitor **MA68** as well as the potent PROTAC **MA191** showed good solubility in the tested PBS buffer (45.8 and 47.7 µM, respectively) which also allows cellular and in vivo studies in the necessary concentration ranges.

### 2.9. Stability Testing

An important aspect in the development of PROTACs is testing chemical stability. On the one hand, we tested all PROTACs produced for their stability under cellular assay conditions. For this purpose, 10 µM solutions of the PROTACs and reference inhibitor were incubated for 72 h at 37 °C with the assay medium DMEM. The remaining amount of the compounds was determined by HPLC after 6, 12, 24, 48 and 72 h. The results of the stability studies are shown in Table S2 (Supplementary Information). All tested PROTACs exhibit very good chemical stability and no degradation products among the 72 h of incubation. 

**Figure 8:**
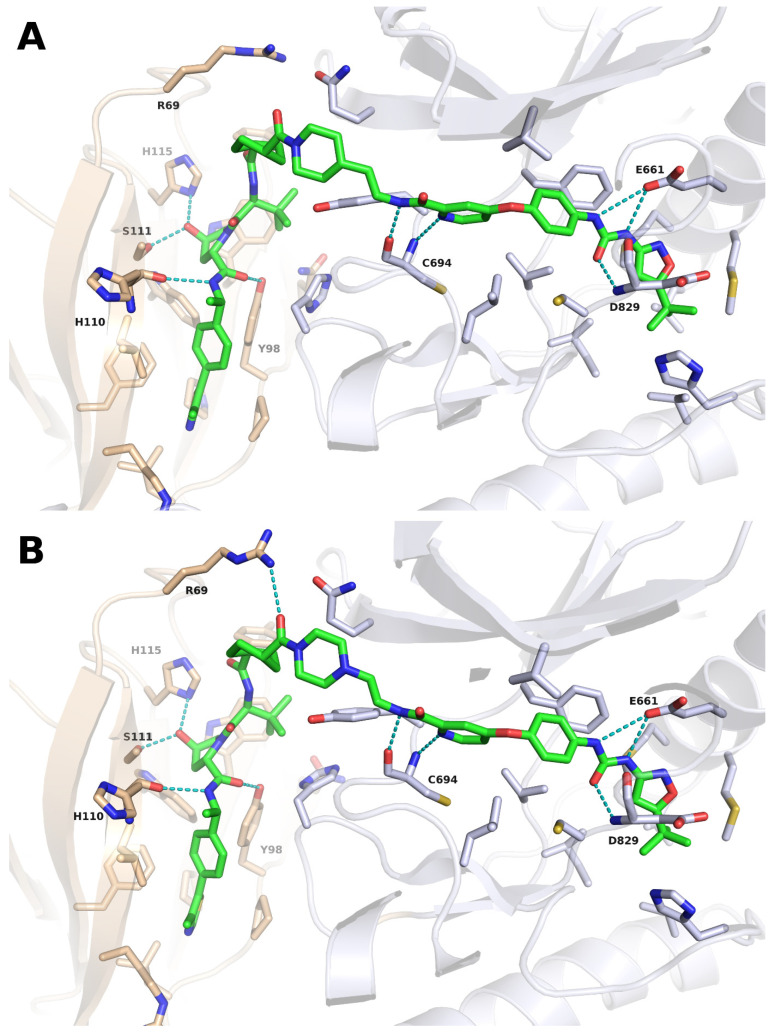
Docking poses of PROTACs (**A**) MA190 and (**B**) MA191 in the FLT3-VHL complex model. Interacting residues of FLT3 are colored orange, interacting residues of VHL are colored tan and the PROTAC is colored green. Hydrogen bonds (≤2.5 Å) are shown as cyan dashed lines.

Another aspect we tested for selected PROTACs, and the reference inhibitor is the stability in human plasma. PROTACs **MA190** and **MA191** and their parent inhibitor **MA68** (10 μM, final DMSO concentration 1%) were incubated with 100 μL pooled human plasma at 37 °C. The remaining amount of the incubated compound was tested for 6 h using a HPLC method. The reference inhibitor **MA68** and PROTACs **MA190** and **MA191** showed high plasma stability over the 6 h period (Table 3, Figure 8). The carried-out stability tests show that both PROTACs **MA190** and **MA191** exhibit high chemical stability in assay medium and in human plasma and could be used for cellular and in vivo testing. The further detailed biological characterization of **MA191** and the successful in vivo testing can be found in a study submitted in parallel [51].

### 2.10. Microsomal Stability

With the development of novel PROTACs with high degradation potency, we aimed to enhance also metabolic stability. Given that reducing linker flexibility has been shown to improve metabolic stability [31], therefore we developed **MA190** and **MA191** with rigidified linkers. Both the optimized PROTACs as well as the previously developed **MA49** were tested for metabolic stability in mouse liver microsomes. **MA49** was found to be almost completely degraded after 2 h (85.0%), whereas **MA190** and **MA191** showed hardly any degradation (8.0% and 10.8%, respectively) (Table 4). Thus, PROTAC **MA190** and **MA191** represent promising stable compounds for future in vivo studies.

### 2.11. Docking Study

To analyze the formation of the ternary complexes of the dual-specific PROTACs, we generated the complexes for the most active PROTACs at FLT3 and MAPK14 in silico. For the FLT3 kinase, we have described the generation of the ternary complex for the PROTAC **MA49** in a recently reported work and validated the methods on available crystal structures [35]. Recent reports showing that different active PROTACs can occupy identical ternary complexes [52,53,54] provided the impetus to dock the newly synthesized PROTACs of this study into the originally developed model of VHL-MA49-FLT3. We employed as in our previous studies induced-fit docking using program MOE [35,55]. For the docked PROTACs (**MA73**, **MA74**, **MA77**, **MA78**, **MA190**, and **MA191**) that all contained the same FLT3 inhibitor warhead, binding poses were obtained in which both ligands (FLT3 and VHL) interacted with their respective binding pockets in a manner analogous to **MA49** (Figure S1, Supplementary Information). The more rigid PROTACs **MA190** and **MA191** were also able to adopt a conformation that allowed interaction in both binding pockets. In the ternary complex models of **MA190** and **MA191**, both PROTACs spanned the active pockets of both proteins with relatively elongated linker conformations (Figure 8). The *N*-methyl-picolinamide group of the FLT3 ligand formed two hydrogen bonds with the hinge region at Cys694. The urea group of PROTACs formed two hydrogen bonds with FLT3 residues Glu661 and Asp829. In the VHL ligand, the R-hydroxy group of the pyrrolidine ring formed two hydrogen bonds with the VHL residues Ser111 and His115. In the neighboring amide, the oxygen atom was hydrogen-bonded to the hydroxy group of Tyr98, while the amino group formed a hydrogen bond with the carbonyl oxygen of His110. In addition, the phenyl ring of the VHL ligand was in close proximity to the phenyl ring of Tyr98, resulting in a π-stacking interaction (Figure 8). These interactions can also be found in the crystal structures of VHL-PROTACs (such as BRD4-VHL and PDB ID 5T35 [52]).

To model the MAPK14-MA191-VHL ternary complex, we first docked **MA68** into the ATP-binding site of MAPK14 using Glide molecular docking (Figure S2a, Supplementary Information). The top-ranked pose of **MA68** exhibited the typical type II inhibitor binding mode within the MAPK14 binding pocket. The *N*-methyl-picolinamide moiety of **MA68** is oriented toward the hinge region near the solvent-exposed area, forming a hydrogen bond with Met109. The urea oxygen and phenyl ring extended toward the DFG-out motif, forming a hydrogen bond with Asp168 and π-stacking interactions with Phe169, respectively (Figure S2a, Supplementary Information). The resulting pose of **MA68** closely matched the crystallographic binding mode of the type II MAPK14 inhibitor DP-1376 (PDB ID: 3NNU) (Figure S2b, Supplementary Information). Subsequently, the docked **MA68** pose at MAPK14, along with the VHL-Ligand 3 complex, was used to generate the ternary structure of **MA190**/**MA191** via the MOE Method 4B (Figure S2c,d, Supplementary Information).

**Figure 9:**
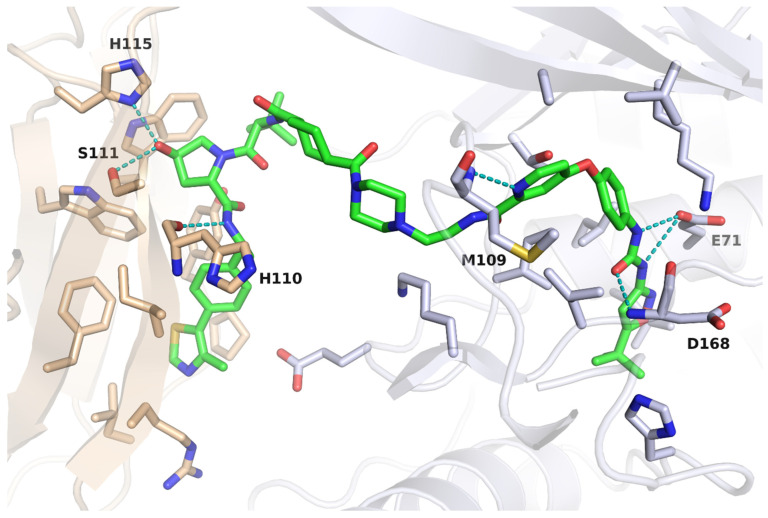
Docking poses of PROTACs MA191 in the MAPK14-VHL complex model. Interacting residues of MAPK14 are colored tan, interacting residues of VHL are colored white and the PROTAC is colored green. Hydrogen bonds (≤2.5 Å) are shown as cyan dashed lines.

The ternary complex of **MA191** with MAPK14-VHL resembles the structure of the corresponding complex with FLT3-VHL (Figure 9). The interaction of the MA68-inhibitor moiety shows the conserved H-bridge bonds with the hinge region (Met109) and the back pocket (Glu71, Asp168) of MAPK14, and also the VHL ligand binds as in the described crystal structures of VHL. Although the rigid linker (4-ethylpiperazin-1-yl)(4-methylcyclohexyl)methanone of **MA191** does not form a direct hydrogen or salt bridge with MAPK14 or VHL, it is able to optimally bridge the distance between the two binding pockets/ligand parts. Interestingly, the conformation of the PROTACs in FLT3 and MAPK14 is very similar and thus also the position of the kinases in relation to the VHL protein. 

## 3. Materials and Methods

### 3.1. Chemistry 

Materials and reagents were purchased from Sigma-Aldrich Co., Ltd. (Darmstadt, Germany) and abcr GmbH (Karlsruhe, Germany). All solvents were analytically pure. Thin-layer chromatography was carried out on aluminum sheets coated with silica gel 60 F254 (Merck, Darmstadt, Germany). For medium-pressure liquid chromatography (MPLC), Biotage SNAP ultra-HP-sphere 25 μm columns containing silica gel were used. Dichloromethane (DCM): methanol (MeOH) mixtures was used as elution systems for MPLC. Purity was measured by UV absorbance at 254 nm. The HPLC consisted of a LiChrosorb^®^ RP-18 (5 μm) 100–4.6 Merck column (Merck, Darmstadt, Germany), two LC-10AD pumps, a SPD-M10A VP PDA detector, and a SIL-HT autosampler, all from the manufacturer Shimadzu (Kyoto, Japan). The absorption spectra were recorded with a SPD-M10A diode array detector Shimadzu spectrophotometer (Kyoto, Japan). Mass spectrometry analyses were performed with a Finnigan MAT710C (Thermo Separation Products, SanJose, CA, USA) for the ESI MS spectra and with a LTQ (linear ion trap) Orbitrap XL hybrid mass spectrometer (Thermo Fisher Scientific, Bremen, Germany) for the HRMS-ESI (high-resolution mass spectrometry) spectra. For the HRMS analyses, the signal for the isotopes with the highest prevalence was given and calculated. NMR spectra were taken on a Varian Inova 400 (Agilent Technologies Deutschland GmbH, Waldbronn, Germany) using deuterated DMSO as solvent. Chemical shifts were referenced to the residual solvent signals. The following abbreviations and formulas for solvents and reagents were used: ethyl acetate (EtOAc), N,N-dimethylformamide (DMF), dimethyl sulfoxide (DMSO), methanol (MeOH), tetrahydrofuran (THF), water (H_2_O), dichloromethane (DCM), N,N-diisopropylethylamine (DIPEA), O-(7-azabenzotriazol-1-yl)-N,N,N′,N′-tetramethyluronium-hexafluorphosphate (HATU) and hydrochloric acid (HCl), trifluoroacetic acid (TFA).

#### 3.1.1. General Synthetic Methods

##### Method I: Hydrolysis of Methyl Ester

To a stirred suspension of the appropriate methyl ester (10.0 mmol, 1.0 equiv.) in THF: H_2_O (1:1) (10 mL), LiOH·H_2_O (2.1 g, 50.0 mmol, 5.0 equiv.) was added, and the mixture was stirred at RT for 3–6 h. The pH value of the mixture was adjusted to 5. The resulting solid was filtrated and washed with water to provide the corresponding carboxylic acid, which required no further purification. 

##### Method II: Hydrolysis of *tert*-Butyl Ester

The appropriate *tert*-butyl ester was dissolved at 0 °C in dry DCM (5 mL), then TFA (5 mL) was added. The reaction mixture was stirred at RT for 3 h. The solvent was evaporated to dryness to provide the corresponding carboxylic acid.

##### Method III: Amide Coupling

A solution of the appropriate carboxylic acid derivative (1.0 mmol, 1.0 equiv.), HATU (0.38 g, 1.0 mmol, 1.0 equiv.) and DIPEA (0.65 g, 5.0 mmol, 5.0 equiv.) in DMF (5 mL) was stirred at RT for 10 min., then the corresponding amine derivative (1.1 mmol, 1.1 equiv.) was added. The reaction mixture was stirred at RT for 3–7 h. After completion of the reaction as indicated by TLC, water was added, and the mixture was extracted using ethyl acetate. The combined organic layer was washed with an aqueous 1 M ammonium chloride solution, followed by an aqueous 1 M sodium bicarbonate solution and brine. The combined organic extract was dried over anhydrous sodium sulfate, and the organic layer was filtered, then evaporated under reduced pressure to yield the crude amide, which was purified by MPLC using DCM: MeOH (3–10% MeOH). 

##### Method IV: N-Boc-Deprotection

The appropriate *N*-Boc-protected amine derivative was dissolved at 0 °C in dry DCM (5 mL), and then TFA (5 mL) was added. The reaction mixture was stirred at RT for 30–60 min. The solvent was evaporated to dryness to provide the corresponding amine derivative as a TFA salt.

#### 3.1.2. Synthesis and Characterization Data of Key Intermediates and Final Compounds

##### Synthesis and Characterization of Intermediates **3**–**12**

Intermediates **3**–**5** were synthesized according to the reported procedures [56]. The compounds were confirmed with ^1^HNMR and mass.

Methyl 4-(4-(3-(3-(trifluoromethyl)phenyl)ureido)phenoxy)picolinate (**7**): The phenyl isocyanate derivative **6** (1.47 g, 6.0 mmol, 1.0 equiv.) in DCM (15 mL) was added dropwise to a stirred solution of methyl 4-(4-aminophenoxy)picolinate (**5**) (1.13 g, 6.0 mmol, 1.0 equiv.) in DCM (15 mL) at 0 °C. Then, the reaction mixture was allowed to stir at RT for 3 h. The formed precipitate was filtered, washed with DCM (5 mL), and dried under vacuum to obtain the corresponding urea derivatives, which required no further purification. The product was obtained as a white solid (yield: 77%). ^1^H NMR (400 MHz, DMSO-d_6_) δ 9.01 (s, 1H), 8.87 (s, 1H), 8.51 (d, *J* = 5.6 Hz, 1H), 7.95 (s, 1H), 7.57–7.48 (m, 3H), 7.45 (t, *J* = 7.9 Hz, 1H), 7.36 (d, *J* = 2.4 Hz, 1H), 7.25 (d, *J* = 7.4 Hz, 1H), 7.15–7.07 (m, 3H), 3.77 (s, 3H).
4-(4-(3-(3-(Trifluoromethyl)phenyl)ureido)phenoxy)picolinic acid (**8**): Intermediate **7** underwent ester hydrolysis according to method I to obtain the corresponding carboxylic acid **8** which was obtained as a white solid (yield: 90%). MS *m*/*z*: 416.16 [M − H]^−^. ^1^H NMR (400 MHz, DMSO-d_6_) δ 13.23 (s, 1H), 9.03 (s, 1H), 8.88 (s, 1H), 8.48 (d, *J* = 5.6 Hz, 1H), 7.94 (s, 1H), 7.50–7.40 (m, 3H), 7.43 (t, *J* = 7.9 Hz, 1H), 7.33 (d, *J* = 2.0 Hz, 1H), 7.23 (d, *J* = 7.5 Hz, 1H), 7.10–7.02 (m, 3H).

Intermediate **12** and the inhibitor **MA68** were synthesized according to the reported procedures [30]. The compounds were confirmed with ^1^HNMR and mass.

##### Synthesis and Characterization of Compound **19** (**MA74**)

The carboxylic acid intermediate **12** reacted with the PEG linker **16** according to method III, followed by *tert*-butyl ester hydrolysis using method II to obtain the corresponding carboxylic acid derivative **17**. Intermediate **17** was reacted with the VHL amine **18** according to method III to obtain compound **19**.

3-(2-(2-(4-(4-(3-(5-(*tert*-Butyl)isoxazol-3-yl)ureido)phenoxy)picolinamido)ethoxy)ethoxy)-propanoic acid (**17**) was obtained as a white solid (yield: 49% over 2 steps). MS *m*/*z*: 578.35 [M + Na]^+^. ^1^H NMR (400 MHz, DMSO-d_6_) δ 12.11 (s, 1H), 9.56 (s, 1H), 9.01 (s, 1H), 8.79–8.59 (m, 1H), 8.57–8.41 (m, 1H), 7.65–7.51 (m, 2H), 7.42–7.27 (m, 1H), 7.20–7.02 (m, 3H), 6.49 (s, 1H), 3.68–3.37 (m, 10H), 2.40 (t, *J* = 6.4 Hz, 2H), 1.28 (s, 9H).
4-(4-(3-(5-(*tert*-Butyl)isoxazol-3-yl)ureido)phenoxy)-*N*-(2-(2-(3-(((*S*)-1-((2*S*,4*R*)-4-hydroxy-2-(((*S*)-1-(4-(4-methylthiazol-5-yl)phenyl)ethyl)carbamoyl)pyrrolidin-1-yl)-3,3-dimethyl-1-oxobutan-2-yl)amino)-3-oxopropoxy)ethoxy)ethyl)picolinamide **19** (**MA74**) was obtained as a white solid (yield: 55%). ^1^H NMR (400 MHz, DMSO-d_6_) δ 9.51 (s, 1H), 8.95 (s, 1H), 8.92 (s, 1H), 8.66 (t, *J* = 5.8 Hz, 1H), 8.49 (d, *J* = 5.6 Hz, 1H), 8.33 (d, *J* = 7.8 Hz, 1H), 7.81 (d, *J* = 9.3 Hz, 1H), 7.58–7.51 (m, 2H), 7.43–7.30 (m, 5H), 7.18–7.11 (m, 3H), 6.48 (s, 1H), 5.06 (d, *J* = 3.5 Hz, 1H), 4.94–4.84 (m, 1H), 4.50 (d, *J* = 9.4 Hz, 1H), 4.40 (t, *J* = 8.0 Hz, 1H), 4.25 (s, 1H), 3.62–3.37 (m, 12H), 2.53–2.48 (m, 1H), 2.42 (s, 3H), 2.32 (dt, *J* = 14.6, 6.1 Hz, 1H), 2.03–1.94 (m, 1H), 1.77 (ddd, *J* = 12.9, 8.4, 4.6 Hz, 1H), 1.34 (d, *J* = 7.0 Hz, 3H), 1.27 (s, 9H), 0.90 (s, 9H). ^13^C NMR (101 MHz, DMSO-d_6_) δ 180.6, 171.0, 170.3, 169.9, 166.4, 163.7, 158.8, 152.6, 151.9, 150.9, 148.4, 148.2, 145.1, 137.2, 131.5, 130.1, 129.2, 126.8, 121.9, 120.8, 114.6, 109.2, 92.9, 69.9, 69.19, 69.15, 67.4, 59.0, 56.8, 56.7, 48.1, 39.1, 38.1, 36.1, 35.7, 32.9, 28.8, 26.85, 26.81, 26.78, 22.8, 16.4. HRMS *m*/*z*: 1004.432 [M + Na]^+^; calculated C_50_H_63_O_10_N_9_NaS^+^: 1004.4316. HPLC: rt 15.31 min (purity 98.74%).

##### Synthesis and Characterization of Compounds **21a**–**e** and **22a**–**e**

The appropriate carboxylic acid **8** or **12** was reacted with the corresponding amine **20a**–**c** according to method III, followed by methyl ester hydrolysis using method I to obtain the corresponding carboxylic acid derivatives **21a**–**e**. The carboxylic acids obtained were further reacted with the VHL amine building block **18** according to method III to afford compounds **22a**–**e**.

6-(4-(4-(3-(3-(Trifluoromethyl)phenyl)ureido)phenoxy)picolinamido)hexanoic acid (**21a**) was obtained as a white solid (yield: 63% over 2 steps). MS *m*/*z*: 529.49 [M − H]^−^. ^1^H NMR (400 MHz, DMSO-d_6_) δ 9.15 (s, 1H), 9.01 (s, 1H), 8.78 (t, *J* = 6.1 Hz, 1H), 8.49 (d, *J* = 5.7 Hz, 1H), 8.01 (s, 1H), 7.63–7.55 (m, 3H), 7.50 (t, *J* = 7.9 Hz, 1H), 7.40 (t, *J* = 2.0 Hz, 1H), 7.32–7.26 (m, 1H), 7.19–7.10 (m, 3H), 3.23 (q, *J* = 8 Hz, 2H), 2.17 (t, *J* = 7.4 Hz, 2H), 1.56–1.41 (m, 4H), 1.37–1.27 (m, 2H).
8-(4-(4-(3-(3-(Trifluoromethyl)phenyl)ureido)phenoxy)picolinamido)octanoic acid (**21b**) was obtained as a white solid (yield: 55% over 2 steps). MS *m*/*z*: 559.43 [M + H]^+^. ^1^H NMR (400 MHz, DMSO-d_6_) δ 9.15 (s, 1H), 9.00 (s, 1H), 8.76 (t, *J* = 6.0 Hz, 1H), 8.49 (d, *J* = 5.7 Hz, 1H), 8.01 (s, 1H), 7.62–7.55 (m, 3H), 7.50 (t, *J* = 7.9 Hz, 1H), 7.39 (t, *J* = 6.5 Hz, 1H), 7.29 (d, *J* = 7.7 Hz, 1H), 7.19–7.10 (m, 3H), 3.23 (q, *J* = 8 Hz, 2H), 2.15 (t, *J* = 7.4 Hz, 2H), 1.48 –1.42 (m, 2H), 1.29–1.17 (m, 8H).
6-(4-(4-(3-(5-(*tert*-Butyl)isoxazol-3-yl)ureido)phenoxy)picolinamido)hexanoic acid (**21c**) was obtained as a white solid (yield: 60% over 2 steps). MS *m*/*z*: 508.27 [M − H]^−^. ^1^H NMR (400 MHz, DMSO-d_6_) δ 9.54 (s, 1H), 8.97 (s, 1H), 8.77 (t, *J* = 6.1 Hz, 1H), 8.49 (d, *J* = 5.7 Hz, 1H), 7.63–7.54 (m, 2H), 7.44–7.38 (m, 1H), 7.21–7.08 (m, 3H), 6.48 (d, *J* = 5.5 Hz, 1H), 3.24 (q, *J* = 8 Hz, 2H), 2.21–2.11 (m, 2H), 1.56–1.42 (m, 4H), 1.32–1.20 (m, 11H).
7-(4-(4-(3-(5-(*tert*-Butyl)isoxazol-3-yl)ureido)phenoxy)picolinamido)heptanoic acid (**21d**) was obtained as a white solid (yield: 62% over 2 steps). MS *m*/*z*: 546.34 [M + Na]^+^. ^1^H NMR (400 MHz, DMSO-d_6_) δ 11.92 (s, 1H), 9.58 (s, 1H), 9.04 (s, 1H), 8.74 (t, *J* = 6.1 Hz, 1H), 8.49 (d, *J* = 5.7 Hz, 1H), 7.63–7.49 (m, 2H), 7.36 (d, *J* = 2.6 Hz, 1H), 7.20–7.08 (m, 3H), 6.49 (s, 1H), 3.22 (q, *J* = 8 Hz, 2H), 2.16 (t, *J* = 7.4 Hz, 2H), 1.46 (dt, *J* = 14.6, 7.2 Hz, 4H), 1.32–1.20 (m, 13H).
8-(4-(4-(3-(5-(*tert*-Butyl)isoxazol-3-yl)ureido)phenoxy)picolinamido)octanoic acid (**21e**) was obtained as a white solid (yield: 60% over 2 steps). ^1^H NMR (400 MHz, DMSO-d_6_) δ 9.61 (s, 1H), 9.21 (s, 1H), 8.78 (t, *J* = 5.4 Hz, 1H), 8.50 (d, *J* = 5.7 Hz, 1H), 7.58 (t, *J* = 8.3 Hz, 2H), 7.42 (s, 1H), 7.21–7.10 (m, 3H), 6.49 (s, 1H), 3.23 (q, *J* = 8 Hz, 2H), 2.16 (t, *J* = 7.3 Hz, 2H), 1.55–1.38 (m, 4H), 1.37–1.20 (m, 15H).
*N*-(6-(((*S*)-1-((2*S*,4*R*)-4-Hydroxy-2-(((*S*)-1-(4-(4-methylthiazol-5-yl)phenyl)ethyl)carbamoyl)-pyrrolidin-1-yl)-3,3-dimethyl-1-oxobutan-2-yl)amino)-6-oxohexyl)-4-(4-(3-(3-(trifluoromethyl)phenyl)ureido)phenoxy)picolinamide **22a** (**MA43**) was obtained as a white solid (yield 52%). ^1^H NMR (400 MHz, DMSO-d_6_) δ 9.10 (s, 1H), 8.99–8.90 (m, 2H), 8.72 (t, *J* = 6.0 Hz, 1H), 8.49 (d, *J* = 5.6 Hz, 1H), 8.33 (d, *J* = 7.8 Hz, 1H), 8.00 (s, 1H), 7.74 (d, *J* = 9.2 Hz, 1H), 7.64–7.54 (m, 3H), 7.50 (t, *J* = 7.9 Hz, 1H), 7.45–7.26 (m, 6H), 7.19–7.08 (m, 3H), 5.06 (d, *J* = 3.6 Hz, 1H), 4.94–4.84 (m, 1H), 4.48 (d, *J* = 9.3 Hz, 1H), 4.40 (t, *J* = 8.0 Hz, 1H), 4.26 (s, 1H), 3.58 (s, br, 2H), 3.27–3.17 (m, 2H), 2.43 (s, 3H), 2.27–2.16 (m, 1H), 2.14 –2.03 (m, 1H), 2.03–1.93 (m, 1H), 1.82–1.71 (m, 1H), 1.55–1.40 (m, 4H), 1.35 (d, *J* = 7.0 Hz, 3H), 1.24–1.20 (m, 2H), 0.91 (s, 9H). ^13^C NMR (101 MHz, DMSO-d_6_) δ 172.4, 171.1, 170.1, 166.5, 163.5, 153.0, 152.9, 151.9, 150.8, 148.2, 145.1, 141.0, 137.7, 131.6, 130.4, 130.1, 129.8, 129.3, 126.8, 122.3, 121.9, 120.8, 118.6, 114.6, 114.5, 109.2, 69.2, 59.0, 56.8, 56.7, 48.1, 39.2, 38.1, 35.6, 35.3, 29.3, 26.9, 26.5, 25.6, 22.8, 16.4. HRMS *m*/*z*: 979.377 [M + Na]^+^; calculated C_49_H_55_O_7_N_8_F_3_NaS^+^: 979.3764. HPLC: rt 12.55 min (purity 97.16%).
*N*-(8-(((*S*)-1-((2*S*,4*R*)-4-Hydroxy-2-(((*S*)-1-(4-(4-methylthiazol-5-yl)phenyl)ethyl)carbamoyl)-pyrrolidin-1-yl)-3,3-dimethyl-1-oxobutan-2-yl)amino)-8-oxooctyl)-4-(4-(3-(3-(trifluoromethyl)phenyl)ureido)phenoxy)picolinamide **22b** (**MA42**) was obtained as a white solid (yield: 57%). ^1^H NMR (400 MHz, DMSO-d_6_) δ 9.08 (s, 1H), 8.97–8.89 (m, 2H), 8.73 (t, *J* = 6.1 Hz, 1H), 8.48 (d, *J* = 5.6 Hz, 1H), 8.33 (d, *J* = 7.8 Hz, 1H), 8.17 (s, 1H), 8.01 (s, 1H), 7.74 (d, *J* = 9.3 Hz, 1H), 7.58–7.52 (m, 3H), 7.50 (t, *J* = 7.9 Hz, 1H), 7.43–7.26 (m, 5H), 7.19–7.08 (m, 3H), 5.06 (d, *J* = 3.6 Hz, 1H), 4.88 (tt, *J* = 11.7, 5.9 Hz, 1H), 4.49 (d, *J* = 9.3 Hz, 1H), 4.41 (t, *J* = 8.0 Hz, 1H), 4.26 (d, *J* = 2.9 Hz, 1H), 3.66–3.51 (m, 3H), 3.23 (q, *J* = 8 Hz, 2H), 3.12–3.06 (m, 2H), 2.43 (s, 3H), 2.28–2.14 (m, 1H), 2.14 –2.03 (m, 1H), 2.02–1.93 (m, 1H), 1.83–1.78 (m, 1H), 1.54–1.40 (m, 4H), 1.35 (d, *J* = 7 Hz, 3H), 1.30–1.14 (m, 3H), 0.91 (s, 9H). ^13^C NMR (101 MHz, DMSO-d_6_) δ 172.5, 171.0, 170.0, 166.4, 163.5, 153.0, 152.9, 151.9, 150.8, 148.17, 148.16, 145.1, 141.0, 137.6, 131.5, 130.3, 130.1, 129.8, 129.2, 126.8, 122.3, 121.9, 120.8, 118.5, 114.64, 114.60, 114.5, 109.2, 69.2, 59.0, 56.8, 56.7, 54.0, 48.1, 42.3, 38.2, 35.6, 29.5, 29.0, 28.2, 26.9, 22.8, 18.5, 17.2, 16.4, 12.9. HRMS *m*/*z*: 985.426 [M + H]^+^; calculated C_51_H_60_O_7_N_8_F_3_S^+^: 985.4257. HPLC: rt 12.95 min (purity 97.37%).
4-(4-(3-(5-(*tert*-Butyl)isoxazol-3-yl)ureido)phenoxy)-*N*-(6-(((*S*)-1-((2*S*,4*R*)-4-hydroxy-2-(((*S*)-1-(4-(4-methylthiazol-5-yl)phenyl)ethyl)carbamoyl)pyrrolidin-1-yl)-3,3-dimethyl-1-oxobutan-2-yl)amino)-6-oxohexyl)picolinamide **22c** (**MA78**) was obtained as a white solid (yield: 57%). ^1^H NMR (400 MHz, DMSO-d_6_) δ 9.52 (s, 1H), 9.00–8.90 (m, 2H), 8.72 (t, *J* = 6.0 Hz, 1H), 8.48 (t, *J* = 5.2 Hz, 1H), 8.33 (d, *J* = 7.8 Hz, 1H), 7.74 (d, *J* = 9.3 Hz, 1H), 7.60–7.52 (m, 2H), 7.46–7.32 (m, 5H), 7.19–7.10 (m, 3H), 6.49 (s, 1H), 5.06 (d, *J* = 3.6 Hz, 1H), 4.95–4.85 (m, 1H), 4.48 (d, *J* = 9.3 Hz, 1H), 4.40 (t, *J* = 8.0 Hz, 1H), 4.24 (t, *J* = 9.7 Hz, 1H), 3.65–3.53 (m, 2H), 3.27–3.18 (m, 2H), 2.43 (s, 3H), 2.27–2.16 (m, 1H), 2.14–2.04 (m, 1H), 2.03– 1.93 (m, 1H), 1.83–1.72 (m, 1H), 1.55–1.41 (m, 4H), 1.35 (d, *J* = 7.0 Hz, 3H), 1.27 (s, 9H), 1.25–1.16 (m, 2H), 0.88 (s, 9H). ^13^C NMR (101 MHz, DMSO-d_6_) δ 180.6, 172.4, 171.0, 170.0, 166.4, 163.5, 158.8, 152.9, 151.9, 150.8, 148.4, 148.2, 145.1, 137.2, 131.5, 130.1, 129.2, 126.8, 121.9, 120.8, 114.5, 109.2, 92.9, 69.2, 59.0, 56.8, 56.7, 48.1, 38.1, 35.6, 35.3, 32.9, 29.3, 28.8, 27.2, 26.92, 26.86, 26.5, 25.6, 22.8, 16.4. HRMS *m*/*z*: 958.4263 [M + Na]^+^; calculated C_49_H_61_O_8_N_9_NaS^+^: 958.4262. HPLC: rt 15.48 min (purity 95.13%).
4-(4-(3-(5-(*tert*-Butyl)isoxazol-3-yl)ureido)phenoxy)-*N*-(7-(((*S*)-1-((2*S*,4*R*)-4-hydroxy-2-(((*S*)-1-(4-(4-methylthiazol-5-yl)phenyl)ethyl)carbamoyl)pyrrolidin-1-yl)-3,3-dimethyl-1-oxobutan-2-yl)amino)-7-oxoheptyl)picolinamide **22d** (**MA77**) was obtained as a white solid (yield: 55%). ^1^H NMR (400 MHz, DMSO-d_6_) δ 9.50 (s, 1H), 8.97–8.88 (m, 2H), 8.72 (t, *J* = 6.0 Hz, 1H), 8.48 (d, *J* = 5.6 Hz, 1H), 8.32 (d, *J* = 7.8 Hz, 1H), 7.73 (d, *J* = 9.3 Hz, 1H), 7.57–7.52 (m, 2H), 7.37 (dt, *J* = 8.2, 7.0 Hz, 5H), 7.18–7.08 (m, 3H), 6.48 (s, 1H), 5.05 (d, *J* = 3.5 Hz, 1H), 4.92–4.83 (m, 1H), 4.48 (d, *J* = 9.3 Hz, 1H), 4.39 (t, *J* = 8.1 Hz, 1H), 4.25 (d, *J* = 3.0 Hz, 1H), 3.62–3.54 (m, 2H), 3.21 (dd, *J* = 13.6, 6.6 Hz, 2H), 2.42 (s, 3H), 2.25–2.16 (m, 1H), 2.13–2.03 (m, 1H), 2.01–1.92 (m, 1H), 1.76 (ddd, *J* = 12.8, 8.5, 4.7 Hz, 1H), 1.52–1.38 (m, 4H), 1.34 (d, *J* = 7.0 Hz, 3H), 1.27 (s, 9H), 1.24–1.16 (m, 4H), 0.90 (s, 9H). ^13^C NMR (101 MHz, DMSO-d_6_) δ 180.6, 172.5, 171.0, 170.1, 166.4, 163.5, 158.8, 152.9, 151.9, 150.8, 148.4, 148.2, 145.1, 137.2, 131.5, 130.1, 129.2, 126.8, 121.9, 120.8, 114.5, 109.2, 92.9, 69.2, 59.0, 56.8, 56.7, 48.1, 38.1, 35.6, 35.3, 32.9, 29.5, 28.84, 28.79, 28.75, 26.93, 26.87, 26.6, 25.8, 22.8, 16.4. HRMS *m*/*z*: 972.4421 [M + Na]^+^; calculated C_50_H_63_O_8_N_9_NaS^+^: 972.4418. HPLC: rt 15.67 min (purity 95.03%).
4-(4-(3-(5-(*tert*-Butyl)isoxazol-3-yl)ureido)phenoxy)-*N*-(8-(((*S*)-1-((2*S*,4*R*)-4-hydroxy-2-(((*S*)-1-(4-(4-methylthiazol-5-yl)phenyl)ethyl)carbamoyl)pyrrolidin-1-yl)-3,3-dimethyl-1-oxobutan-2-yl)amino)-8-oxooctyl)picolinamide **22e** (**MA73**) was obtained as a white solid (yield: 59% yield). ^1^H NMR (400 MHz, DMSO-d_6_) δ 9.51 (s, 1H), 8.98–8.90 (m, 2H), 8.72 (t, *J* = 6.1 Hz, 1H), 8.47 (d, *J* = 5.6 Hz, 1H), 8.32 (d, *J* = 7.8 Hz, 1H), 7.73 (d, *J* = 9.3 Hz, 1H), 7.58–7.52 (m, 2H), 7.43–7.38 (m, 2H), 7.37–7.30 (m, 3H), 7.18–7.09 (m, 3H), 6.48 (s, 1H), 5.05 (d, *J* = 3.6 Hz, 1H), 4.94–4.85 (m, 1H), 4.48 (d, *J* = 9.3 Hz, 1H), 4.39 (t, *J* = 8.0 Hz, 1H), 4.25 (d, *J* = 2.8 Hz, 1H), 3.62–3.53 (m, 2H), 3.22 (dd, *J* = 13.5, 6.7 Hz, 2H), 2.42 (d, *J* = 3.7 Hz, 3H), 2.27–2.14 (m, 1H), 2.12–2.02 (m, 1H), 2.01–1.93 (m, 1H), 1.77 (ddd, *J* = 12.9, 8.4, 4.7 Hz, 1H), 1.52–1.39 (m, 4H), 1.34 (d, *J* = 7.0 Hz, 3H), 1.26 (s, 9H), 1.22 (s, 6H), 0.89 (s, 9H). ^13^C NMR (101 MHz, DMSO-d_6_) δ 180.6, 172.5, 171.0, 170.0, 166.4, 163.5, 158.8, 152.9, 151.9, 150.8, 148.4, 148.2, 145.1, 137.2, 131.5, 130.1, 129.2, 126.8, 126.7, 121.9, 120.8, 114.5, 109.2, 92.9, 69.2, 59.0, 56.8, 56.7, 48.1, 38.1, 35.6, 35.5, 35.3, 32.9, 29.5, 29.0, 28.9, 28.8, 26.91, 26.86, 26.78, 25.8, 22.8, 16.4. HRMS *m*/*z*: 986.458 [M + Na]^+^; calculated C_51_H_65_O_8_N_9_NaS^+^: 986.4575. HPLC: rt 15.87 min (purity 93.24%).

##### Synthesis and Characterization of Compounds **25**–**31a**,**b**

Firstly, *trans*-1,4-cyclohexanedicarboxylic acid monomethyl ester (**23**) was treated with the appropriate VHL amine **18** or **24** according to method III followed by alkaline ester hydrolysis according to method I to obtain the corresponding carboxylic acids **25** and **26**, respectively. Then, the carboxylic acid intermediate **12** reacted with mono-Boc protected amine **27a** or **27b** according to method III to get the Boc-protected intermediates **28a** and **28b**, respectively, followed by Boc-deprotection according to method IV to obtain intermediates **29a** and **29b**, respectively. Finally, intermediate **25** was reacted with intermediates **29a** and **29b**, respectively according to method III to get compounds **30a** and **30b**, respectively. Similarly, intermediate **26** was treated with intermediates **29a** or **29b** to get compounds **31a** and **31b**, respectively.

*trans*-4-(((*S*)-1-((2*S*,4*R*)-4-Hydroxy-2-(((*S*)-1-(4-(4-methylthiazol-5-yl)phenyl)-ethyl)carbamoyl)pyrrolidin-1-yl)-3,3-dimethyl-1-oxobutan-2-yl)carbamoyl)cyclohexane carboxylic acid (**25**) was obtained as a white solid (yield: 95%). MS *m*/*z*: 599.3 [M + H]^+^. ^1^H NMR (400 MHz, DMSO-d_6_) δ 11.98 (s, 1H), 8.96 (s, 1H), 8.34 (d, *J* = 7.7 Hz, 1H), 7.65 (d, *J* = 9.1 Hz, 1H), 7.41 (d, *J* = 8.3 Hz, 2H), 7.36 (d, *J* = 8.3 Hz, 2H), 5.06 (s, 1H), 4.95–4.86 (m, 1H), 4.47 (d, *J* = 9.3 Hz, 1H), 4.40 (t, *J* = 8.0 Hz, 1H), 4.26 (s, 1H), 3.62–3.52 (m, 2H), 2.43 (s, 3H), 2.37–2.26 (m, 1H), 2.18–2.06 (m, 1H), 2.03–1.97 (m, 1H), 1.92–1.84 (m, 2H), 1.81–1.72 (m, 2H), 1.66 (d, *J* = 11.4 Hz, 1H), 1.41–1.19 (m, 7H), 0.91 (s, 9H).
*trans*-4-(((*S*)-1-((2*R*,4*S*)-4-Hydroxy-2-(((*S*)-1-(4-(4-methylthiazol-5-yl)phenyl)ethyl)carbamoyl)pyrrolidin-1-yl)-3,3-dimethyl-1-oxobutan-2-yl)carbamoyl)-cyclohexane carboxylic acid (**26**) was obtained as a white solid (yield: 92%). MS *m*/*z*: 599.1 [M + H]^+^. ^1^H NMR (400 MHz, DMSO-d_6_) δ 11.97 (s, 1H), 8.95 (s, 1H), 8.06 (d, *J* = 7.9 Hz, 1H), 7.76 (d, *J* = 8.1 Hz, 1H), 7.52–7.34 (m, 4H), 5.07 (s, 1H), 4.92–4.83 (m, 1H), 4.40–4.32 (m, 2H), 4.31–4.24 (m, 1H), 3.73 (dd, *J* = 10.5, 5.4 Hz, 1H), 3.47 (dd, *J* = 10.4, 3.9 Hz, 1H), 2.44 (s, 3H), 2.36–2.25 (m, 1H), 2.11–2.01 (m, 1H), 1.98–1.91 (m, 2H), 1.88–1.77 (m, 2H), 1.76–1.70 (m, 1H), 1.66–1.58 (m, 1H), 1.36–1.18 (m, 7H), 0.93 (s, 9H).
*tert*-Butyl 4-(2-(4-(4-(3-(5-(*tert*-butyl)isoxazol-3-yl)ureido)phenoxy)picolinamido)ethyl)-piperidine-1-carboxylate (**28a**) was obtained as a pale yellow solid (yield: 56%). MS *m*/*z*: 629.1 [M + Na]^+^. ^1^H NMR (400 MHz, DMSO-d_6_) δ 9.52 (s, 1H), 8.93 (s, 1H), 8.76 (t, *J* = 6.0 Hz, 1H), 8.49 (d, *J* = 5.6 Hz, 1H), 7.56 (d, *J* = 8.9 Hz, 2H), 7.36 (d, *J* = 2.6 Hz, 1H), 7.22–7.08 (m, 3H), 6.49 (s, 1H), 3.87 (d, *J* = 12.1 Hz, 2H), 3.29–3.23 (m, 2H), 2.87 (s, 1H), 2.65 (s, 2H), 1.64 (d, *J* = 12.3 Hz, 2H), 1.43 (d, *J* = 5.4 Hz, 2H), 1.36 (s, 9H), 1.25 (s, 9H), 1.03–0.87 (m, 2H).
*tert*-Butyl 4-(2-(4-(4-(3-(5-(*tert*-butyl)isoxazol-3-yl)ureido)phenoxy)picolinamido)ethyl)-piperazine-1-carboxylate (**28b**) was obtained as a pale yellow solid (yield 50%). MS *m*/*z*: 630.1 [M + Na]^+^. ^1^H NMR (500 MHz, DMSO-d_6_) δ 9.40 (s, 1H), 9.14–8.83 (m, 1H), 8.70 (t, *J* = 5.6 Hz, 1H), 8.50 (d, *J* = 5.6 Hz, 1H), 7.62–7.53 (m, 2H), 7.37 (d, *J* = 2.6 Hz, 1H), 7.19–7.09 (m, 3H), 6.49 (s, 1H), 3.38 (dd, *J* = 12.3, 6.4 Hz, 2H), 3.29–3.23 (m, 4H), 2.48–2.45 (m, 2H), 2.38–2.32 (m, 4H), 1.38 (s, 9H), 1.29 (s, 9H).
4-(2-(4-(4-(3-(5-(*tert*-Butyl)isoxazol-3-yl)ureido)phenoxy)picolinamido)ethyl)piperidin-1-ium 2,2,2-trifluoroacetate (**29a**) was obtained as a yellow solid (yield: 92%). MS *m*/*z*: 507.0 [M + H]^+^. ^1^H NMR (400 MHz, DMSO-d_6_) δ 9.74 (s, 1H), 9.24 (s, 1H), 8.82 (t, *J* = 6.0 Hz, 1H), 8.57 (d, *J* = 9.2 Hz, 1H), 8.49 (d, *J* = 5.6 Hz, 1H), 8.25 (d, *J* = 9.8 Hz, 1H), 7.66–7.51 (m, 2H), 7.37 (d, *J* = 2.6 Hz, 1H), 7.21–7.08 (m, 3H), 6.49 (s, 1H), 3.36–3.14 (m, 4H), 2.95–2.72 (m, 4H), 1.83 (d, *J* = 13.1 Hz, 2H), 1.61–1.39 (m, 3H), 1.25 (s, 9H).
4-(2-(4-(4-(3-(5-(*tert*-Butyl)isoxazol-3-yl)ureido)phenoxy)picolinamido)ethyl)piperazin-1-ium 2,2,2-trifluoroacetate (**29b**) was obtained as a yellow solid (yield: 90%). MS *m*/*z*: 508.0 [M + H]^+^_._^1^H NMR (400 MHz, DMSO-d_6_) δ 9.81 (s, 1H), 9.37 (s, 1H), 9.33 (s, 1H), 9.08 (t, *J* = 6.0 Hz, 1H), 8.51 (d, *J* = 5.6 Hz, 1H), 7.59 (d, *J* = 9.0 Hz, 3H), 7.38 (d, *J* = 2.6 Hz, 1H), 7.19–7.15 (m, 1H), 7.14 (d, *J* = 8.9 Hz, 2H), 6.48 (s, 1H), 3.62 (dd, *J* = 11.6, 5.7 Hz, 2H), 3.56–3.26 (m, 10H), 1.27 (s, 9H).
4-(4-(3-(5-(*tert*-Butyl)isoxazol-3-yl)ureido)phenoxy)-*N*-(2-(1-(*trans*-4-(((*S*)-1-((2*S*,4*R*)-4-hydroxy-2-(((*S*)-1-(4-(4-methylthiazol-5-yl)phenyl)ethyl)carbamoyl)pyrrolidin-1-yl)-3,3-dimethyl-1-oxobutan-2-yl)carbamoyl)cyclohexanecarbonyl)piperidin-4-yl)ethyl)picolinamide **30a** (**MA190**) was obtained as a white solid (yield: 53%). ^1^H NMR (400 MHz, DMSO-d_6_) δ 9.56 (s, 1H), 9.01 (s, 1H), 8.96 (s, 1H), 8.77 (t, *J* = 5.9 Hz, 1H), 8.49 (d, *J* = 5.6 Hz, 1H), 8.34 (d, *J* = 7.7 Hz, 1H), 7.63 (d, *J* = 9.3 Hz, 1H), 7.56 (d, *J* = 8.9 Hz, 2H), 7.41 (d, *J* = 8.3 Hz, 2H), 7.39–7.31 (m, 3H), 7.19–7.10 (m, 3H), 6.49 (s, 1H), 5.07 (s, 1H), 4.95–4.86 (m, 1H), 4.48 (d, *J* = 9.3 Hz, 1H), 4.41 (t, *J* = 8.0 Hz, 1H), 4.32 (d, *J* = 12.8 Hz, 1H), 4.26 (s, 1H), 3.88 (d, *J* = 11.6 Hz, 1H), 3.64–3.49 (m, 2H), 2.96–2.85 (m, 1H), 2.57–2.50 (m, 1H), 2.43 (s, 3H), 2.36–2.26 (m, 1H), 2.04–1.92 (m, 1H), 1.82–1.70 (m, 3H), 1.69–1.57 (m, 4H), 1.54–1.40 (m, 5H), 1.39–1.30 (m, 6H), 1.28 (s, 9H), 1.24–1.14 (m, 2H), 1.05–0.95 (m, 1H), 0.91 (s, 9H), 0.87–0.80 (m, 1H). ^13^C NMR (101 MHz, DMSO-d_6_) δ 180.6, 175.2, 173.3, 171.1, 170.0, 166.4, 163.6, 158.8, 152.9, 151.9, 150.8, 148.4, 148.2, 145.1, 137.2, 131.5, 130.1, 129.3, 126.8, 122.0, 120.8, 114.5, 109.2, 92.9, 69.2, 59.0, 56.7, 56.6, 48.1, 45.3, 43.2, 41.7, 39.0, 38.1, 36.8, 36.1, 35.8, 33.5, 33.1, 32.9, 29.5, 28.8, 28.2, 26.9, 22.9, 16.4. HRMS *m*/*z*: 1109.5265 [M + Na]^+^; calculated C_58_H_74_O_9_N_10_NaS^+^: 1109.5259. HPLC: rt 15.41 min (purity 99.08%).
4-(4-(3-(5-(*tert*-Butyl)isoxazol-3-yl)ureido)phenoxy)-*N*-(2-(4-(*trans*-4-(((*S*)-1-((2*S*,4*R*)-4-hydroxy-2-(((S)-1-(4-(4-methylthiazol-5-yl)phenyl)ethyl)carbamoyl)pyrrolidin-1-yl)-3,3-dimethyl-1-oxobutan-2-yl)carbamoyl)cyclohexanecarbonyl)piperazin-1-yl)ethyl)picolinamide **30b** (**MA191**) was obtained as a white solid (yield: 55%). ^1^H NMR (400 MHz, DMSO-d_6_) δ 9.55–9.50 (m, 1H), 8.95 (d, *J* = 7.1 Hz, 2H), 8.70 (t, *J* = 5.8 Hz, 1H), 8.49 (d, *J* = 5.6 Hz, 1H), 8.34 (d, *J* = 7.8 Hz, 1H), 7.63 (d, *J* = 9.2 Hz, 1H), 7.56 (d, *J* = 8.9 Hz, 2H), 7.41 (d, *J* = 8.3 Hz, 2H), 7.38–7.29 (m, 3H), 7.19–7.10 (m, 3H), 6.49 (s, 1H), 5.07 (d, *J* = 3.5 Hz, 1H), 4.90 (dt, *J* = 14.2, 6.9 Hz, 1H), 4.48 (d, *J* = 9.3 Hz, 1H), 4.41 (t, *J* = 8.0 Hz, 1H), 4.26 (s, 1H), 3.65–3.50 (m, 2H), 3.50–3.34 (m, 6H), 2.58–2.50 (m, 1H), 2.46–2.42 (m, 4H), 2.40–2.30 (m, 5H), 2.04–1.93 (m, 1H), 1.84–1.69 (m, 2H), 1.63 (d, *J* = 10.9 Hz, 3H), 1.49–1.30 (m, 7H), 1.28 (s, 9H), 1.24–1.18 (m, 1H), 0.91 (s, 9H). ^13^C NMR (101 MHz, DMSO-d_6_) δ 180.6, 175.2, 173.5, 171.1, 170.0, 166.4, 163.6, 158.8, 152.7, 151.9, 150.9, 148.4, 148.2, 145.1, 137.2, 131.5, 130.1, 129.3, 126.8, 121.9, 120.8, 114.6, 109.2, 92.9, 69.2, 59.0, 57.0, 56.6, 52.9, 48.1, 43.2, 41.5, 38.8, 38.2, 36.6, 35.8, 32.9, 29.5, 28.9, 28.8, 28.6, 28.2, 26.9, 22.9, 16.4. HRMS *m*/*z*: 1088.540 [M + H]^+^; calculated C_57_H_74_O_9_N_11_S^+^: 1088.5392. HPLC: rt 15.08 min (purity 95.20%).
4-(4-(3-(5-(*tert*-Butyl)isoxazol-3-yl)ureido)phenoxy)-*N*-(2-(1-(*trans*-4-(((*S*)-1-((2*R*,4*S*)-4-hydroxy-2-(((*S*)-1-(4-(4-methylthiazol-5-yl)phenyl)ethyl)carbamoyl)pyrrolidin-1-yl)-3,3-dimethyl-1-oxobutan-2-yl)carbamoyl)cyclohexanecarbonyl)piperidin-4-yl)ethyl)picolinamide **31a** (**MA224**) was obtained as a white solid (yield: 55%) ^1^H NMR (400 MHz, DMSO-d_6_) δ 9.52 (s, 1H), 8.95 (s, br, 2H), 8.77 (t, *J* = 5.9 Hz, 1H), 8.49 (d, *J* = 5.6 Hz, 1H), 8.07 (d, *J* = 7.6 Hz, 1H), 7.74 (d, *J* = 8.1 Hz, 1H), 7.56 (d, *J* = 9.0 Hz, 2H), 7.49–7.38 (m, 4H), 7.36 (d, *J* = 2.6 Hz, 1H), 7.20–7.10 (m, 3H), 6.48 (s, 1H), 5.08 (d, *J* = 3.9 Hz, 1H), 4.93–4.83 (m, 1H), 4.43–4.34 (m, 2H), 4.33–4.24 (m, 2H), 3.81 (d, *J* = 11.9 Hz, 1H), 3.73 (dd, *J* = 10.4, 5.3 Hz, 1H), 3.47 (dd, *J* = 10.3, 3.9 Hz, 1H), 2.87 (t, *J* = 11.7 Hz, 1H), 2.45–2.36 (m, 4H), 2.31 (t, *J* = 11.4 Hz, 1H), 1.99–1.90 (m, 2H), 1.78–1.18 (m, 28H), 1.01–0.80 (m, 11H). ^13^C NMR (101 MHz, DMSO-d_6_) δ 180.6, 175.8, 173.2, 171.1, 170.0, 166.4, 163.6, 158.8, 152.9, 151.9, 150.8, 148.4, 148.2, 144.8, 137.2, 131.5, 130.1, 129.2, 127.0, 122.0, 120.8, 114.5, 109.2, 92.9, 68.9, 59.0, 57.0, 55.7, 48.0, 45.3, 43.0, 41.6, 38.9, 38.2, 36.8, 36.1, 35.0, 33.5, 33.1, 32.9, 32.0, 28.8, 26.9, 26.8, 23.0, 16.4. HRMS *m*/*z*: 1109.5267 [M + Na]^+^; calculated C_58_H_74_O_9_N_10_NaS^+^: 1109.5259. HPLC: rt 15.68 min (purity 98.02%).
4-(4-(3-(5-(*tert*-Butyl)isoxazol-3-yl)ureido)phenoxy)-*N*-(2-(4-(*trans*-4-(((*S*)-1-((2*R*,4*S*)-4-hydroxy-2-(((*S*)-1-(4-(4-methylthiazol-5-yl)phenyl)ethyl)carbamoyl)pyrrolidin-1-yl)-3,3-dimethyl-1-oxobutan-2-yl)carbamoyl)cyclohexanecarbonyl)piperazin-1-yl)ethyl)picolinamide **31b** (**MA225**) was obtained as a white solid (yield: 55%). ^1^H NMR (400 MHz, DMSO-d_6_) δ 9.53 (m, 1H), 8.95 (s, br, 2H), 8.69 (t, *J* = 5.8 Hz, 1H), 8.49 (d, *J* = 5.6 Hz, 1H), 8.08 (d, *J* = 8.2 Hz, 1H), 7.74 (d, *J* = 8.2 Hz, 1H), 7.58–7.52 (m, 2H), 7.49–7.38 (m, 4H), 7.36 (d, *J* = 5.2 Hz, 1H), 7.18–7.10 (m, 3H), 6.48 (s, 1H), 5.08 (d, *J* = 4.0 Hz, 1H), 4.93–4.83 (m, 1H), 4.43–4.35 (m, 2H), 4.29 (dd, *J* = 8.9, 4.6 Hz, 1H), 3.73 (dd, *J* = 10.4, 5.3 Hz, 1H), 3.47 (dd, *J* = 10.4, 3.9 Hz, 1H), 3.41–3.33 (m, 6H), 2.47–2.39 (m, 5H), 2.38–2.25 (m, 5H), 1.98–1.92 (m, 2H), 1.76–1.67 (m, 1H), 1.63–1.51 (m, 3H), 1.44–1.33 (m, 2H), 1.32 (d, *J* = 7.0 Hz, 3H), 1.30 –1.25 (m, 10H), 1.24–1.19 (m, 2H), 0.93 (s, 9H). ^13^C NMR (101 MHz, DMSO-d_6_) δ 180.6, 175.8, 173.4, 171.1, 169.9, 166.4, 163.6, 158.8, 152.7, 151.9, 150.9, 148.4, 148.2, 144.8, 137.2, 131.5, 130.1, 129.2, 127.0, 122.0, 120.8, 114.6, 109.2, 92.9, 68.9, 59.0, 57.0, 55.7, 52.9, 48.0, 43.0, 41.5, 38.8, 38.2, 36.6, 35.1, 32.9, 29.3, 28.8, 28.6, 28.4, 26.9, 26.8, 23.0, 16.4. HRMS *m*/*z*: 1088.5407 [M + H]^+^; calculated C_57_H_74_O_9_N_11_S^+^: 1088.5392. HPLC: rt 14.92 min (purity 99.32%).

### 3.2. Computational Methods

For the new FLT3-VHL PROTACs described in this work, a modeling approach was chosen that we have recently reported and used for the development of the prototype FLT3 PROTAC MA49 [35]. We used the developed model of the ternary FLT3-MA49-VHL complex [35] for induced-fit docking of the active PROTACs synthesized in this work (MA73, MA74, MA77, MA78, MA190 and MA191). The PROTACs were energy minimized using the LigPrep module implemented in Schrödinger version 2021 [57]. The predicted ionization states at pH 7.0, which resulted in protonation of the piperazine, were kept unchanged. Induced fit docking was carried out in MOE2019 and was constrained using a pharmacophore model for MA49 developed in the original study [35]. MOE-induced fit docking generated 1000 initial placement poses, of which the top 100 MOE-ranked poses were used for refinement. During refinement, the Generalized-Born Volume Integral/Weighted Surface Area (GB-VI/WSA) evaluation function was used. GB-VI/WSA is a force field-based scoring function that estimates the free energy of binding (kcal/mol) of PROTACs based on the placed pose [58].

For MAPK14 employed the same method as previously described for FLT3 [35] to model the MAPK14-**MA191**-VHL ternary complex. Consistent with our prior work, unbound MAPK14 and VHL monomers were retrieved from the Protein Data Bank (PDB) to resemble a realistic scenario in the absence of an experimental ternary complex structure. Specifically, we used VHL in complex with Ligand 3 (PDB ID: 5NVV [59]) and MAPK14 in complex with the type II inhibitor DP-1376 (PDB ID: 3NNU [60]), both prepared using the Protein Preparation Wizard in Schrödinger version 2021 [57]. Preparation steps included adding hydrogen atoms, completing missing side chains and loops, and removing ions and water molecules. Protonation states and tautomeric forms were optimized at pH 7.0 using PROPKA version 3.1 followed by restrained energy minimization with the OPLS4 force field [57].

To model the MAPK14-MA191-VHL ternary complex, we first docked **MA68** into the ATP-binding site of MAPK14 using Glide molecular docking (Figure S2a, Supplementary Information). The top-ranked pose of **MA68** exhibited the typical type II inhibitor binding mode within the MAPK14 binding pocket. The docking protocol was validated by redocking the co-crystallized type II MAPK14 inhibitor, DP-1376 (PDB ID: 3NNU). The resulting pose closely matched the crystallographic binding mode, with an RMSD of 0.37 Å (Figure S2b, Supplementary Information). Finally, **MA68** was docked into the MAPK14 binding pocket using the Glide module (standard precision mode), and the resulting MAPK14-**MA68** complex, together with VHL-Ligand 3 complex and the **MA191** PROTAC, served as input for ternary complex modeling using Method 4B in MOE. **MA190** and **MA191** were docked into the MAPK14-**MA191**-VHL ternary complex model by using the induced fit docking method in MOE as described for FLT3 above.

### 3.3. Kinase Binding Affinity Testing Using KINOMEscan

Kinase-tagged T7 phage strains were prepared in an *E. coli* host derived from the BL21 strain. *E. coli* were grown to log-phase and infected with T7 phage and incubated with shaking at 32 °C until lysis. The lysates were centrifuged and filtered to remove cell debris. The remaining kinases were produced in HEK-293 cells and subsequently tagged with DNA for qPCR detection. Streptavidin-coated magnetic beads were treated with biotinylated small molecule ligands for 30 min at room temperature to generate affinity resins for kinase assays. The liganded beads were blocked with excess biotin and washed with blocking buffer (SeaBlock (Pierce), 1% BSA, 0.05% Tween 20, 1 mM DTT) to remove unbound ligand and to reduce non-specific binding. Binding reactions were assembled by combining the kinase, liganded affinity beads, and test compounds in 1x binding buffer (20% SeaBlock, 0.17× PBS, 0.05% Tween 20, 6 mM DTT). Test compounds were prepared as 111× stocks in 100% DMSO. K_d_ values were determined using an 11-point 3-fold compound dilution series with three DMSO control points. All compounds for K_d_ measurements are distributed by acoustic transfer (non-contact dispensing) in 100% DMSO. The compounds were then diluted directly into the assays such that the final concentration of DMSO was 0.9%. All reactions performed in polypropylene 384-well plate. Each was a final volume of 0.02 mL. The assay plates were incubated at room temperature with shaking for 1 h and the affinity beads were washed with wash buffer (1× PBS, 0.05% Tween 20). The beads were then re-suspended in elution buffer (1× PBS, 0.05% Tween 20, 0.5 μM nonbiotinylated affinity ligand) and incubated at room temperature with shaking for 30 min. The kinase concentration in the eluates was measured by qPCR.

### 3.4. Kinase Selectivity Profiling Using NanoBRET^TM^

The CELLinib128 assay was performed by CELLinib GmbH, Frankfurt (Germany) as described previously [42,43]. In brief: Full-length kinase ORF (Promega, Madison, WI, USA) cloned in frame with a NanoLuc-vector was transfected into HEK293T cells in Opti-MEM without phenol red (Life Technologies, Carlsbad, CA, USA) at a density of 2 × 10^5^ cells/mL using FuGENE HD (Promega, E2312) and proteins were allowed to express for 20 h. For CDKs, where indicated below, an excess of an activator without an NLuc-fusion was cotransfected. For the control compounds, serially diluted inhibitor (10-point dose–response from 18 µM to 1.0 nM)—and for the 1-shot test compounds the indicated screening concentration—and NanoBRET^TM^ Kinase Tracer (as indicated in the table below at close to the previously determined tracer K_D,app_) were pipetted into white 1536-well plates (Greiner 782074, Greiner Bio-One, Kremsmünster, Austria). The system was allowed to equilibrate at 37 °C for 2 h and 5% CO_2_ prior to BRET measurements. For PKCs, a chemical activator PMA was added in a final concentration of 1 µM was added together with test compounds and tracers (Promega, V1171). To measure BRET, NanoBRET^TM^ NanoGlo substrate + extracellular NanoLuc inhibitor (Promega, N2160) were added as per the manufacturer’s protocol, and filtered luminescence was measured (450 nm BP filter (donor) and 610 nm LP filter (acceptor)). For the 1-shot test compounds, data were normalized to the tracer control and the background control or control compound plateau and an average and SD were calculated. If the resulting average was smaller than −5% or higher than 120%, the respective value was set to these constraints and no SD was calculated. For the control compounds competitive displacement data were normalized to the tracer Ctrl and the background Ctrl and then plotted and fitted using GraphPad Prism 10 software using a normalized 3-parameter curve fit with the following equation: Y = 100/(1 + 10^X−LogIC50^).

### 3.5. Cell Culture

The cell lines that we used are MV4-11 (AML cells, from a 10-year-old boy), MOLM-13 (AML cells, from a 20-year-old male), RS4-11 (ALL cells, from a 32-year-old female), MOLT-4 cells (ALL cells, from a 19-year-old, male), and K562 cells (CML cells, from a 53-year-old female). These cells were gifts from Prof. Frank Böhmer or Dr. Sebastian Drube, University of Jena, Germany (originally from the DSMZ, Braunschweig, Germany) [30]. Cells were kept at 37 °C, 5% CO_2_, and a humid atmosphere. Growth medium was RPMI-1640, 5–10% fetal bovine serum (FBS) and 1% penicillin/streptomycin (Sigma Aldrich, Munich, Germany). Cell lines were confirmed to be mycoplasma-free by MycoStrip (Invivogen, Toulouse, France).

### 3.6. Antibodies and Immunoblot

Immunoblotting was performed as recently described by us in [30]. Antibodies: anti-FLT3 (ab245116, 1:300), anti-AKT (ab32505, 1:2000), anti-p38 delta/MAPK13 + p38 alpha/MAPK14 (ab31828, 1:1000) from Abcam, Cambridge, UK; anti-phospho-FLT3 (Tyr591) (3461, 1:1000), anti-cleaved caspase-3 (Asp175) (9661, 1:1000), anti-phospho-Akt (Ser473) (9271, 1:1000), anti-phospho-p38 MAPK (Thr180/Tyr182) (9215, 1:1000) from Cell Signaling Technology, Leiden, The Netherlands; anti-STAT5 (33-5900, 1:1000), anti-phospho-STAT5 (Tyr694) (MA5-14973, 1:1000) from Thermo Fisher, Dreieich, Germany; anti-beta-actin (sc-47778, 1:1000), vinculin (sc-73614, 1:1000) from Santa Cruz, Heidelberg, Germany. Secondary antibodies: HRP-coupled anti-mouse (7076; 1:2500) and anti-rabbit (7074; 1:2500) were purchased from Cell Signaling Technology, Leiden, The Netherlands. IRDye^®^ 680RD- (mouse: 925-68070; 1:10,000; rabbit: 925-68071; 1:10,000) or IRDye^®^ 800CW-coupled secondary antibodies (mouse: 925-32210, 1:10,000; rabbit: 925-32211, 1:10,000) were from Licor, Bad Homburg, Germany.

### 3.7. Global Proteomics in MOLT4 Cells

The proteomic study was carried out by the Fischer Lab Degradation Proteomics Initiative, Dana-Farber Cancer Institute, Boston, MA, USA.

#### 3.7.1. Boiler Plate Methods: Sample Preparation LFQ Quantitative Mass Spectrometry

MOLT4 cells were lysed using a buffer (50 mM NaCl, 8 M urea, 50 mM EPPS, pH 8.5, with phosphatase and protease inhibitors) and homogenized by bead beating (3 × 30 s at 2400 strokes/min). Protein concentration was determined using the Bradford assay. Fifty micrograms of protein per sample were reduced, alkylated, and precipitated using methanol/chloroform [61]. The dried protein pellet was resuspended in 4 M urea/50 mM HEPES (pH 7.4) and diluted to 1 M urea with 200 mM EPPS (pH 8). 

Proteins were digested with LysC and trypsin (1:50, enzyme:protein) for 12 h at 37 °C. Samples were acidified to pH 2–3 with formic acid and desalted using C18 solid-phase extraction. Peptides were dried, reconstituted in 0.1% formic acid, and prepared for LC-MS analysis. Analysis was performed using a TimsTOF HT coupled to a nanoElute LC pump via CaptiveSpray. Peptides were separated on a C18 column (25 cm × 75 µm, 1.6 µm) at 50 °C using a 50 min gradient (2–30% acetonitrile) with a flow rate of 250 nL/min.

To determine (1/K_0_) values, TIMS voltages were calibrated using three standard masses. For diaPASEF, py_diAID [62] was used to generate variable isolation windows aligned with the precursor density in the *m*/*z*–ion mobility space. Data acquisition was performed over 20 cycles with 3 mobility windows each (total of 60), covering 350–1250 *m*/*z* and 1/K_0_ from 0.6 to 1.45 V·s/cm^2^.

#### 3.7.2. LC-MS Data Analysis

diaPASEF raw data were analyzed using DIA-NN 1.8 in library-free mode, enabling peptide/protein identification, FDR control, protein assembly, and quantification through in silico digestion and deep learning-based predictions [63]. Analysis was performed against the SwissProt human database (January 2021). Standard directDIA settings were applied: tryptic digestion (2 missed cleavages), carbamidomethylation (C), oxidation (M), and FDR < 1%. Precursor quantification used Robust LC with RT-based cross-run normalization.

Proteins with low total abundance (<2000 × number of treatments) were excluded. Only proteins with at least 3 values in ≥4 replicates per treatment vs. DMSO comparison were retained [64]. Scaling and imputation of missing values were performed using in-house R scripts (R Development Core Team, 2014). Significant differences versus control were assessed using two-sided moderated *t*-tests via the limma package in R framework (R Development Core Team, 2014) [65].

### 3.8. Cytotoxicity Studies in HEK293 CELLS

To determine the cytotoxicity of the developed PORTACs on the human epithelial kidney, cell line HEK293 was used. HEK293 cells (DSMZ Braunschweig, ACC305) were incubated at 37 °C in a humidified incubator with 5% CO_2_ in Dulbecco’s Modified Eagle Medium (DMEM) supplemented with 10% FCS and 5 mM glutamine. Cells were seeded out at 1.5 × 10^3^ cells per well in a 96-well cell culture plate (TPP, Trasadingen, Switzerland). The compounds to be tested were added immediately to the medium at 50 µM. After 24 h, Alamar Blue reagent (Invitrogen, Carlsbad, CA, USA) was added according to the manufacturer’s instructions and incubated again for 21 h before samples were analyzed. Detection of viable cells which convert the resazurine reagent into the highly fluorescent resorufin was performed by using a FLUOstar OPTIMA microplate reader (BMG Labtec, Ortenberg, Germany) with the following filter set: Ex 530 nm/Em 590 nm. Measurements were performed in triplicate and data are means with standard deviation <12%. Daunorubicin was used as a positive control and an IC_50_ value of 12.55 ± 0.07 µM was measured.

### 3.9. Nephelometric Solubility Studies

First, equidistant and logarithmic pre-dilutions were prepared in a 96-well plate with round bottoms, using 25 mM stock solutions of compounds **MA68** and **MA191** in DMSO. Then, 5 μL of each pre-dilution was added to 245 μL of PBS buffer in a 96-well plate with straight bottoms. Quadruplets were prepared for the blank (DMSO + PBS), and final concentrations (one plate per compound) were tested. The final concentrations measured started from 500 μM to 0.78125 μM. A NEPHELOstarplus (BMG LABTECH GmbH, Ortenberg, Germany) was used to measure the solubility, and omega-data analysis software (version 3.42 R5) was used to analyze the results.

### 3.10. Non-Enzymatic Stability Testing

The developed compounds were diluted in the assay media consisting of a mixture of DMEM (50%), DMSO (10%) and acetonitrile (40%) at pH 7.4 and incubated at 37 °C for 72 h. The quantity of the compounds was measured after 6, 12, 24, 48, and 72 h by HPLC using an XTerra RP18 column (3.5 mm, 3.9 mm × 100 mm) from the manufacturer Waters (Milford, MA, USA) and two LC-10AD pumps, an SPD-M10AVP PDA detector, and an SIL-HT autosampler, all from the manufacturer Shimadzu (Kyoto, Japan).

### 3.11. Plasma Stability

To determine plasma stability, 10 μM of the given compound was incubated with 100 μL human pooled plasma for 5, 10, 30, 60, 120 and 360 min at 37 °C. The reactions were stopped by adding 300 μL of acetonitrile with subsequent plasma proteins sedimentation. The samples were subjected to ultra-centrifugation (5 min, 10,000 RPM/g) using modified PES 30K low protein binding centrifugal filter. The filtrate was then analyzed using HPLC to determine the available concentration of the compounds and also to determine and quantify potential degradation products. As HPLC system a LiChrosorb^®^ RP-18 (5 μm) 100–4.6 column from the manufacturer Merck, two LC-10AD pumps, a SPD-M10A VP PDA detector, and a SIL HT auto-sampler were used (all from the manufacturer Shimadzu, Kyoto, Japan). As mobile phase a gradient with increasing polarity composed of methanol/water/trifluoroacetic acid at a flow rate of 1 mL/min. UV absorbance was measured at 254 nm was used.

### 3.12. Metabolic Stability Assay in Mouse (CD-1) Liver Microsomes

The metabolic stability of selected compounds was evaluated using mouse (CD-1) liver microsomes. The test compounds were incubated at a concentration of 25 μM with microsomal preparations under NADPH-supplemented conditions, and the rate of parent compound depletion was determined over time. Diclofenac sodium (25 μM) was included as a positive control to confirm the validity and performance of the assay. 

The test compounds were dissolved in a suitable stock concentration (10 mM in DMSO). Twelve 1.5 mL microcentrifuge tubes were prepared for each test compound (for 0, 10, 20, 30, 60, and 120 min, including duplicate tubes) and the positive and negative controls. The final incubation volume for each tube is 700 µL. Incubations were carried out using 0.1 M potassium phosphate buffer (pH 7.4) (634 μL), mouse (CD-1) microsomes purchased from ThermoFisher 20 mg/mL (17 µL), 10 mM of test compound or positive control (7 μL), 1 M MgCl_2_ (7 µL), and 20 mM NADPH (35 μL). The mixtures, except NADPH, were pre-incubated for 10 min in a thermoshaker maintained at 37 °C, and reactions were initiated by the addition of the NADPH solution. The samples were mixed well by briefly vortexing and then incubated at 37 °C. The samples were collected at six time points (0, 10, 20, 30, 60, and 120 min) to monitor compound degradation. At each time point, 100 μL of the incubation mixture was withdrawn and immediately quenched with an equal volume of ice-cold acetonitrile to terminate enzymatic activity.

The quenched samples were vortexed and centrifuged at 10,000× *g* for 5 min at 4 °C. The supernatants were then put into HPLC vials for analysis. Chromatographic separation was performed using a Shimadzu HPLC system equipped with two LC-10AD pumps and an SIL-HAT autosampler. A LiChrosphere 100 RP-18e column (5 μm, 100 mm; Merck) was used as the stationary phase. The mobile phase consisted of a methanol–water gradient containing 0.05% trifluoroacetic acid (TFA). The injection volume was 20 μL, and analytes were detected using an SPD-M10A VP PDA UV-Vis detector (from the manufacturer Shimadzu, Kyoto, Japan) set at 254 or 280 nm.

The remaining concentration of the parent compound at each time point was determined, and the extent of metabolism (as substrate depletion) and % recovery of the test compounds and positive control were calculated.

## 4. Conclusions

In the current study, we focused on the development and optimization of novel PROTACs for the targeted degradation of the oncogenic FLT3-ITD in AML cells. Through systematic modification of the linker components based on the previously identified and characterized degrader **MA49**, we developed the more potent and chemically stable degraders **MA190** and **MA191**. These exhibit significantly stability in microsomal testing and enhanced degradation efficiency, which might be the due to their rigidified linker structures. **MA191** eliminates FLT3-ITD more rapidly and completely (>95%) than previously reported PROTACs. Notably, **MA191** additionally induces an unexpected but specific downregulation of MAPK14, a stress kinase signaling protein being overexpressed in leukemia cells. Interestingly, the novel PROTACs **MA190** and **MA191** do not show cytotoxic effects in human HEK293 cells. Thus, we disclose the first FLT3-ITD/MAPK14 co-targeting degrader with high microsomal stability. Functional cell studies demonstrate that the combined elimination of FLT3-ITD and MAPK14 by **MA191** triggers stronger apoptosis in AML cells than inhibition of either target alone. Immunoblots and proteomic analyses confirm the independence of these two effects. Furthermore, **MA190** and **MA191** exhibit improved kinase selectivity, high chemical and plasma stability, and good aqueous solubility. Such properties render them promising candidates for preclinical in vivo studies which we describe in a parallel study focusing on biological characterization [48]. These findings highlight the therapeutic potential of FLT3-targeted PROTAC **MA191** with dual target engagement and provide a promising foundation for in vivo studies.

## Figures and Tables

**Table 1:** Chemical structures of developed FLT3-ITD PROTACs.

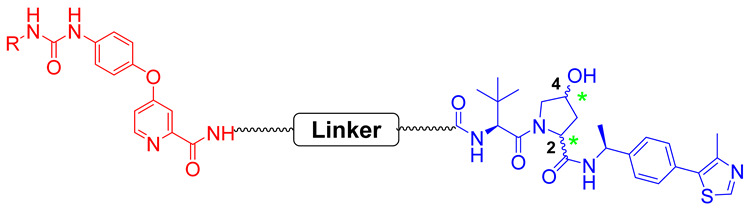						
Cpd. Id	R	Linker	VHL Ligand	% FLT3-ITD Binding at 1 µM	% MAPK- 14 Binding at 1 µM	% FLT3-ITD Degradation at 50 nM
**19** (**MA74**)	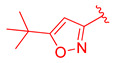	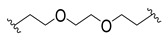	2S, 4R	89 ± 2	n.d.	90
**22a** (**MA43**)	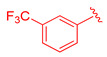	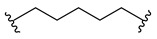	2S, 4R	n.d.	n.d.	20
**22b** (**MA42**)	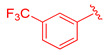	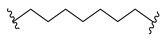	2S, 4R	n.d.	n.d.	30
**22c** (**MA78**)	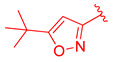	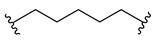	2S, 4R	89 ± 2	n.d.	80
**22d** (**MA77**)	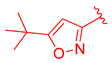	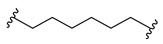	2S, 4R	89 ± 2	n.d.	80
**22e** (**MA73**)	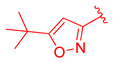	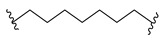	2S, 4R	84 ± 1	n.d.	80
**30a** (**MA190**)	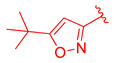	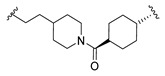	2S, 4R	71 ± 1	90 ± 2	95
**30b** (**MA191**)	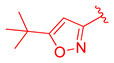	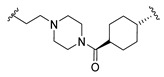	2S, 4R	88 ± 2	89 ± 2	95
**31a** (**MA224**) (neg. control)	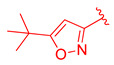	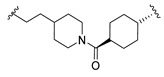	2R, 4S	n.d.	n.d.	No effect
**31b** (**MA225**) (neg. control)	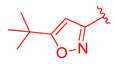	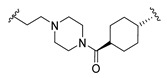	2R, 4S	97 ± 2	n.d.	No effect
**MA49** (Reference)	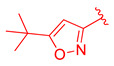	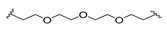	2S, 4R	91 ± 2	81 ± 0	80

* refers to the configuration of the chiral centers (R or S).

**Table 2:** Cytotoxicity in human HEK293 cells.

Cpd.	Cell Viability (%) at 50 μM
**MA68**	11.5 ± 2.2
**MA49**	2.9 ± 0.3
**MA190**	99.1 ± 3.1
**MA191**	86.9 ± 5.8

**Table 3:** Plasma (human) stability of PROTAC MA190, MA191 and the inhibitor MA68. Compounds were incubated for 6 h at 37 °C and quantified by HPLC.

Cpd. Id	0 min—Compound Remaining %	10 min—Compound Remaining %	30 min—Compound Remaining %	60 min—Compound Remaining %	120 min—Compound Remaining %	360 min—Compound Remaining %
**MA49**	100	94.9 ± 2.5	96.1 ± 1.7	95.2 ± 2.8	93.2 ± 6.7	95.4 ± 4.3
**MA68**	100	92.9 ± 0.2	94.2 ± 1.2	91.9 ± 3.1	93.6 ± 3.2	88.9 ± 3.2
**MA190**	100	94.1 ± 4.7	89.5 ± 1.8	88.3 ± 0.2	86.0 ± 2.0	86.2 ± 2.4
**MA191**	100	95.9 ± 9.4	93.3 ± 4.2	92.6 ± 5.7	94.9 ± 1.8	93.3 ± 0.7

**Table 4:** Microsomal stability of PROTACs and control diclofenac. Compounds were incubated for 2 h using mouse (CD-1) liver microsomes.

Cpd. Id	0 min—Compound Remaining %	10 min—Compound Remaining %	20 min—Compound Remaining %	30 min—Compound Remaining %	60 min—Compound Remaining %	120 min—Compound Remaining %
**MA49**	100	66.2 ± 2.3	52.9 ± 2.1	37.2 ± 0.6	28.8 ± 2.9	15.0 ± 2.5
**MA190**	100	91.5 ± 0.4	88.5 ± 1.2	93.9 ± 0.6	96.0 ± 0.2	92.0 ± 1.3
**MA191**	100	98.2 ± 0.9	97.0 ± 1.0	95.0 ± 1.2	91.2 ± 1.8	89.2 ± 1.0
**Diclofenac**	100	94.7 ± 1.7	86.9 ± 0.5	79.0 ± 1.3	70.1 ± 2.8	60.8 ± 2.2

## Data Availability

The original contributions presented in this study are included in the article/Supplementary Materials. Further inquiries can be directed to the corresponding authors.

## Supplementary Materials

The following supporting information can be downloaded at: https://www.mdpi.com/article/10.3390/ph19050756/s1, Figure S1: Docking results within the FLT3-MA49-VHL ternary model. (a) Our previously published FLT3-MA49-VHL ternary complex model. (b) Cartoon and surface view of FLT3 (orange) and VHL (purple) with docked PROTACs (green sticks). (C–H) Interactions of MA73 (c), MA74 (d), MA77 (e), and MA78 (f) within FLT3 and VHL binding pockets. Hydrogen bonds (≤2.5 Å) are shown as cyan dashed lines; Figure S2: Modeling of MAPK14-MA191-VHL ternary complex. (a) Interactions of docked MA68 with MAPK14 amino acid residues. (b) DP-1376 docked pose (magenta) superposed on its crystallographic pose (cyan, PDB ID 3NNU) within MAPK14 binding pocket. (c) Cartoon representation of the top-scored MAPK14-MA191-VHL ternary complex model generated by MOE Method 4B (d) Cartoon and surface view of FLT3 (orange) and VHL (purple) with docked PROTACs (green sticks). (e) and (f) Interactions of MA190 and MA191, respectively, within MAPK14 and VHL binding pockets. Hydrogen bonds (≤2.5 Å) are shown as cyan dashed lines; Table S1: Kinase selectivity profiling of MA68 and MA191 (Cellular Nano-Bret CELLinib128 assay). The residual binding of the respective kinase inhibitor (tracer) when using 1 μM MA68 or MA191 is shown (100% corresponds to no effect, 0% corresponds to complete displacement of the tracer); Table S2: HPLC Stability of PROTACs and inhibitor under cellular assay conditions; Table S3: Microsomal stability assay of MA49, MA190, MA191 and control diclofenac.

## Abbreviations

The following abbreviations are used in this manuscript:

AML	Acute myeloid leukemia
FLT3	FMS-like tyrosine kinase 3
ITD	Internal tandem duplications
TKD	Tyrosine kinase domain
FLT3i	FLT3 inhibitors
PROTACs	Proteolysis targeting chimeras
TPD	Targeted degradation of proteins
POI	Protein of interest
VHL	Von Hippel-Lindau
MAPK14	Mitogen-activated protein kinase 14
