# Supplementary material for: Dual FLT3/MAPK14 Proteolysis-Targeting Chimera (PROTAC) Induces Potent Acute Myeloid Leukemia Cell Death
Source: Pharmaceuticals (Basel). 2026 May 12;19(5):756. doi: 10.3390/ph19050756 (PMC13210772; doi:10.3390/ph19050756)
Supplement: Supplementary file 1 [file pharmaceuticals-19-00756-s001.zip › pharmaceuticals-4286121-supplementary.pdf]

Dual FLT3/MAPK14 Proteolysis-targeting Chimera (PROTAC) Induces Potent Acute Myeloid Leukemia Cell Death

Mohamed Abdelsalam [1,2,#], Melisa Halilovic [3,#], Ramy Ashry [3], Husam Nassar [1], Frank Erdmann [1], Matthias Schmidt[1], Oliver H. Krämer [3,+,*], and Wolfgang Sippl [1,+,*]

[#] these authors contributed equally to this work

[+] equal last author contribution

[*] Correspondence: wolfgang.sippl@pharmazie.uni-halle.de (chemistry) or okraemer@uni-mainz.de (biology)

[1] Institute of Pharmacy, Martin-Luther University of Halle-Wittenberg, 06120 Halle/Saale (Germany)

[2] Department of Pharmaceutical Chemistry, Faculty of Pharmacy, Alexandria University, Alexandria 21521 (Egypt)

[3] Institute of Toxicology, University Medical Center, Johannes Gutenberg-University Mainz, 55131, Mainz, (Germany)

Table of Contents

Content	Page No.
**Supplementary Figures**	**2**
**Supplementary Tables**	**4**
**Charts of analytical and spectral characterization of PROTACs**	**8**
**HPLC chromatrograms microsomal stability assay**	**28**

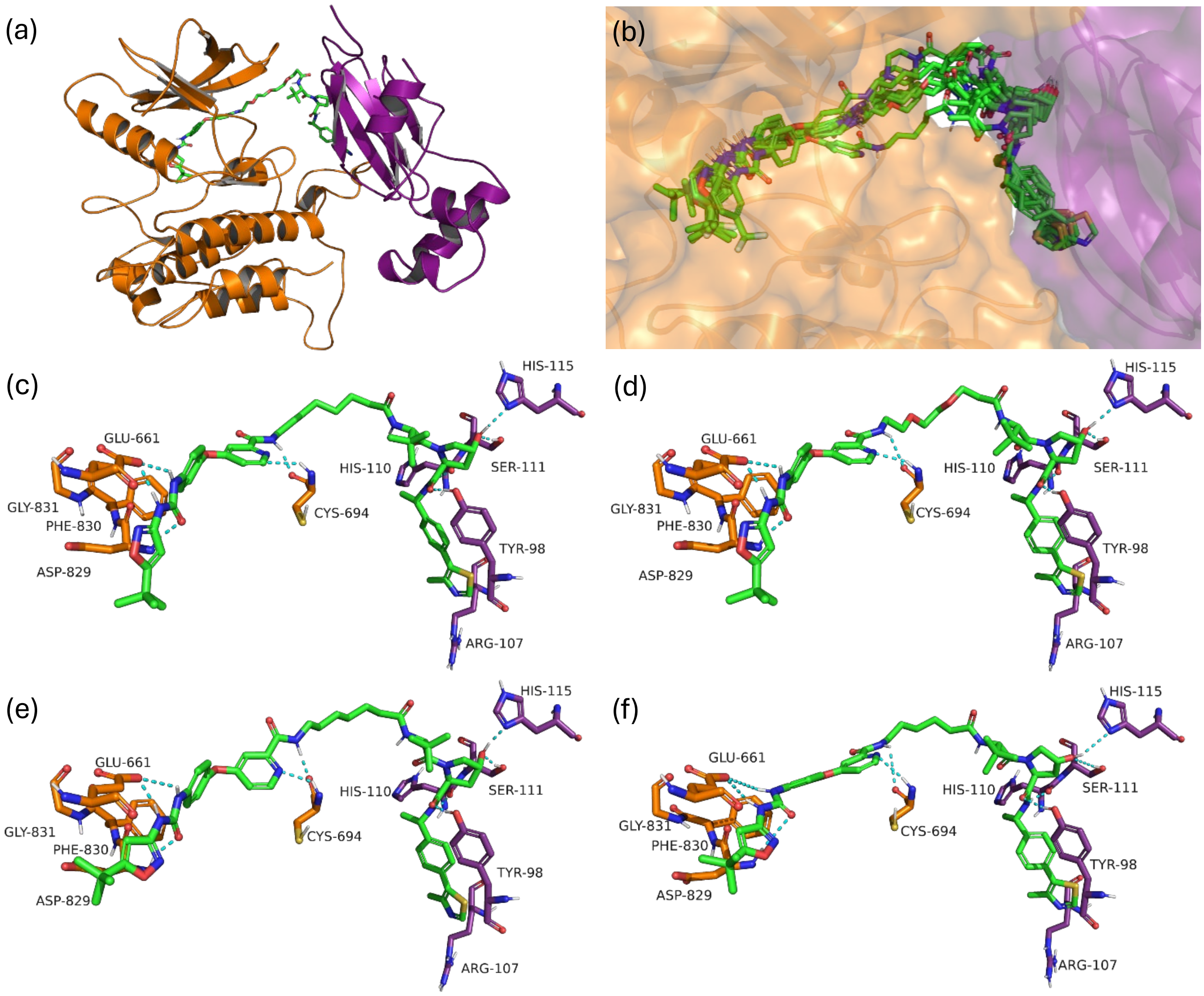

Figure S1: Docking results within the FLT3-MA49-VHL ternary model. (a) Our previously published FLT3-MA49-VHL ternary complex model. (b) Cartoon and surface view of FLT3 (orange) and VHL (purple) with docked PROTACs (green sticks). (C-H) Interactions of MA73 (c), MA74 (d), MA77 (e), and MA78 (f) within FLT3 and VHL binding pockets. Hydrogen bonds (≤ 2.5 Å) are shown as cyan dashed lines.

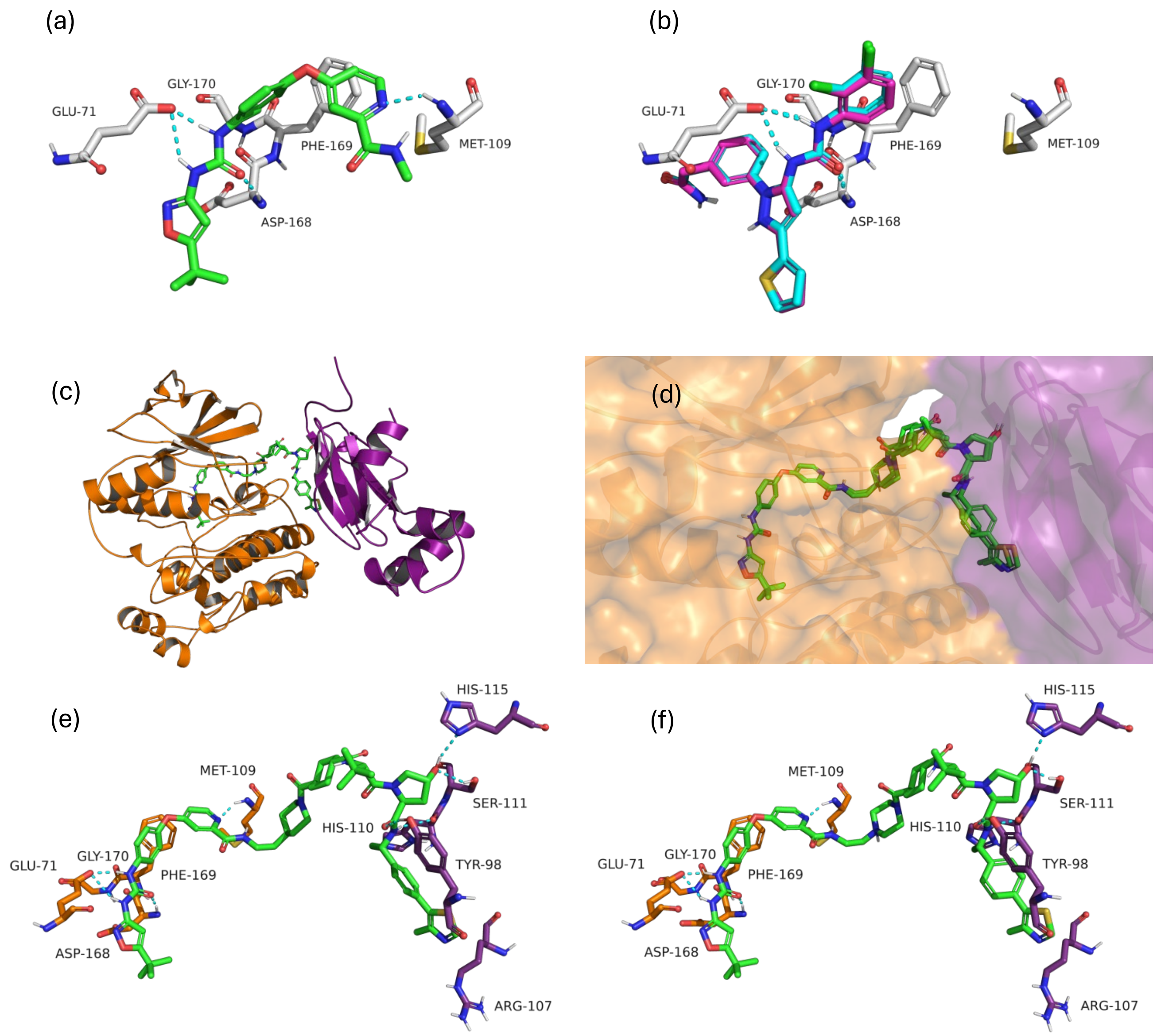

Figure S2: Modeling of MAPK14-MA191-VHL ternary complex. (a) Interactions of docked MA68 with MAPK14 amino acid residues. (b) DP-1376 docked pose (magenta) superposed on its crystallographic pose (cyan, PDB ID 3NNU) within MAPK14 binding pocket. (c) Cartoon representation of the top-scored MAPK14-MA191-VHL ternary complex model generated by MOE Method 4B (d) Cartoon and surface view of FLT3 (orange) and VHL (purple) with docked PROTACs (green sticks). (e) and (f) Interactions of MA190 and MA191, respectively, within MAPK14 and VHL binding pockets. Hydrogen bonds (≤ 2.5 Å) are shown as cyan dashed lines.

Supplementary Tables

Table S1. Kinase selectivity profiling of MA68 and MA191 (Cellular Nano-Bret CELLinib128 assay). The residual binding of the respective kinase inhibitor (tracer) when using 1µM MA68 or MA191 is shown (100% corresponds to no effect, 0% corresponds to complete displacement of the tracer).

Screening Concentration. [M]	1.00E-06	1.00E-06		
**Kinases**	**MA68**	**MA191**	**Ctrl Cpd**	**Ctrl Cpd EC_{50}. [M]**
**ABL1**	71.8	104.8	Dasatinib	1.21E-08
**ALK**	109.4	105.3	Staurosporine	8.60E-07
**AURKA**	120.0	120.0	CC1	1.54E-08
**AURKB**	76.4	109.4	CC1	1.69E-08
**AURKC**	102.0	110.2	CC1	2.28E-08
**BRSK1**	105.8	107.4	CC1	5.99E-08
**BTK**	120.0	120.0	CC1	6.20E-08
**CAMK1**	100.4	93.6	Staurosporine	1.63E-07
**CAMK1D**	101.7	85.4	Staurosporine	4.90E-08
**CAMK1G**	101.1	91.6	Staurosporine	1.20E-07
**CAMK2A**	101.6	102.5	CC1	1.59E-07
**CDK14-CCNY**	42.0	95.7	CC1	7.47E-08
**CDK17-CCNY**	13.9	81.3	CC1	1.13E-08
**CDK18-CCNY**	28.6	95.9	CC1	3.08E-08
**CDK1-CCNB1**	98.0	81.9	Staurosporine	1.23E-07
**CDK20-CCNH**	93.3	108.0	CC1	2.35E-07
**CDK2-CCNE1**	96.0	102.5	CC1	6.79E-08
**CDK3-CCNE1**	81.0	117.0	CC1	4.90E-08
**CDK4-CCND3**	29.7	104.6	CC1	1.24E-08
**CDK5-CDK5R2**	86.1	104.0	CC1	6.07E-08
**CHEK2**	119.3	111.1	CC1	2.56E-07
**CLK4**	93.8	100.3	Staurosporine	3.76E-08
**CSF1R**	27.1	107.6	Dasatinib	1.18E-08
**CSNK1D**	103.7	120.0	Staurosporine	1.04E-05
**CSNK1G2**	95.1	92.4	CC1	2.43E-07
**CSNK2A2**	109.5	113.9	CC1	2.97E-08
**DCLK3**	113.0	102.3	CC1	1.46E-07
**DDR1**	4.0	85.7	Dasatinib	2.59E-09
**EPHA1**	37.2	120.0	Dasatinib	1.43E-08
**EPHA2**	10.6	104.0	Dasatinib	6.86E-09
**EPHA4**	33.7	118.1	Dasatinib	2.86E-09
**EPHA5**	41.1	120.0	Dasatinib	3.39E-09
**EPHA6**	23.6	82.5	CC1	1.98E-07
**EPHA8**	38.9	120.0	Dasatinib	2.33E-09
**EPHB1**	27.7	85.1	Staurosporine	2.86E-06
**EPHB2**	49.5	120.0	Dasatinib	3.35E-09

**EPHB3**	44.8	120.0	Dasatinib	1.72E-08
**EPHB4**	3.8	120.0	Dasatinib	2.32E-09
**EPHB6**	99.5	120.0	Dasatinib	5.83E-08
**ERN1**	116.7	116.3	CC1	2.47E-08
**ERN2**	120.0	116.3	CC1	1.11E-07
**FGFR1**	69.2	103.1	CC1	2.71E-08
**FGFR2**	40.7	108.6	CC1	2.87E-08
**FGFR4**	86.5	92.7	CC1	1.28E-07
**FGR**	118.8	120.0	Dasatinib	4.10E-08
**FRK**	68.2	116.7	Dasatinib	1.37E-08
**FYN**	113.7	120.0	Dasatinib	1.27E-08
**GSK3A**	95.9	118.4	Staurosporine	7.26E-08
**GSK3B**	120.0	120.0	Staurosporine	1.22E-07
**HCK**	120.0	120.0	Dasatinib	2.25E-08
**HUNK**	88.2	102.6	CC1	8.77E-07
**IGF1R**	111.3	106.8	Staurosporine	3.30E-06
**IRAK1**	108.5	102.5	Staurosporine	1.29E-05
**IRAK3**	120.0	115.0	CC1	1.53E-08
**IRAK4**	94.3	114.7	CC1	9.11E-09
**JAK2**	110.6	105.7	CC1	1.63E-07
**JAK3**	110.8	116.6	CC1	9.25E-08
**LATS1**	105.7	97.8	CC1	1.89E-07
**LIMK2**	88.5	105.1	CC1	5.89E-08
**LTK**	102.2	101.4	Staurosporine	3.80E-07
**MAP3K11**	109.2	108.2	CC1	2.82E-07
**MAP3K9**	111.4	108.4	CC1	1.04E-07
**MAP4K1**	101.2	105.4	CC1	1.11E-07
**MAP4K2**	106.6	107.2	CC1	3.91E-08
**MAP4K3**	102.6	91.1	CC1	1.38E-07
**MAP4K5**	96.3	94.0	CC1	2.98E-07
**MAPK11**	24.0	96.2	Dasatinib	8.62E-07
**MAPK14**	25.9	70.3	Dasatinib	1.66E-07
**MAPK6**	112.7	120.0	CC1	4.34E-09
**MAPK9**	94.9	101.6	CC1	1.67E-07
**MARK2**	112.5	110.5	Staurosporine	5.17E-09
**MARK3**	105.1	102.0	Staurosporine	9.35E-09
**MLTK**	15.3	54.8	CC1	5.79E-08
**MUSK**	6.0	66.0	CC1	3.91E-08
**MYLK3**	118.9	107.5	CC1	8.08E-08
**NEK1**	109.9	95.9	CC1	2.31E-07
**NEK3**	120.0	100.5	CC1	2.11E-07
**NEK9**	108.7	109.0	CC1	1.81E-07
**NUAK1**	120.0	120.0	CC1	2.35E-08
**PAK4**	97.7	100.7	CC1	4.13E-07
**PAK6**	115.6	120.0	CC1	3.20E-07
**PHKG1**	87.3	93.5	CC1	2.13E-08
**PLK2**	98.1	107.6	Staurosporine	4.71E-06
**PLK4**	106.7	111.8	CC1	1.74E-07

**PRKAA1**	113.7	110.2	CC1	3.41E-08
**PRKAA2**	108.6	118.9	CC1	2.85E-08
**PRKACB**	99.6	105.6	CC1	5.90E-08
**PRKCD-PMA**	112.8	114.9	CC1	2.14E-07
**PRKCE-PMA**	76.2	78.3	CC1	3.18E-07
**PRKCQ-PMA**	107.8	104.1	CC1	1.58E-07
**PTK2**	117.7	112.8	CC1	6.39E-08
**PTK6**	67.6	117.9	Dasatinib	1.84E-08
**RET**	3.8	83.5	CC1	3.83E-08
**RIOK2**	108.8	114.6	CC1	7.35E-07
**RIPK1**	93.3	103.6	Staurosporine	4.76E-06
**RON**	100.4	98.5	Staurosporine	5.97E-06
**RPS6KA1**	108.3	110.0	CC1	3.78E-08
**RPS6KA2**	116.4	114.1	CC1	1.11E-07
**RPS6KA3**	118.5	120.0	CC1	2.90E-08
**RPS6KA6**	113.5	107.5	CC1	5.43E-08
**SIK1**	110.5	115.0	CC1	3.20E-08
**SIK3**	113.2	106.3	CC1	7.95E-08
**SNRK**	99.2	97.0	CC1	1.44E-07
**SRC**	120.0	120.0	Dasatinib	2.14E-08
**SRMS**	94.4	94.4	CC1	6.98E-07
**STK17B**	102.2	92.4	Staurosporine	1.20E-07
**STK26**	88.5	82.3	Staurosporine	5.18E-07
**STK3**	99.3	105.1	CC1	8.87E-08
**STK32B**	104.2	92.5	Staurosporine	1.71E-07
**STK33**	94.8	99.0	CC1	5.38E-08
**STK35**	59.6	95.8	CC1	4.36E-08
**STK36**	108.1	118.6	CC1	2.76E-08
**STK38L**	90.0	90.0	CC1	5.25E-07
**STK4**	98.8	102.0	CC1	3.83E-08
**TBK1**	106.9	108.5	CC1	9.56E-08
**TEC**	107.2	112.9	Dasatinib	9.58E-08
**TEK**	12.6	85.7	CC1	4.96E-08
**TESK1**	102.4	116.5	Dasatinib	3.22E-07
**TNK1**	113.8	120.0	CC1	5.41E-08
**TNK2**	115.7	118.2	CC1	2.09E-07
**TNNI3K**	15.1	83.2	CC1	1.64E-07
**TRKA**	28.6	102.8	CC1	1.52E-07
**TYK2**	103.2	105.6	CC1	1.49E-07
**ULK1**	117.4	111.5	CC1	3.01E-08
**ULK2**	91.7	98.6	CC1	2.36E-08
**ULK3v1**	96.1	107.8	CC1	8.66E-08
**WEE2**	102.1	99.3	CC1	9.57E-08
**YES1**	77.1	120.0	Dasatinib	1.11E-08
**Target Count < 50 % tracer bound**	**25**	**0**		

Table S2. HPLC Stability of PROTACs and inhibitor under cellular assay conditions

Cpd.	0 h - %	6 h- %	12 h- %	24 h- %	48 h- %	72 h- %
**MA49**	100	101.1	101.4	103.5	104.2	104.9
**MA42**	100	99.7	100.0	100.2	100.8	101.0
**MA43**	100	100.1	100.3	100.5	101.0	101.2
**MA73**	100	101.2	101.3	102.2	103.3	106.1
**MA74**	100	101.4	101.4	101.8	102.6	103.1
**MA77**	100	99.6	99.6	99.9	100.5	100.7
**MA78**	100	99.5	99.5	99.9	100.6	101.0
**MA190**	100	102.6	103.2	104.4	105.4	107.4
**MA191**	100	100.3	100.0	99.7	100.3	100.3
**MA68**	100	101.0	102.4	102.1	105.7	105.2

^{1}HNMR of 19 (MA74)

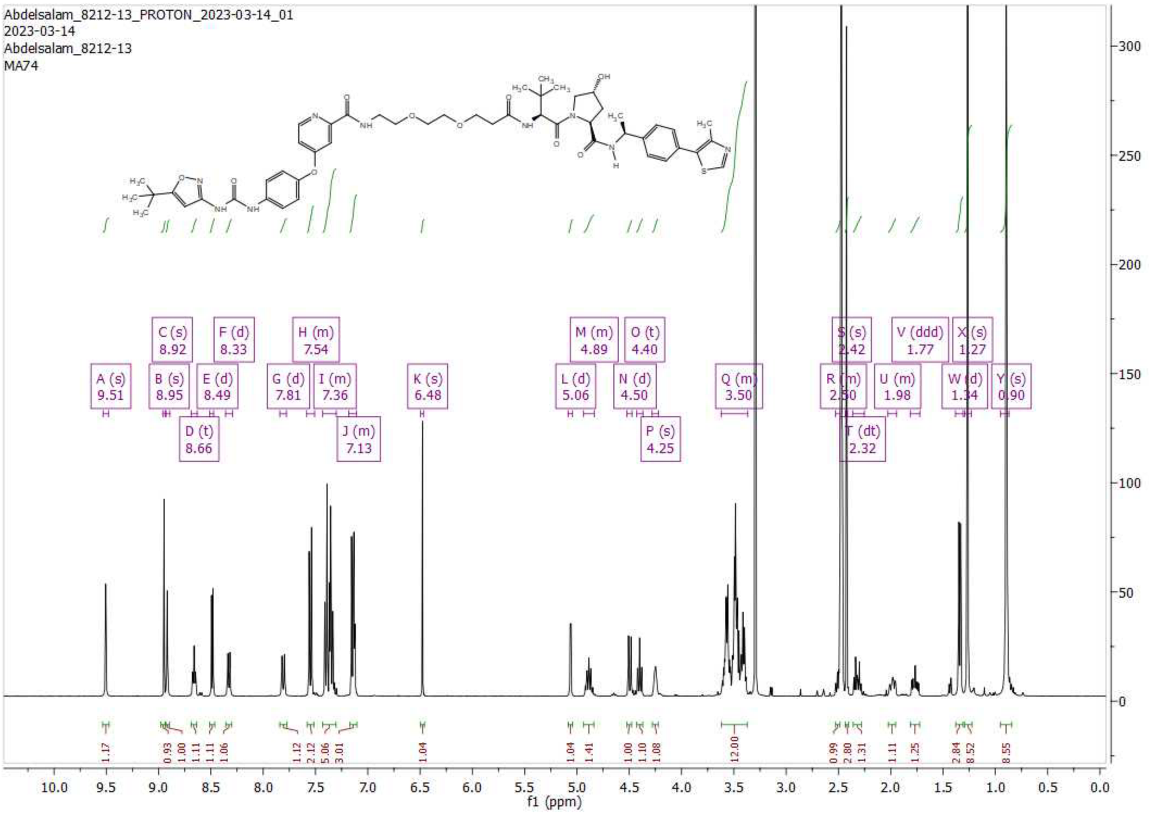

^{13}CNMR of 19 (MA74)

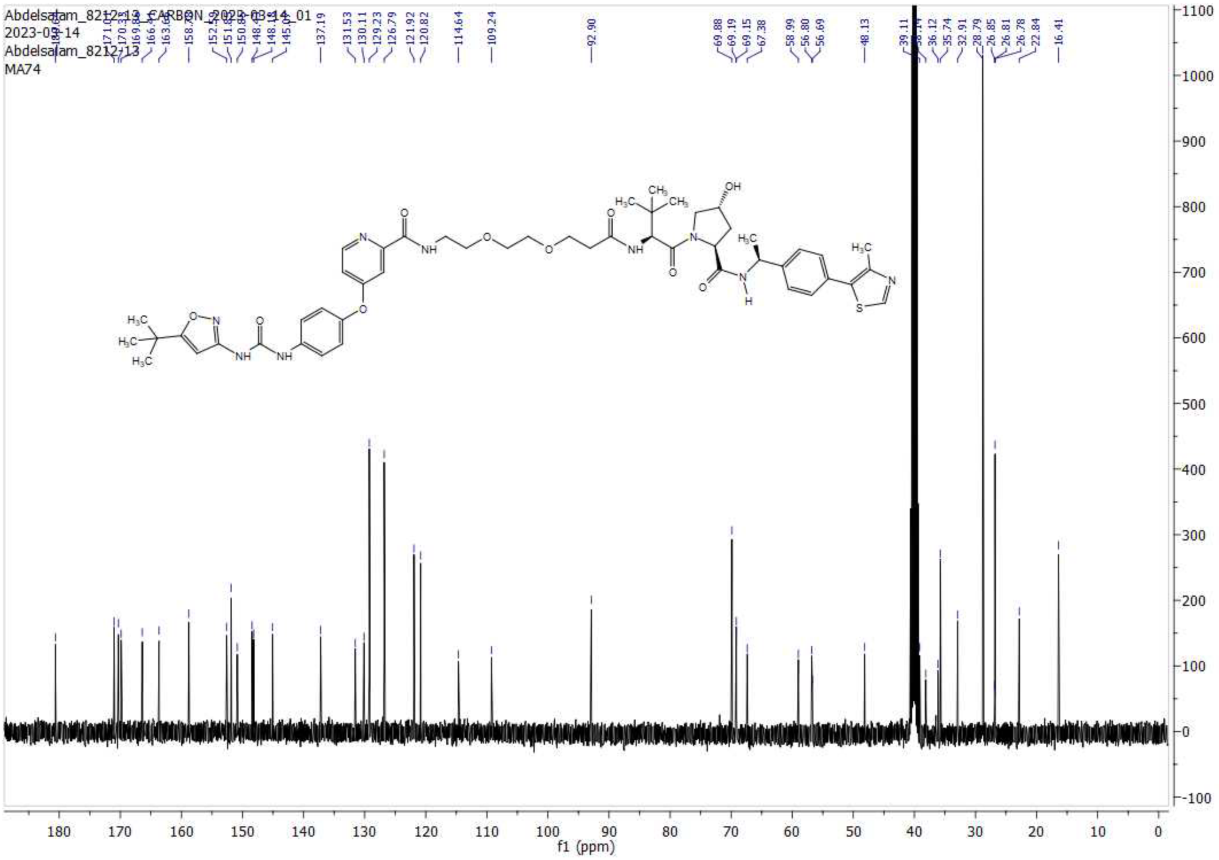

HRMS of 19 (MA74)

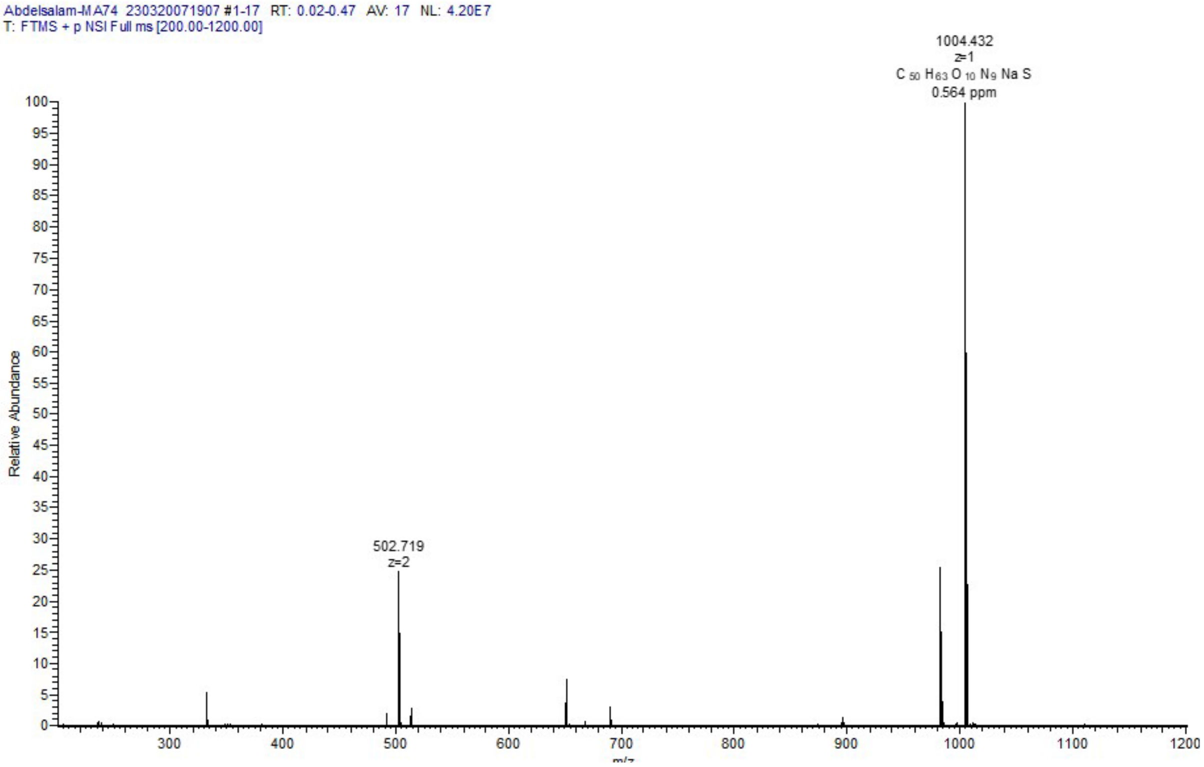

HPLC of 19 (MA74)

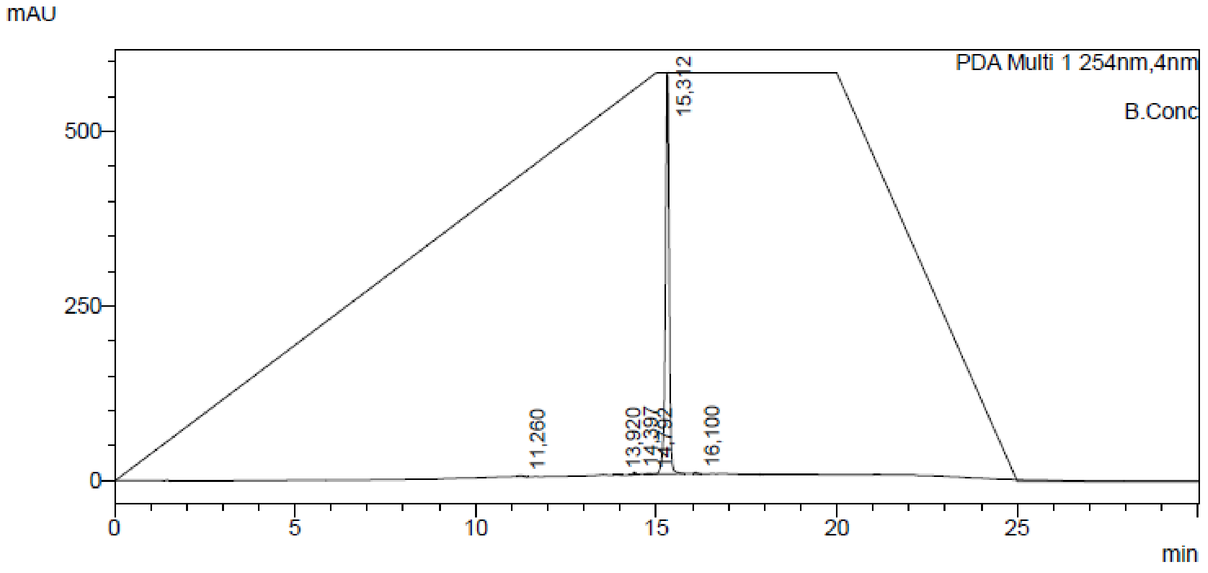

PDA Ch1 254nm

Peak#	Ret. Time	Area	Height	Area%
1	11,260	10237	1275	0,248
2	13,920	7656	1130	0,186
3	14,397	17884	3276	0,434
4	14,792	5173	770	0,125
5	15,312	4071162	574831	98,736
6	16,100	11170	1901	0,271
Total		4123282	583184	100,000

^{1}HNMR of 22a (MA43)

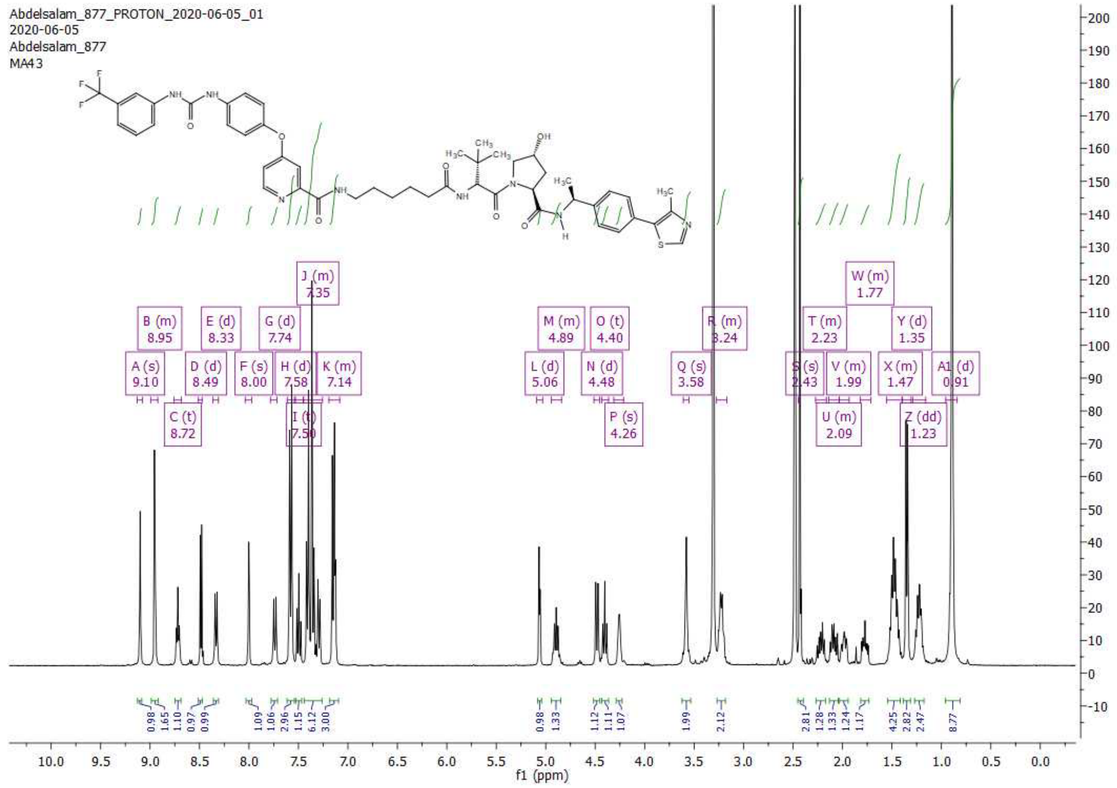

^{13}CNMR of 22a (MA43)

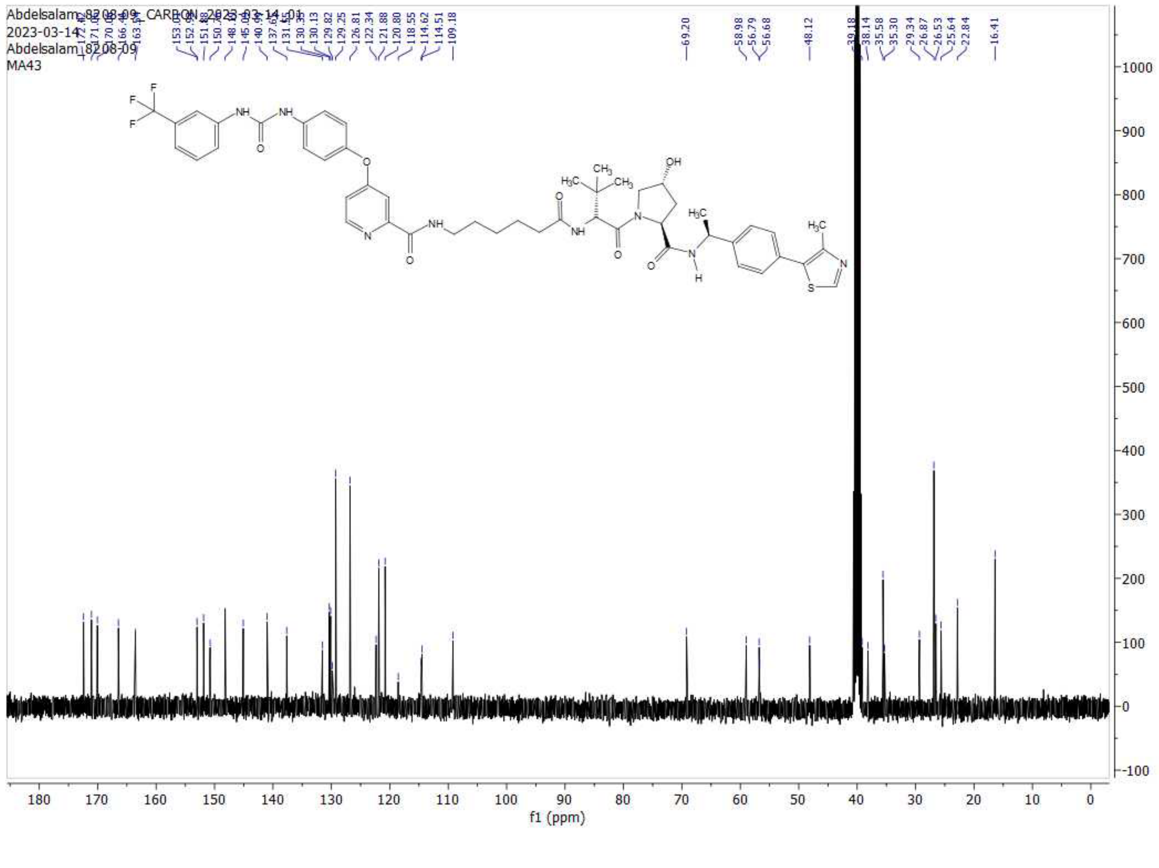

HRMS of 22a (MA43)

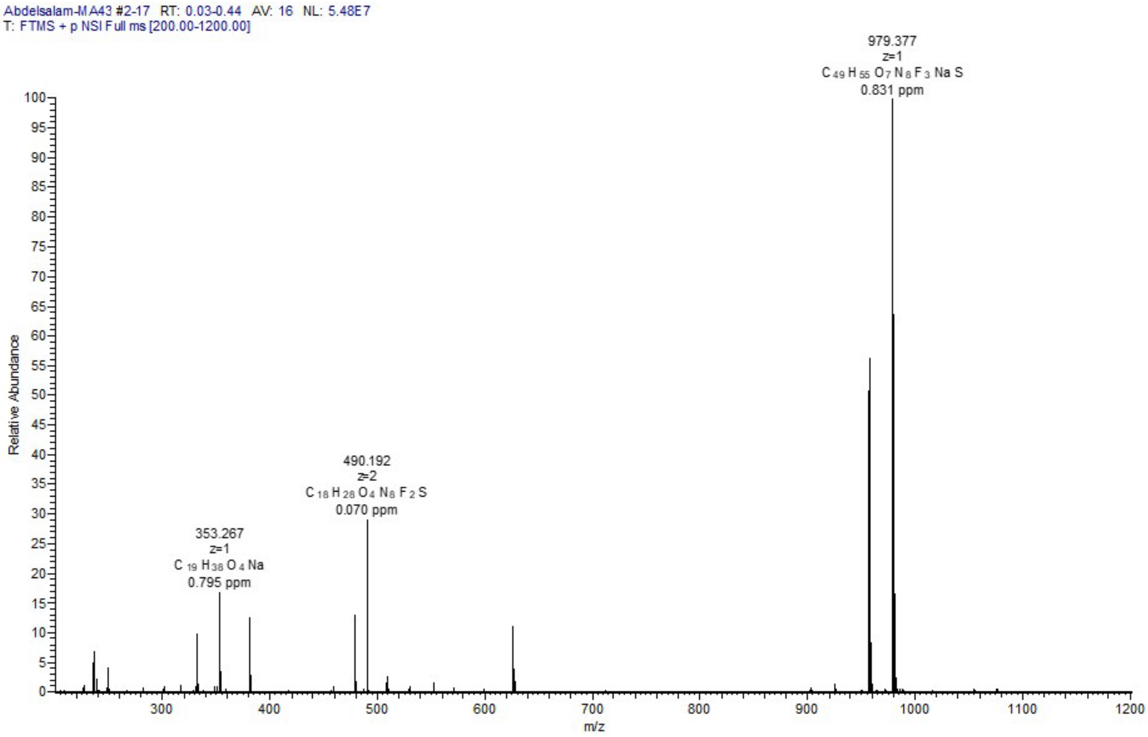

HPLC of 22a (MA43)

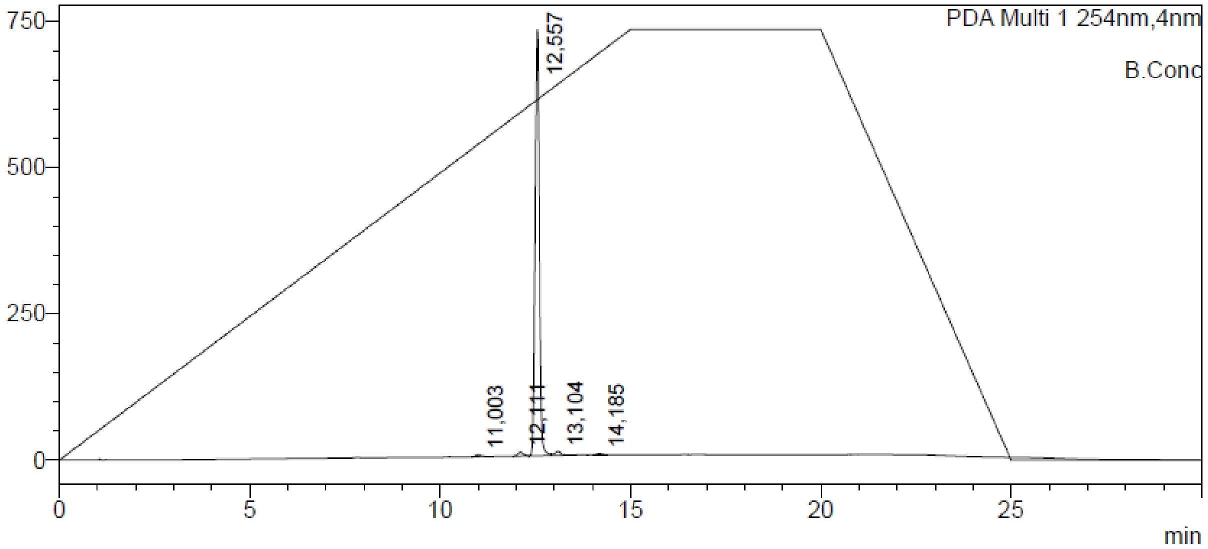

PDA Ch1 254nm

Peak#	Ret. Time	Area	Height	Area%
1	11,003	22650	2834	0,381
2	12,111	56897	5967	0,958
3	12,557	5770555	729186	97,163
4	13,104	67780	6417	1,141
5	14,185	21167	2039	0,356
Total		5939048	746442	100,000

^{1}HNMR of 22b (MA42)

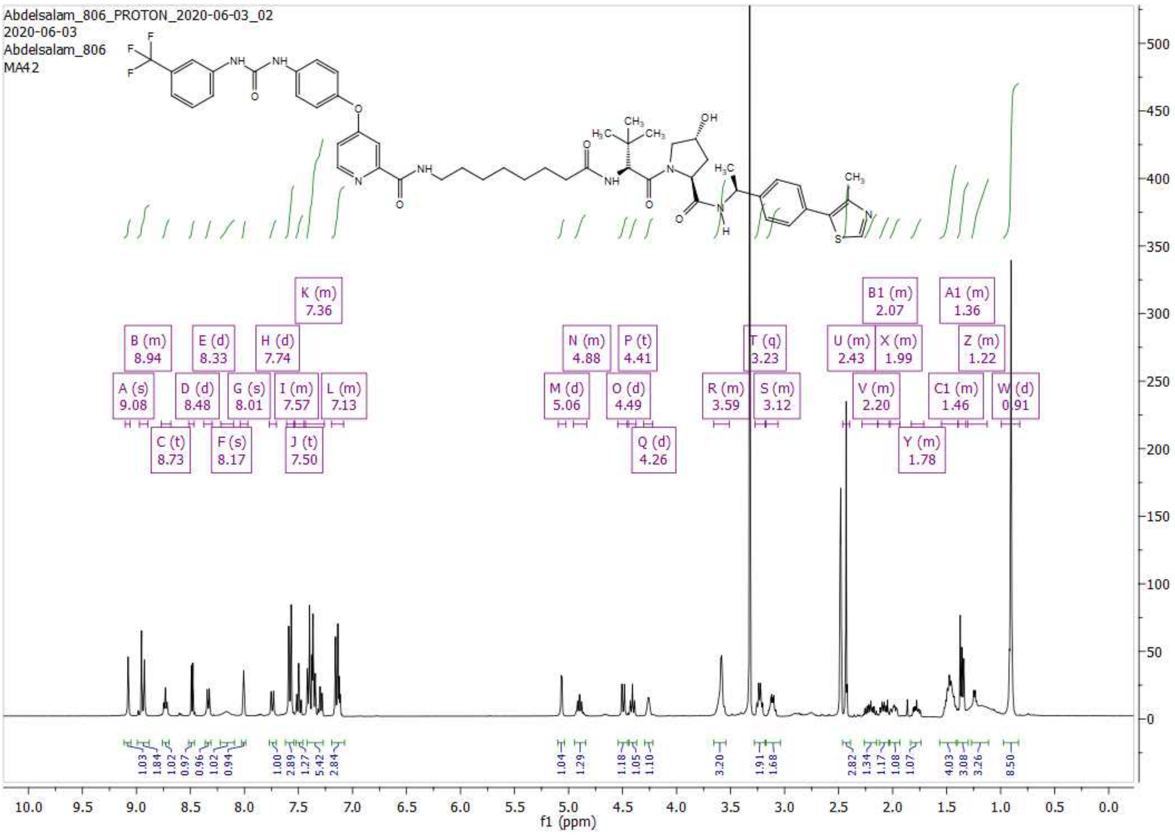

^{13}C NMR of 22b (MA42)

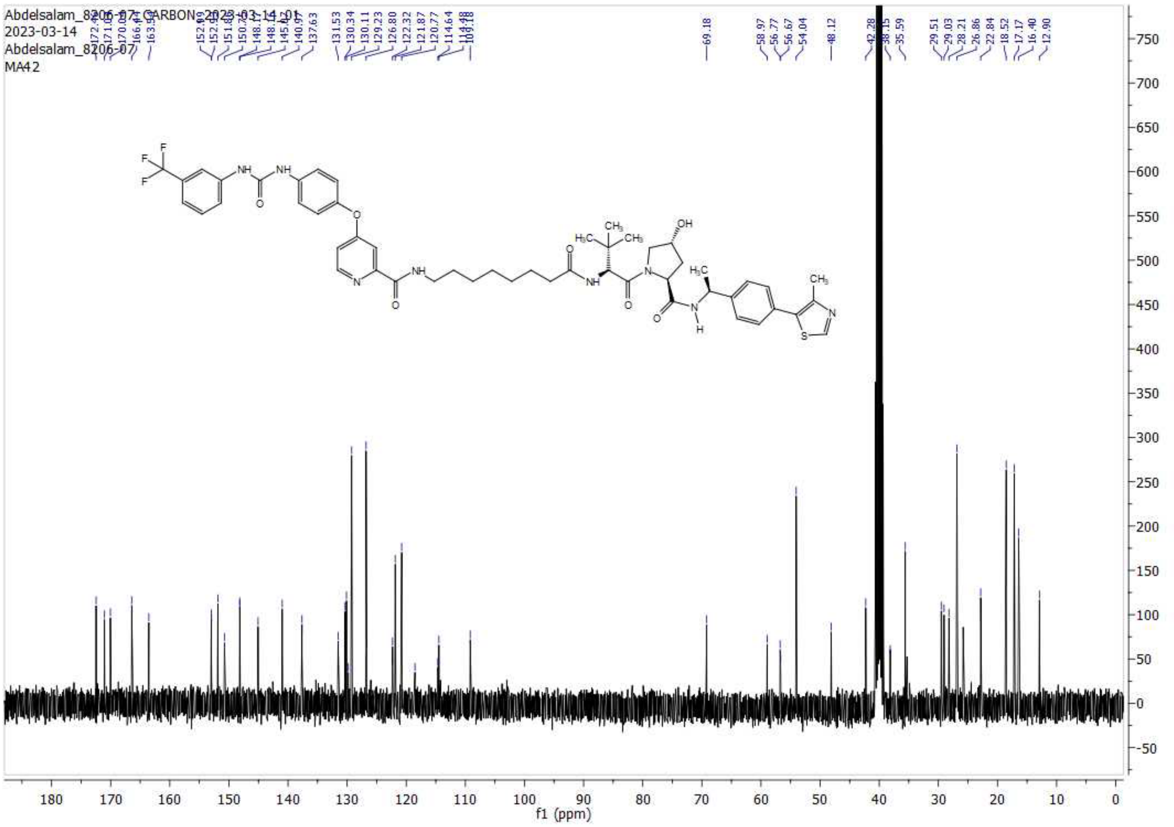

HRMS of 22b (MA42)

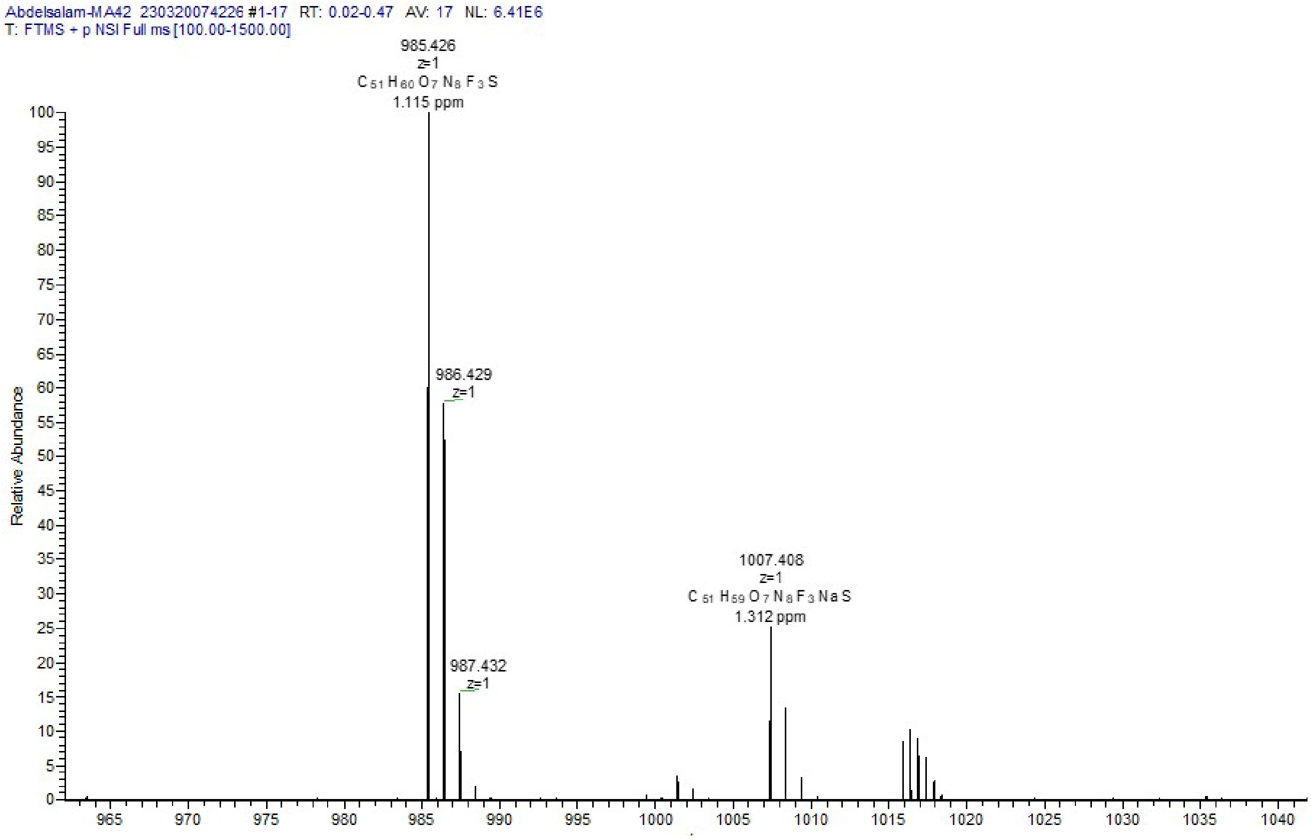

HPLC of 22b (MA42)

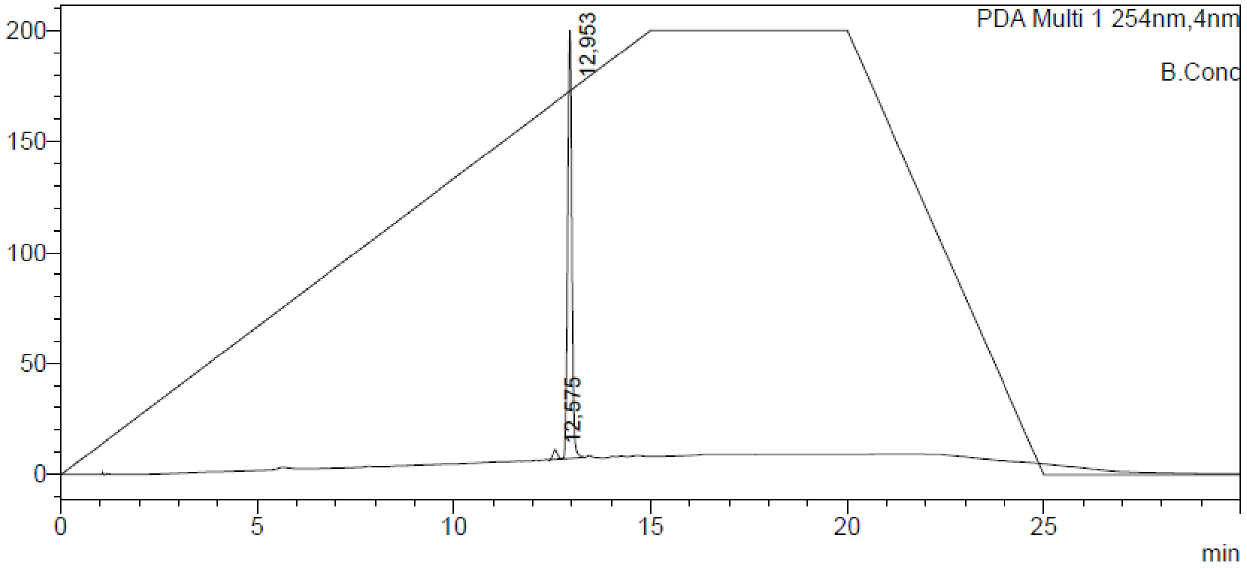

PDA Ch1 254nm

Peak#	Ret. Time	Area	Height	Area%
1	12,575	38319	4570	2,628
2	12,953	1419543	192903	97,372
Total		1457862	197473	100,000

[1] HNMR of 22c (MA78)

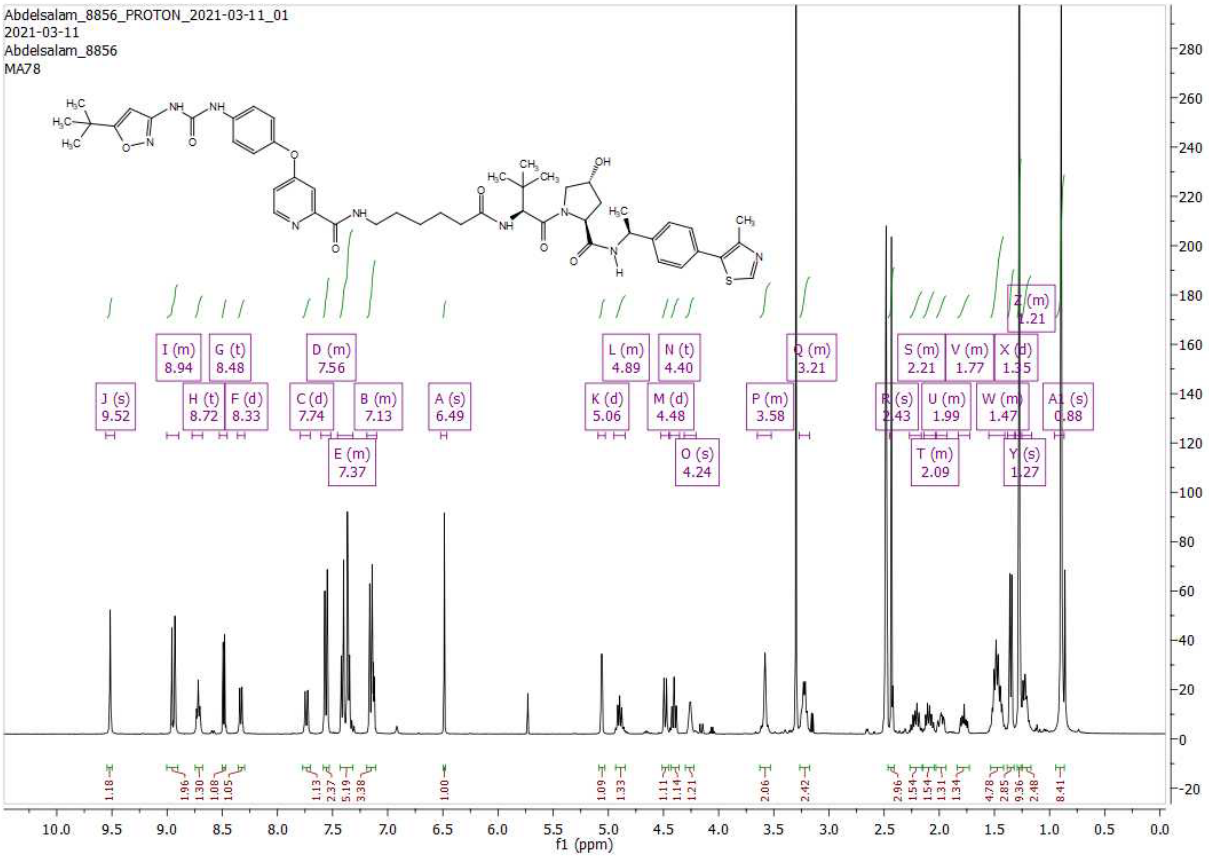

[13] CNMR of 22c (MA78)

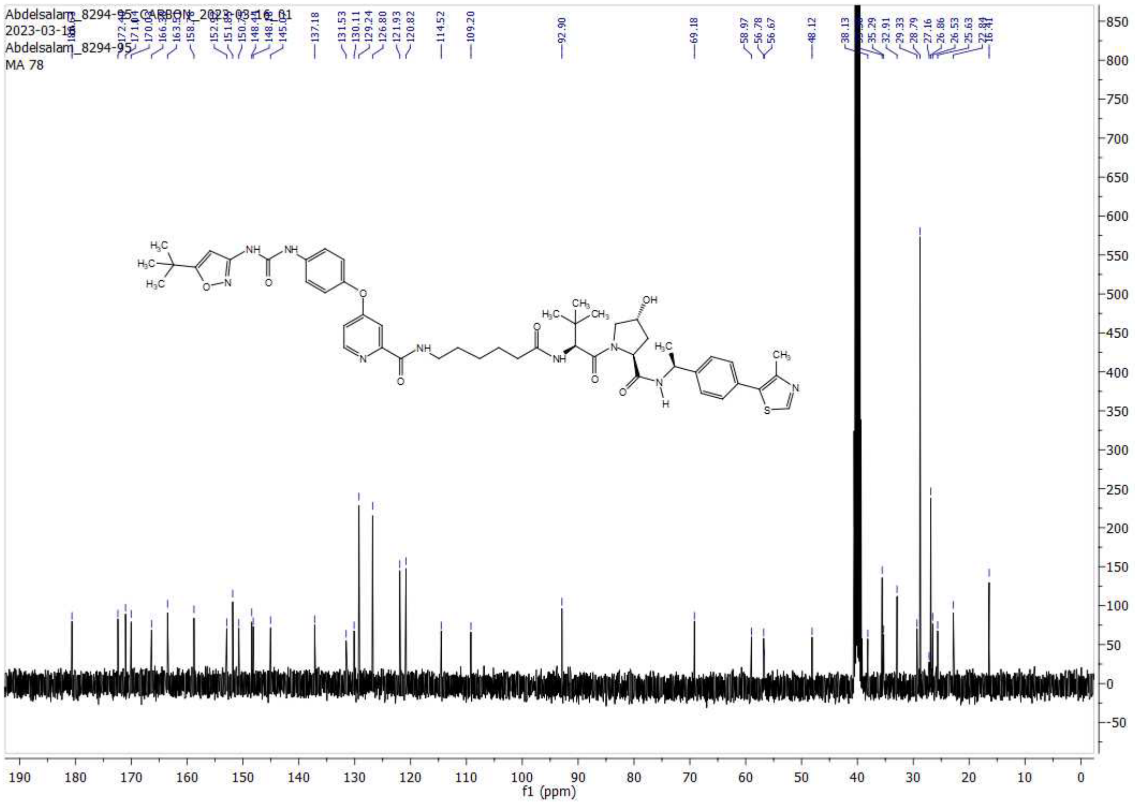

HRMS of 22c (MA78)

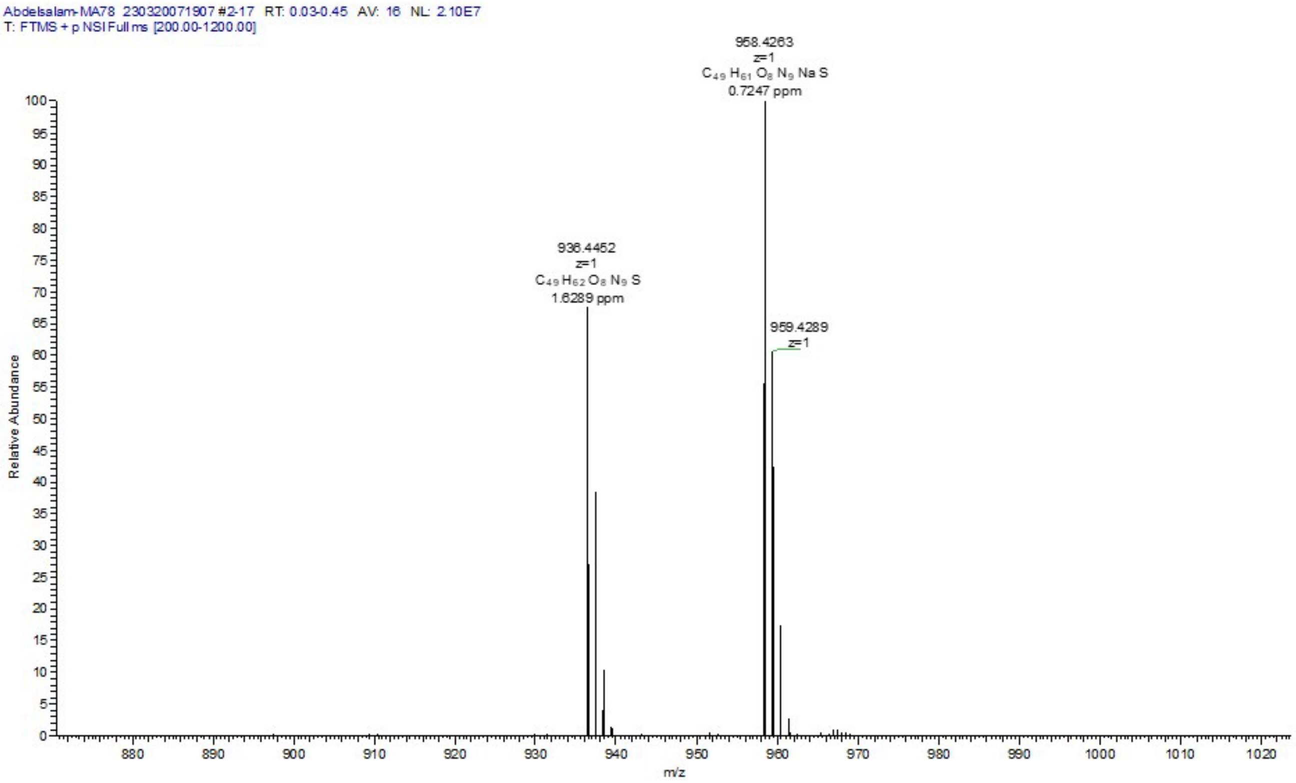

HPLC of 22c (MA78)

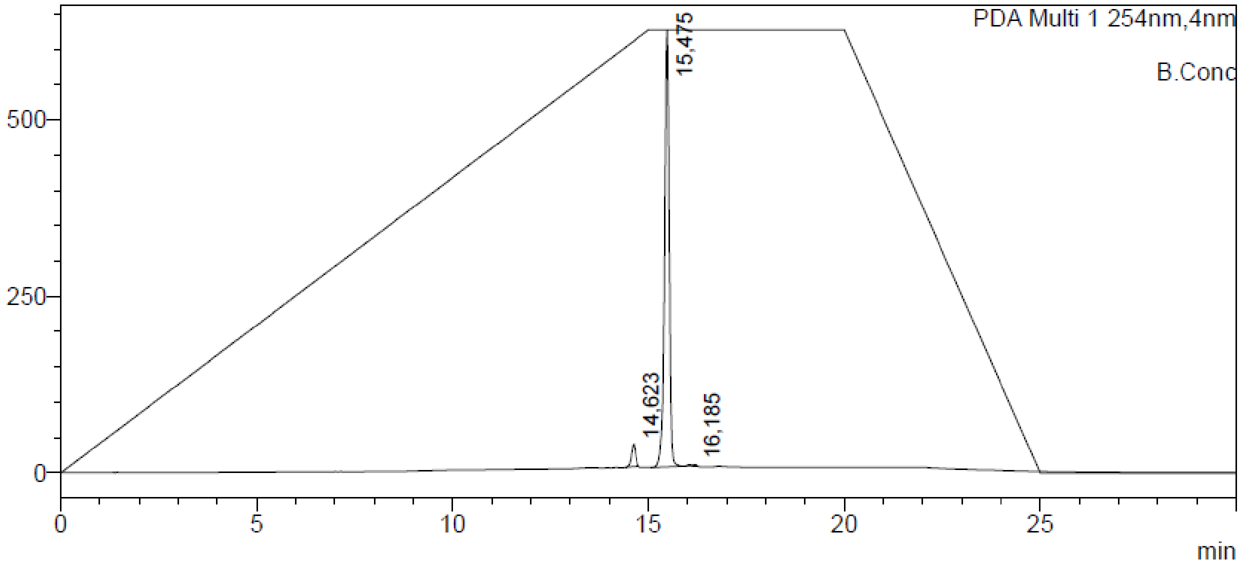

PDA Ch1 254nm

Peak#	Ret. Time	Area	Height	Area%
1	14,623	233735	31936	4,396
2	15,475	5058257	618881	95,129
3	16,185	25295	2215	0,476
Total		5317288	653031	100,000

^{1}HNMR of 22d (MA77)

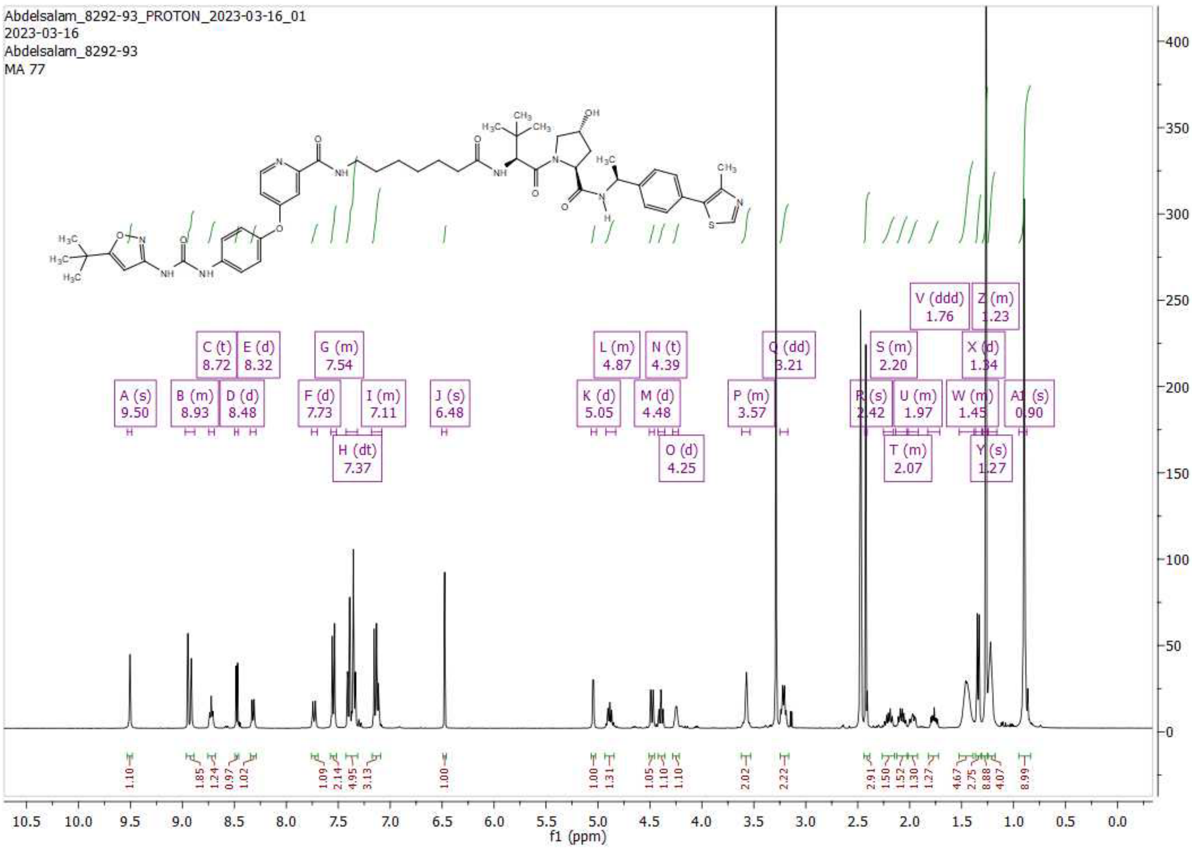

^{13}CNMR of 22d (MA77)

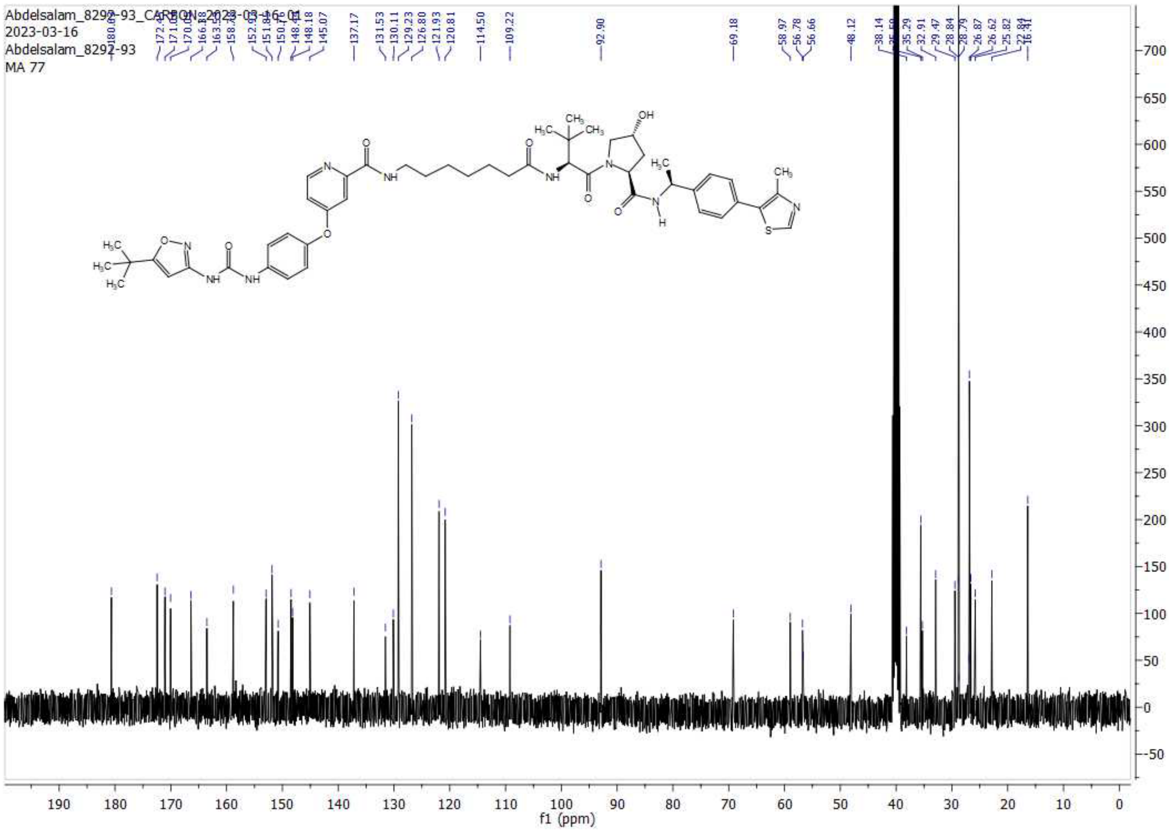

HRMS of 22d (MA77)

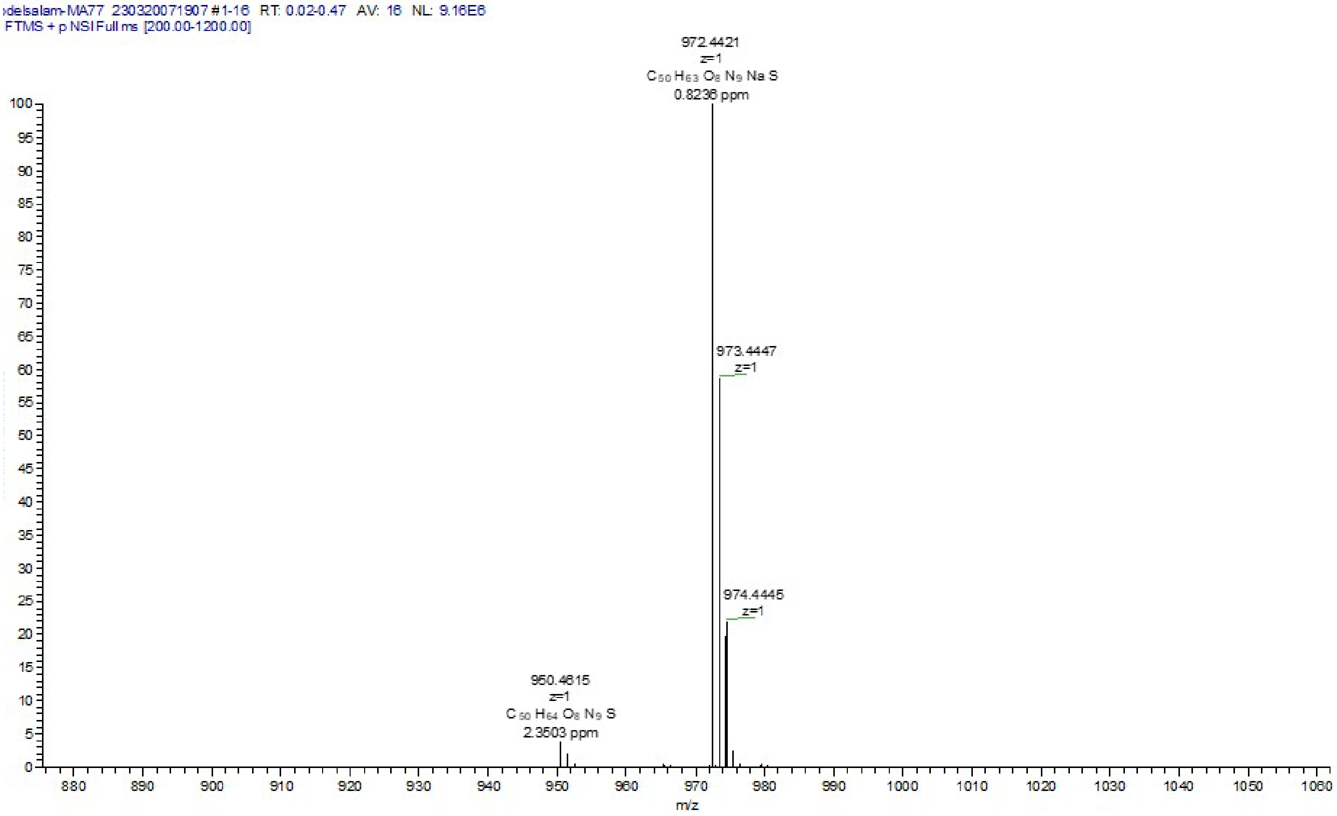

HPLC of 22d (MA77)

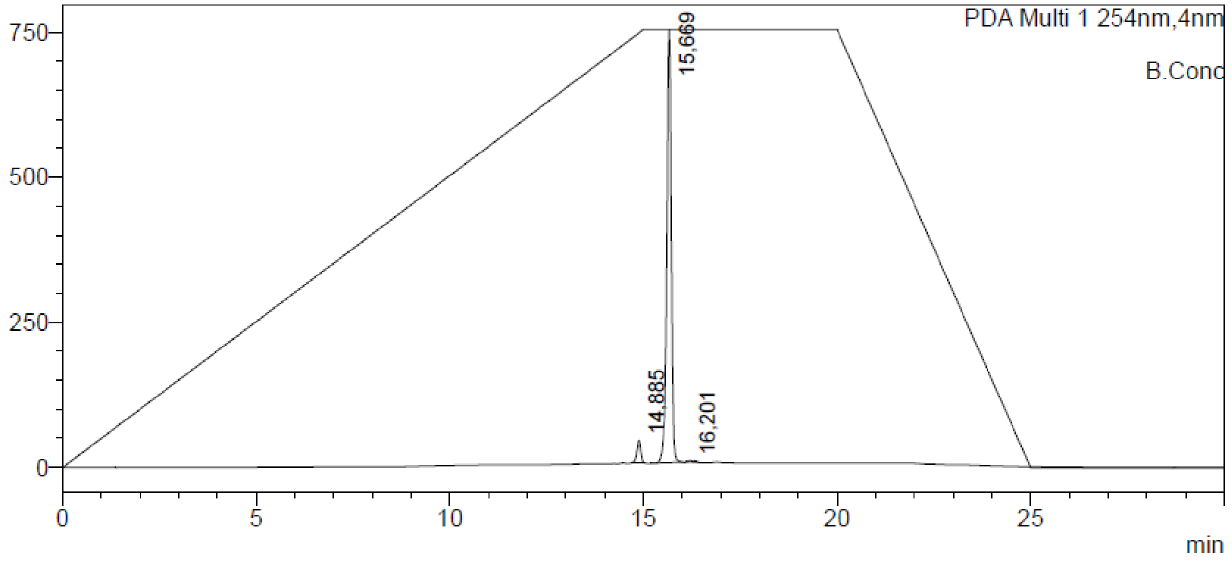

PDA Ch1 254nm

Peak#	Ret. Time	Area	Height	Area%
1	14,885	284526	38907	4,483
2	15,669	6031492	746176	95,033
3	16,201	30709	2486	0,484
Total		6346726	787569	100,000

^{1}HNMR of 22e (MA73)

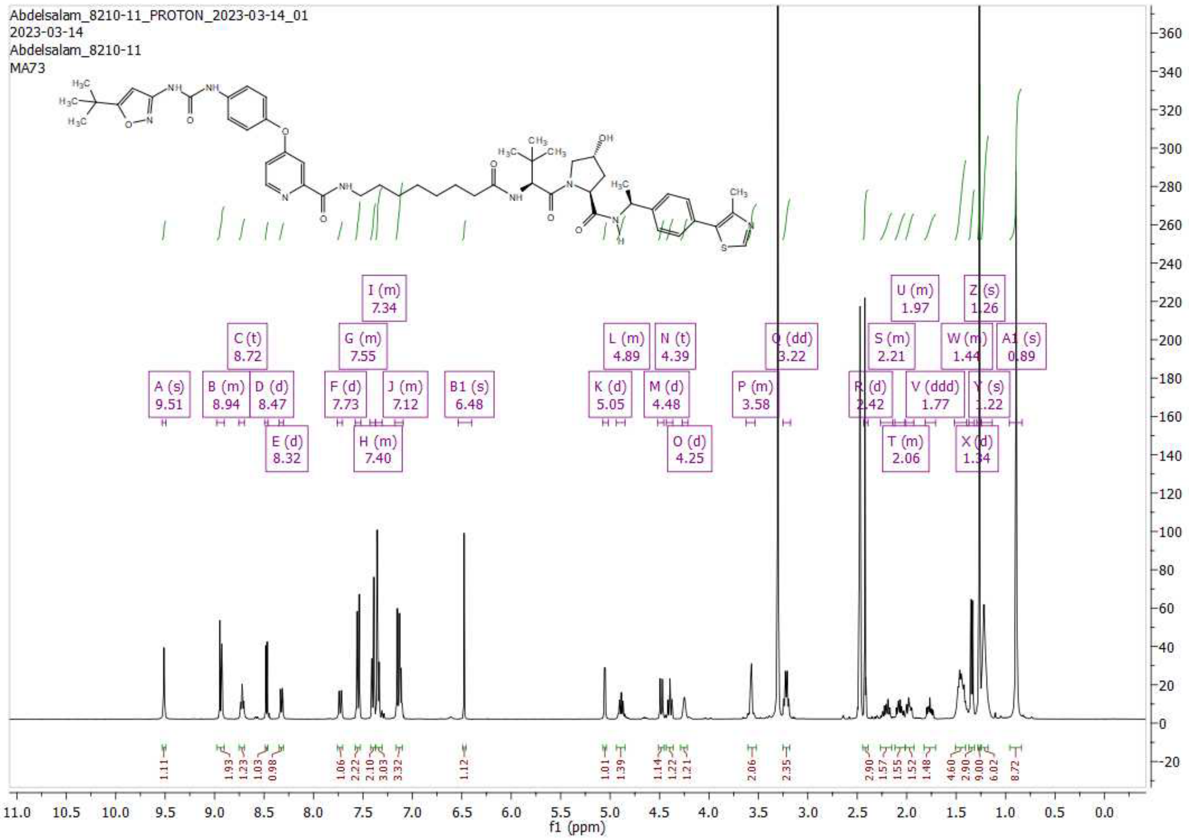

^{13}CNMR of 22e (MA73)

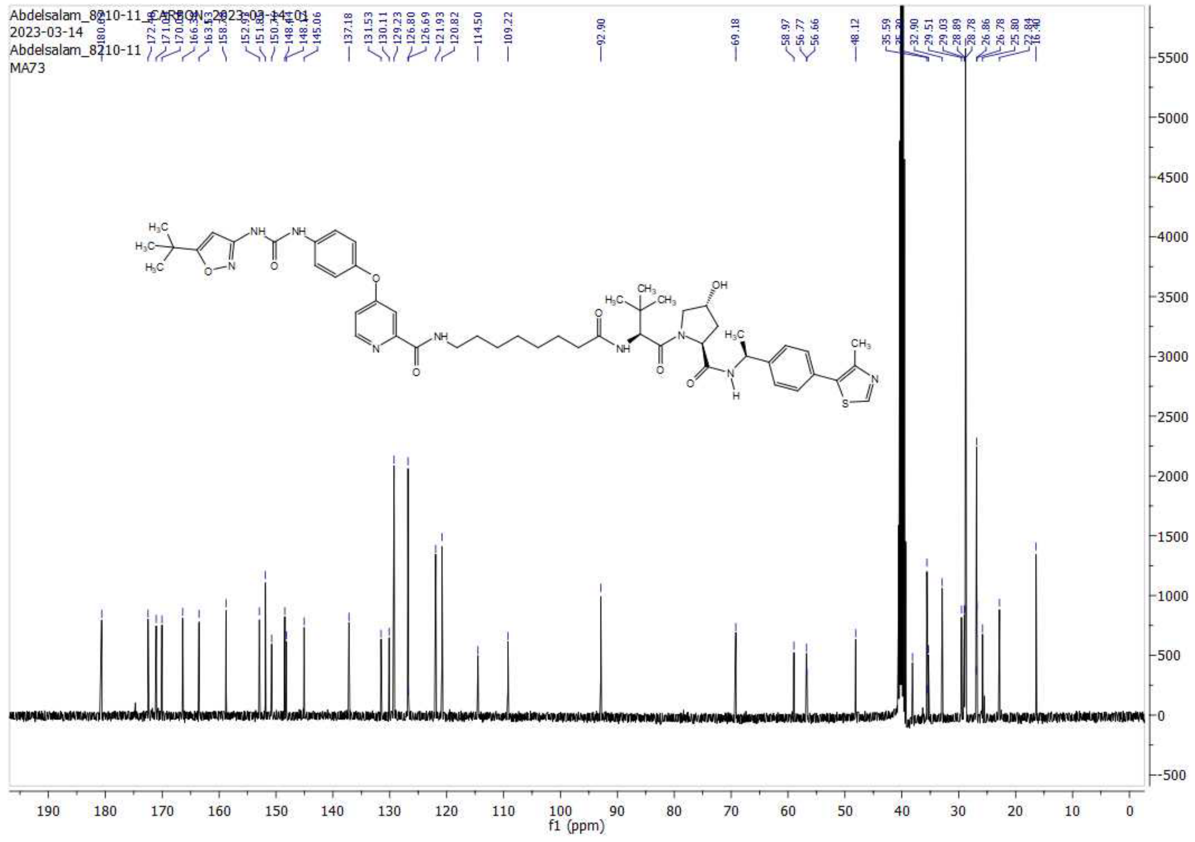

HRMS of 22e (MA73)

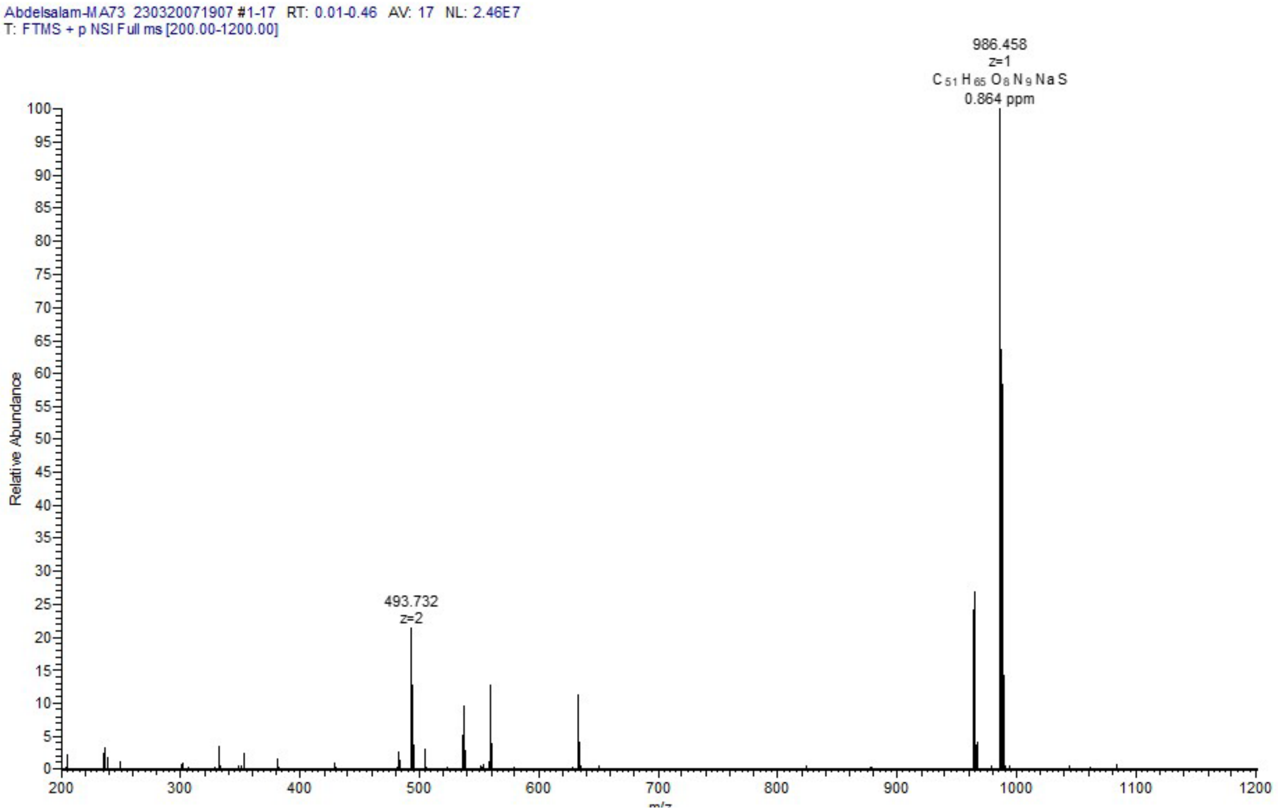

HPLC of 22e (MA73)

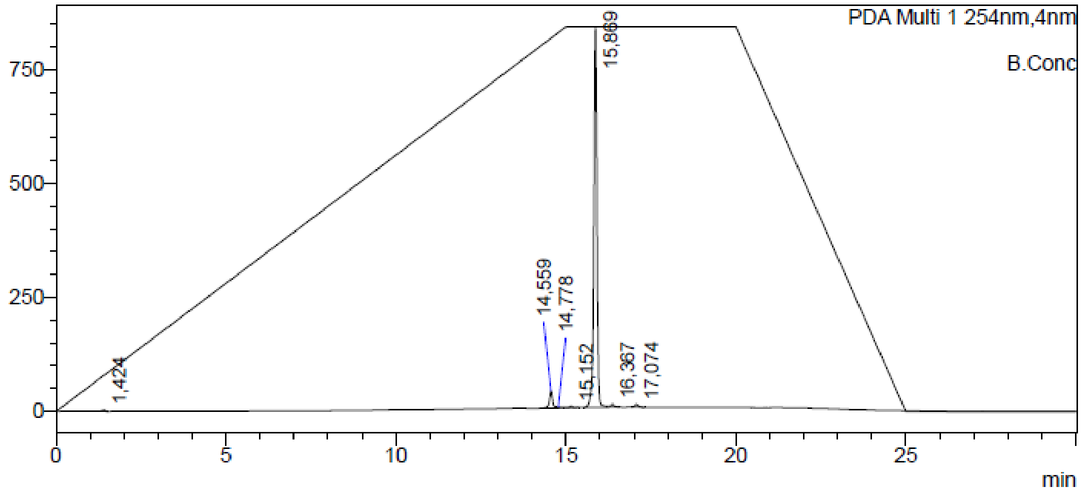

PDA Ch1 254nm

Peak#	Ret. Time	Area	Height	Area%
1	1,424	15449	2683	0,265
2	14,559	239373	37463	4,105
3	14,778	20453	2232	0,351
4	15,152	32088	3799	0,550
5	15,869	5437029	834205	93,238
6	16,367	50149	6372	0,860
7	17,074	36832	5719	0,632
Total		5831373	892473	100,000

^{1}HNMR of 30a (MA190)

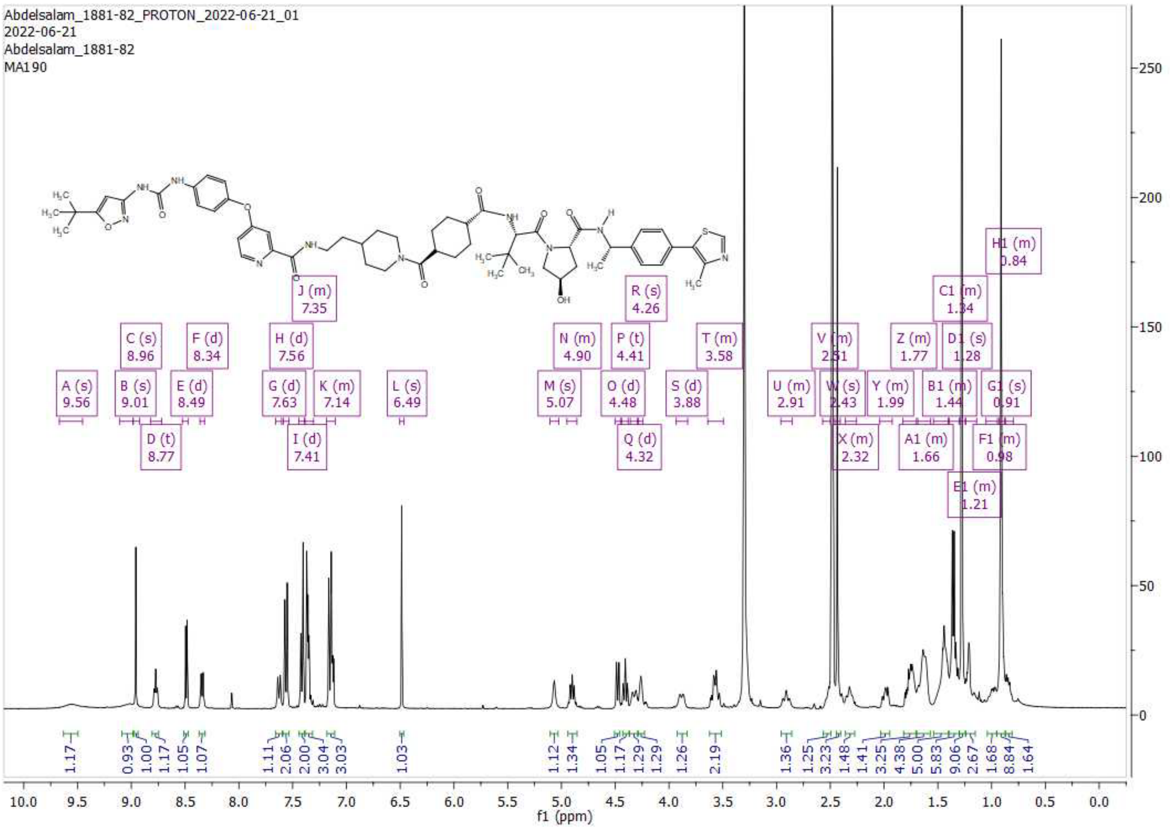

^{13}CNMR of 30a (MA190)

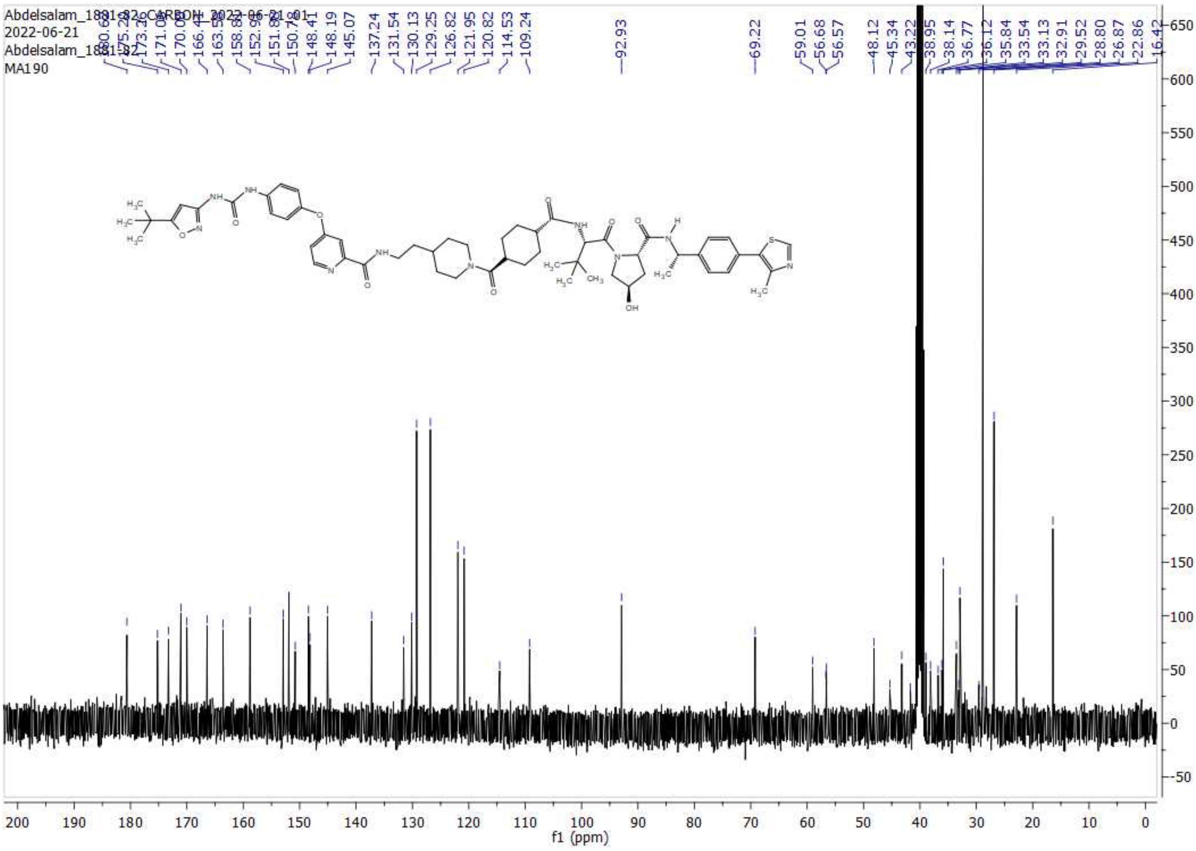

HRMS of 30a (MA190)

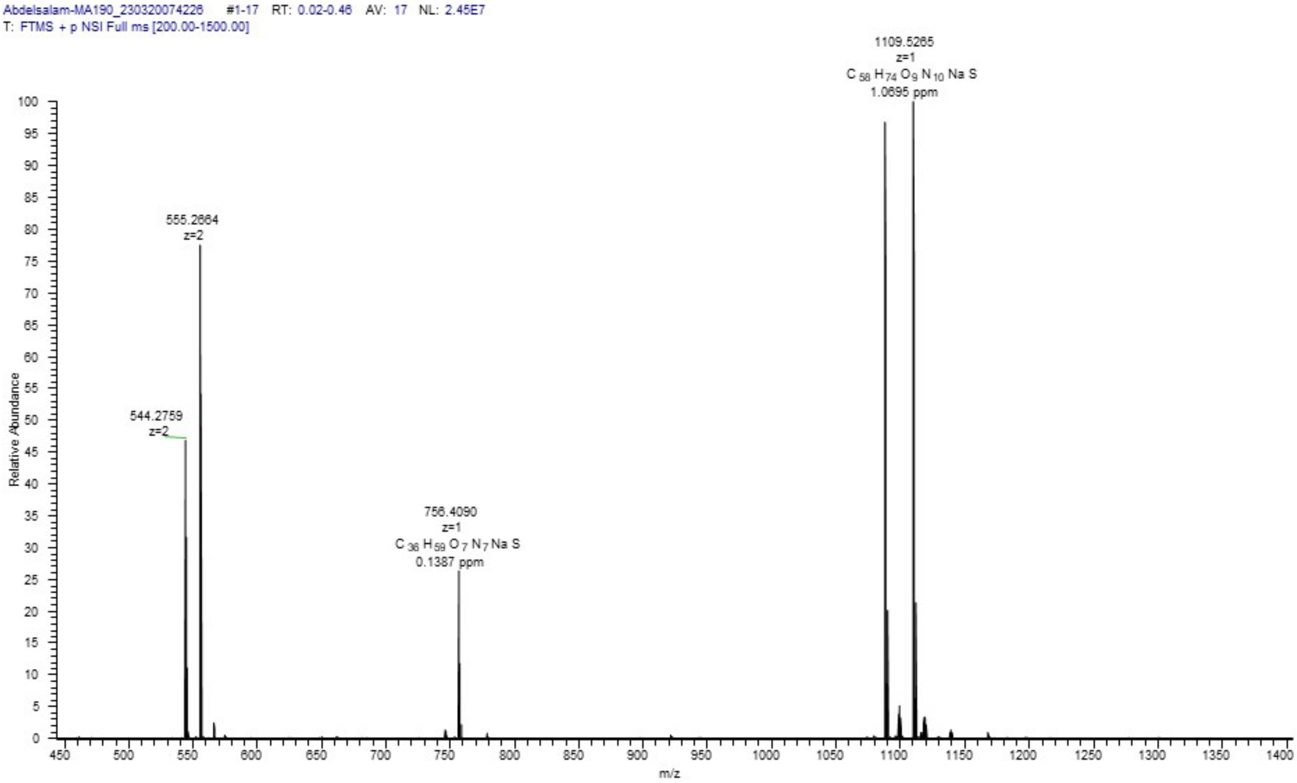

HPLC of 30a (MA190)

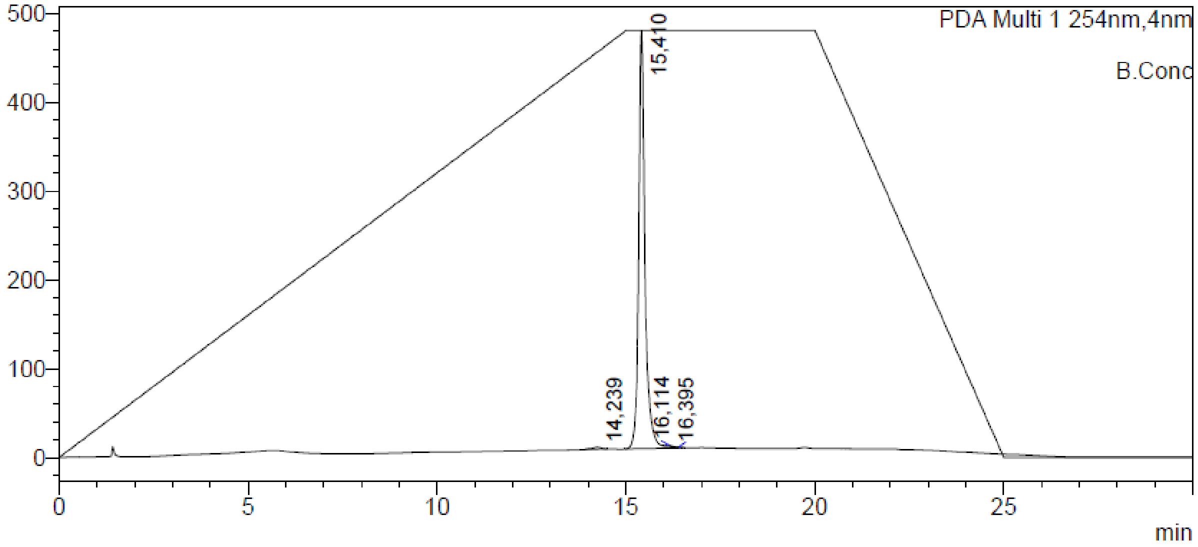

PDA Ch1 254nm

Peak#	Ret. Time	Area	Height	Area%
1	14,239	33884	1940	0,623
2	15,410	5389688	470879	99,076
3	16,114	13786	1474	0,253
4	16,395	2610	423	0,048
Total		5439968	474715	100,000

^{1}HNMR of 30b (MA191)

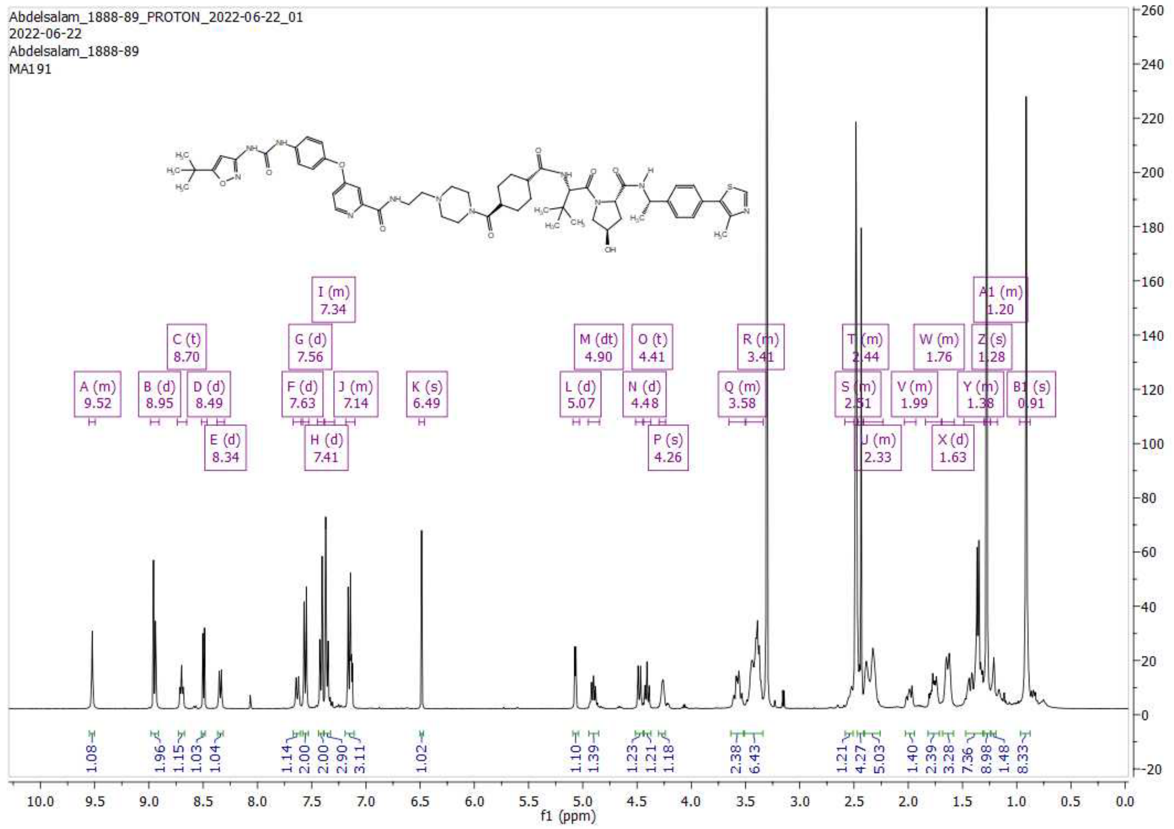

^{13}CNMR of 30b (MA191)

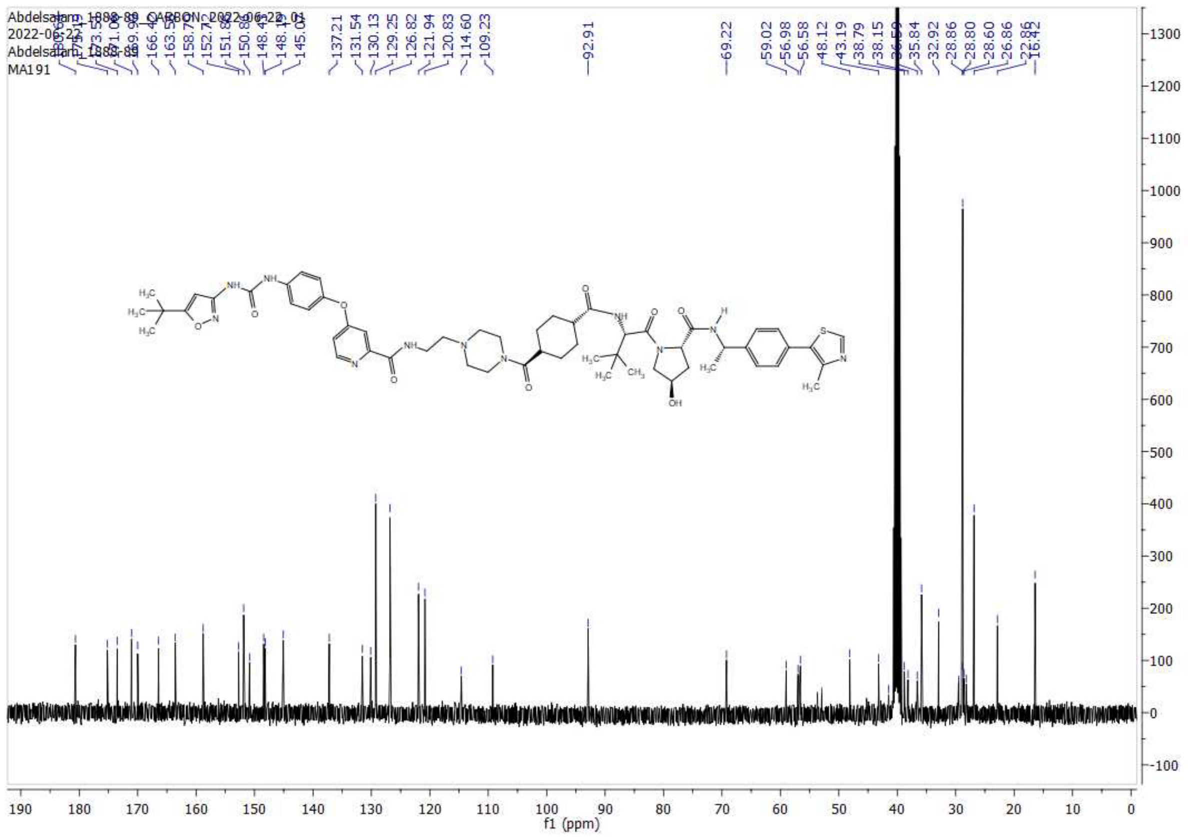

HRMS of 30b (MA191)

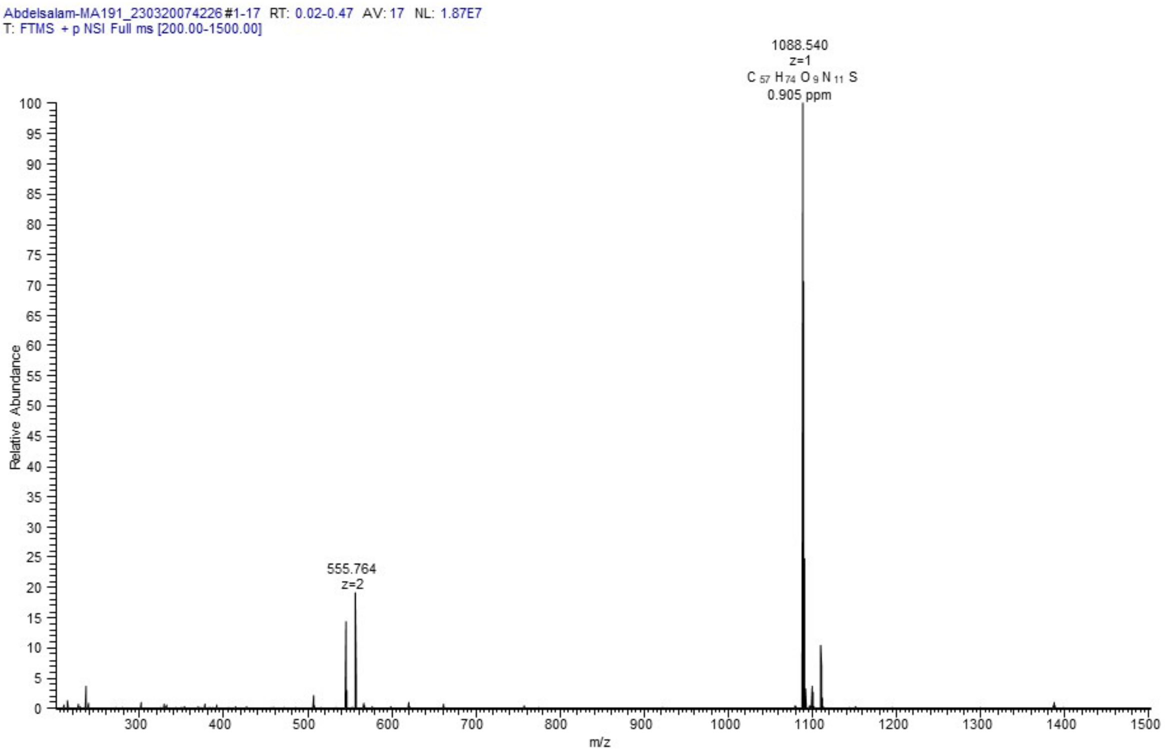

HPLC of 30b (MA191)

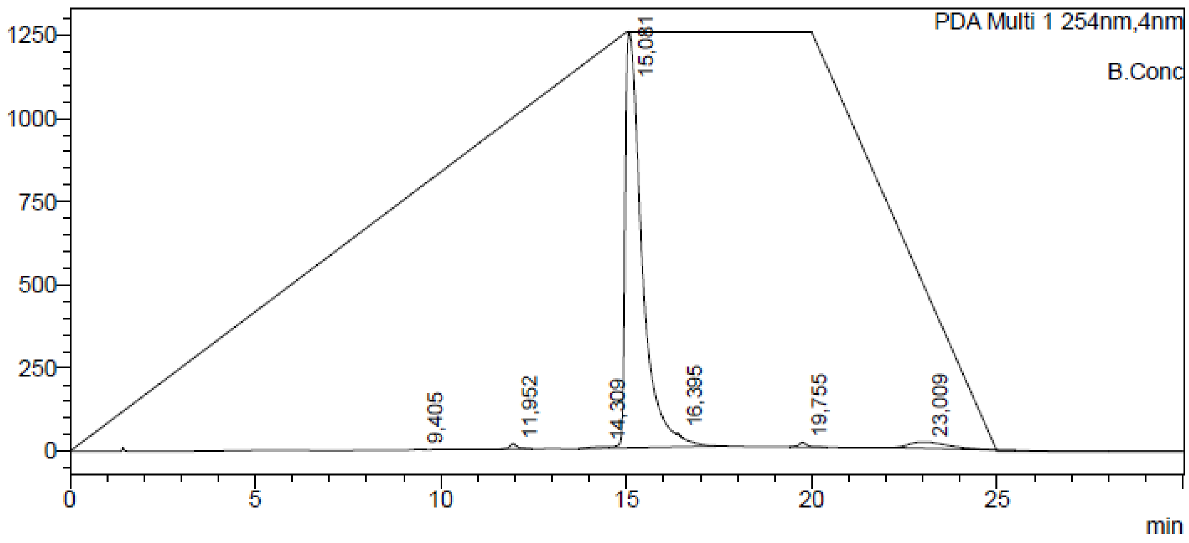

PDA Ch1 254nm

Peak#	Ret. Time	Area	Height	Area%
1	9,405	7842	898	0,019
2	11,952	214373	15625	0,516
3	14,309	145314	5031	0,350
4	15,081	39578976	1247850	95,200
5	16,395	17365	3749	0,042
6	19,755	214085	13873	0,515
7	23,009	1396574	18916	3,359
Total		41574529	1305941	100,000

^{1}HNMR of 31a (MA224)

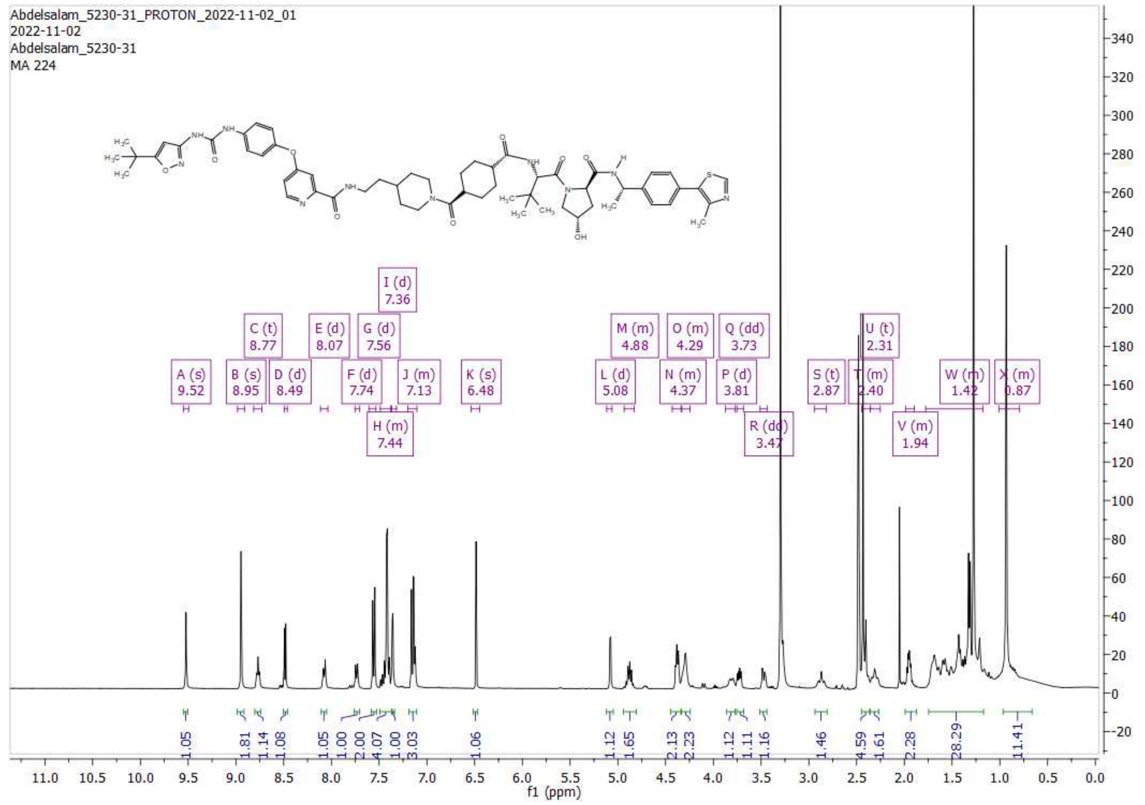

13 CNMR of 31a (MA224)

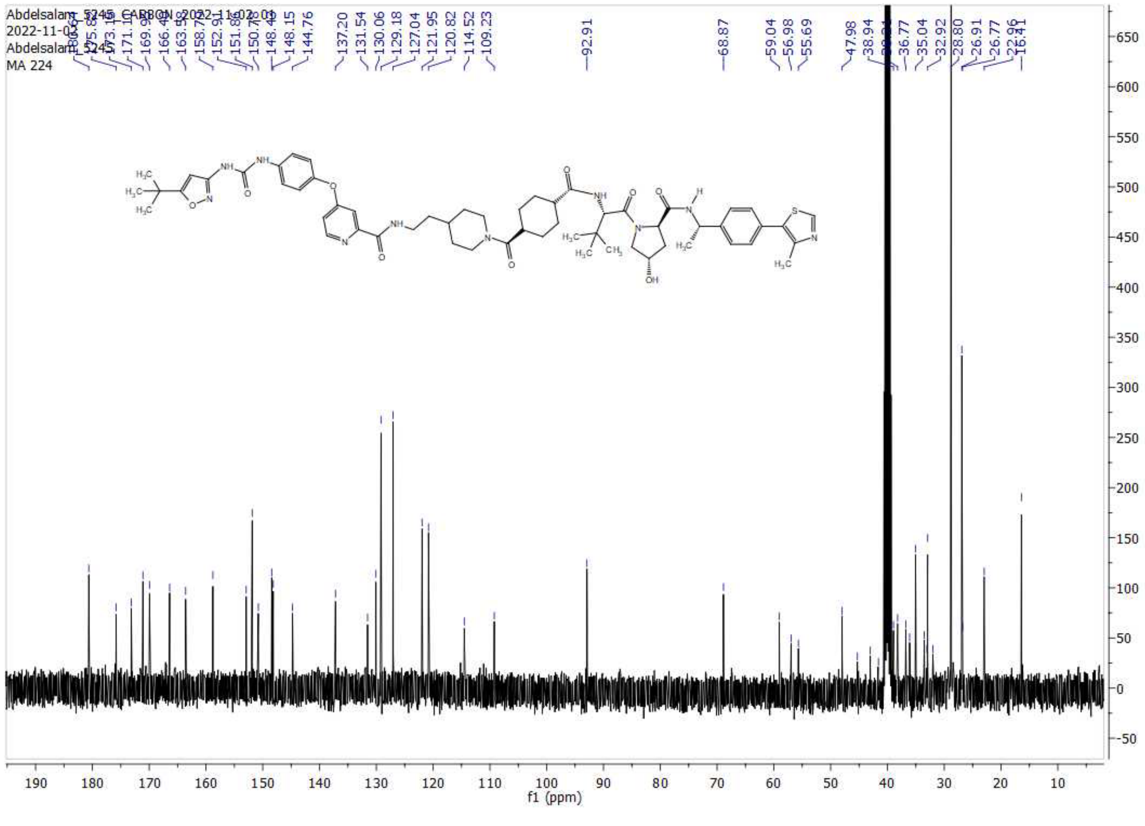

HRMS of 31a (MA224)

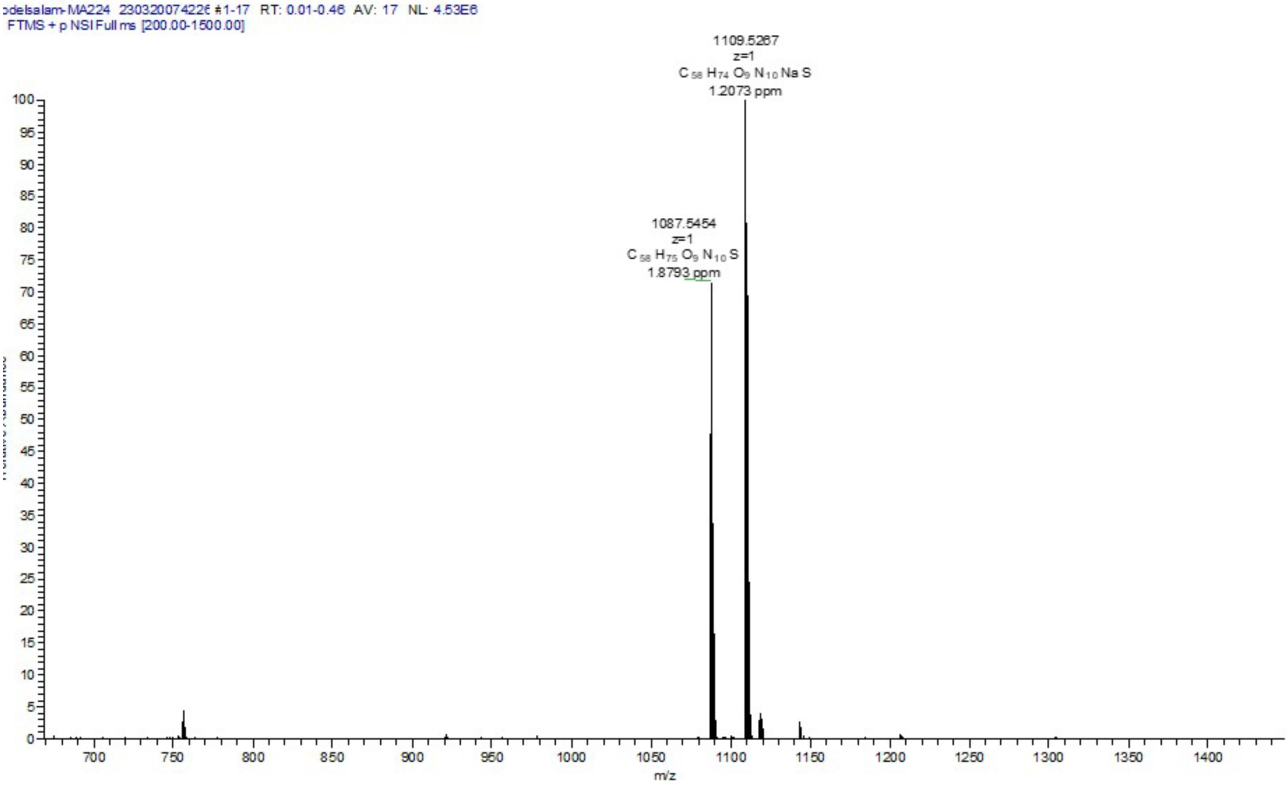

HPLC of 31a (MA224)

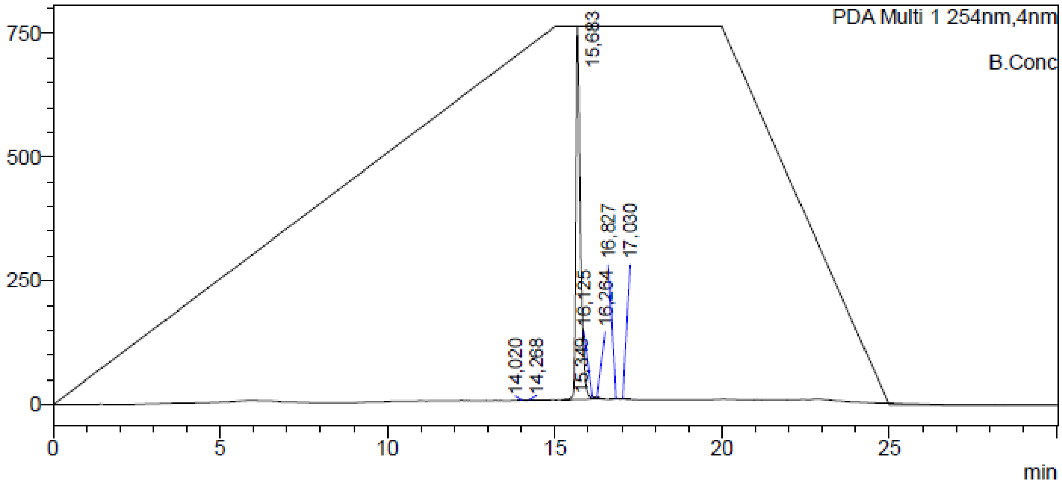

PDA Ch1 254nm

Peak#	Ret. Time	Area	Height	Area%
1	14,020	8086	1291	0,112
2	14,268	12851	2388	0,178
3	15,349	9075	1564	0,126
4	15,683	7065079	752968	98,018
5	16,125	30107	4830	0,418
6	16,264	41209	4676	0,572
7	16,827	24160	2851	0,335
8	17,030	17341	2906	0,241
Total		7207908	773474	100,000

^{1}HNMR of 31b (MA225)

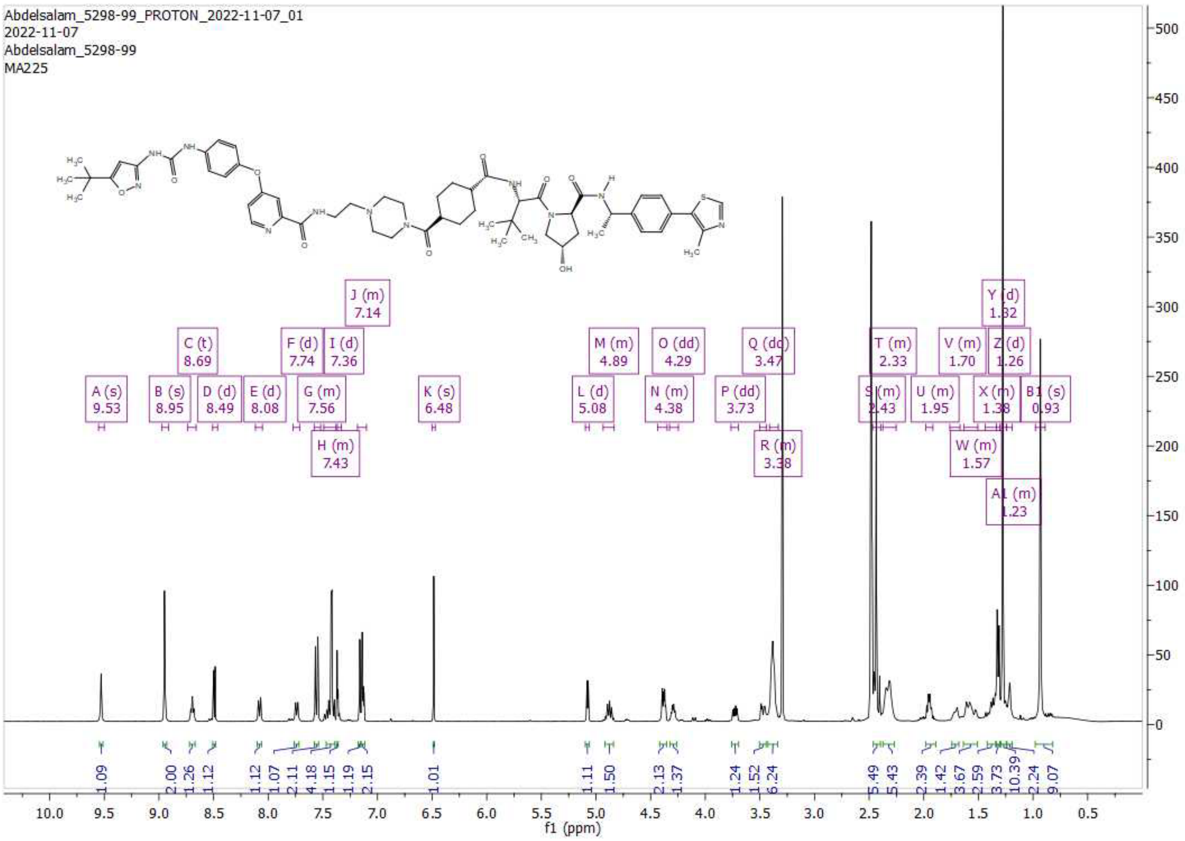

^{13}CNMR of 31b (MA225)

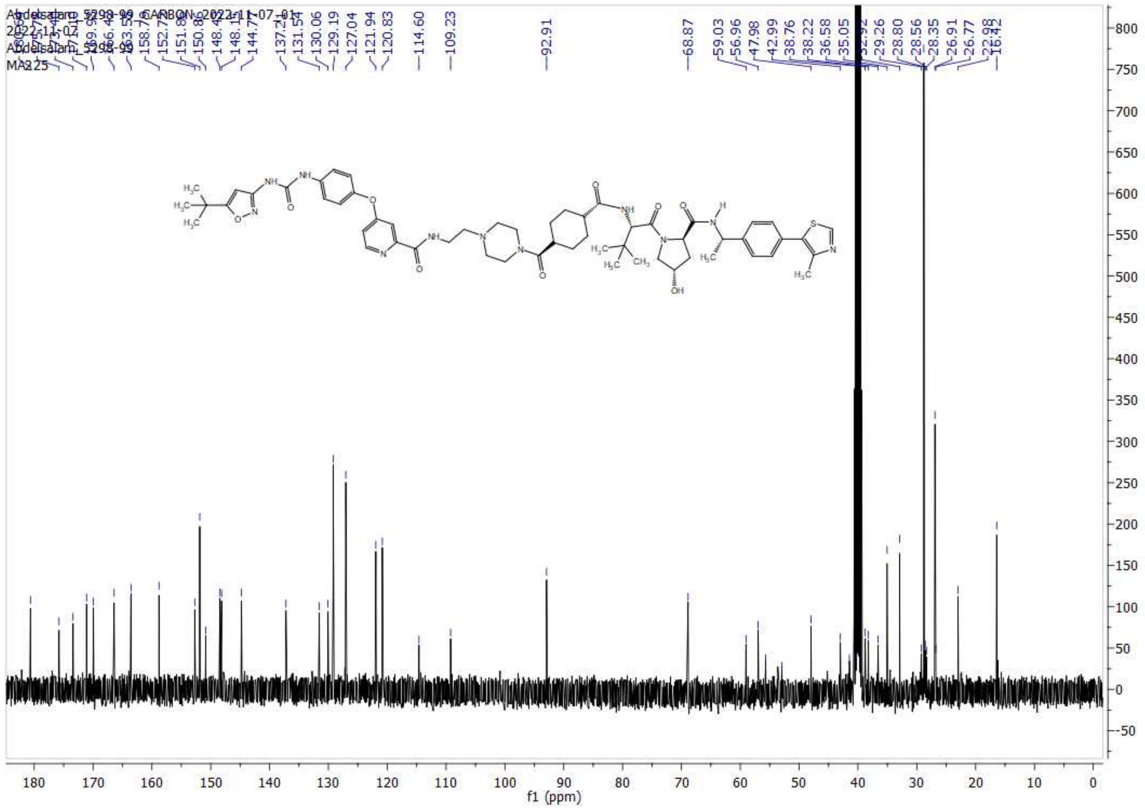

HRMS of 31b (MA225)

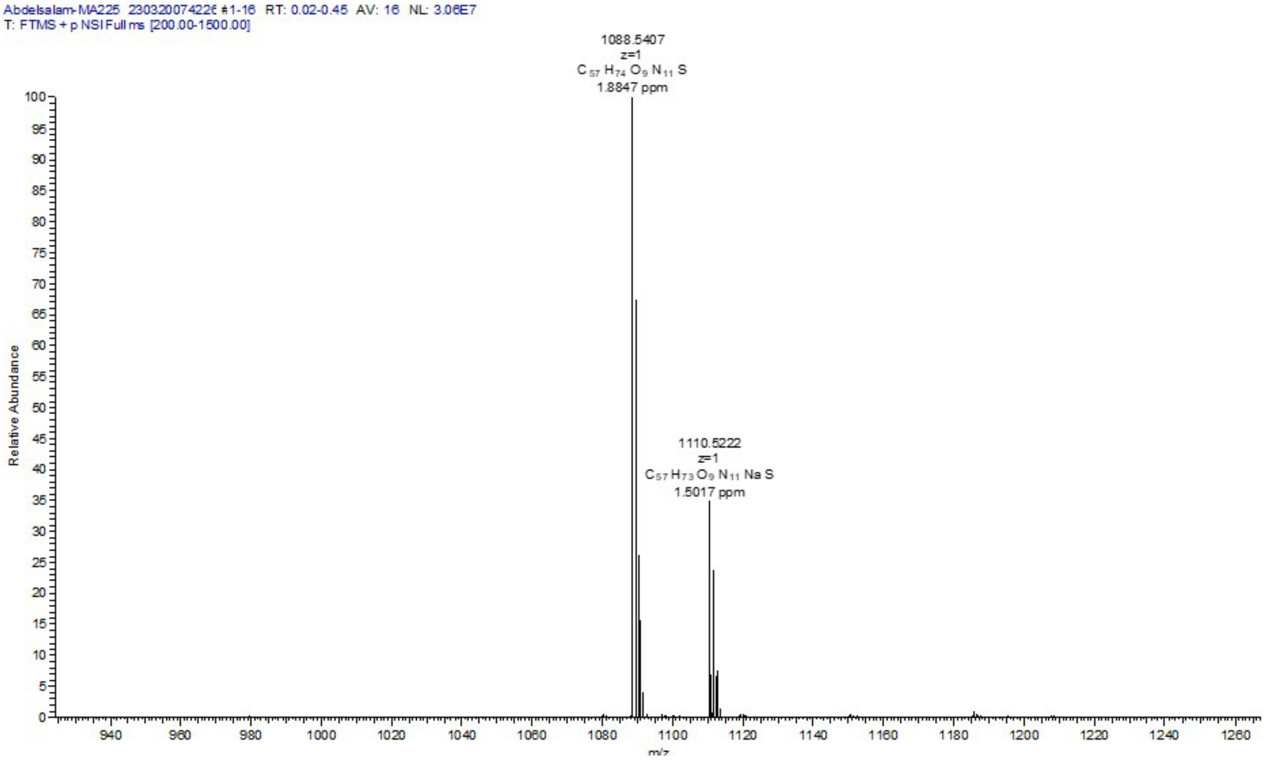

HPLC of 31b (MA225)

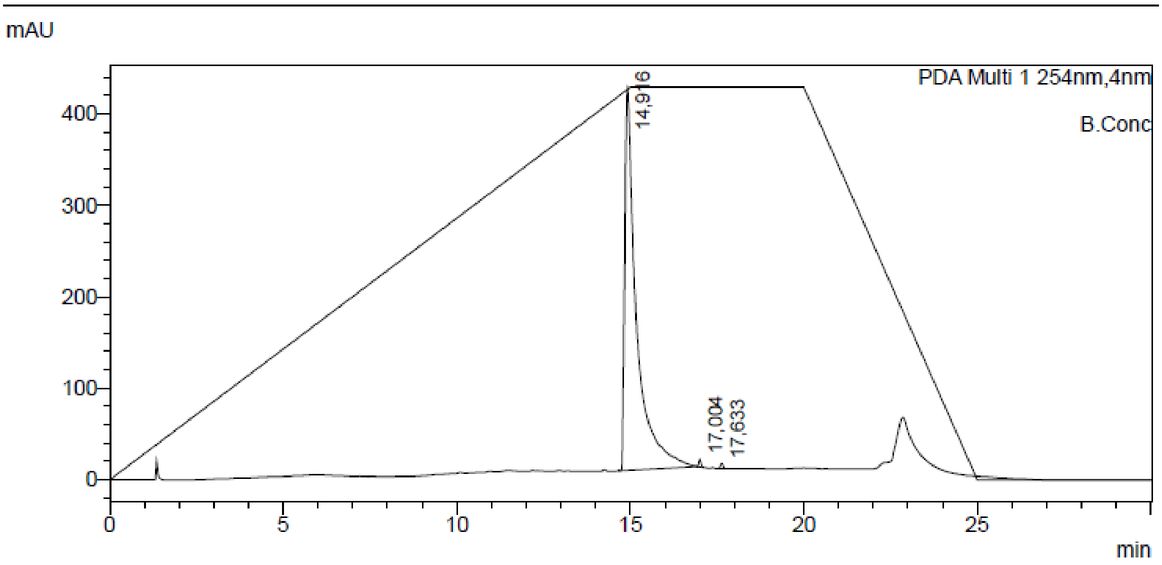

PDA Ch1 254nm

Peak#	Ret. Time	Area	Height	Area%
1	14,916	9824182	419119	99,316
2	17,004	35536	8036	0,359
3	17,633	32085	5669	0,324
Total		9891803	432825	100,000

Table S3. Microsomal stability assay of MA49, MA190, MA191 and control diclofenac

	0 min - compound remaining %	10 min - compound remaining %	20 min - compound remaining %	30 min - compound remaining %	60 min - compound remaining %	120 min - compound remaining %	RT
**Diclofenac_A**	100	93	87	80	73	63	14.4
**Diclofenac_B**	100	96	86	78	67	59	14.4
**MA49_A**	100	66	53	37	29	15	15.4
**MA49_B**	100	71	57	38	35	10	15.4
**MA190_A**	100	92	90	95	96	91	15.8
**MA190_B**	100	91	87	93	96	93	15.8
**MA191_A**	100	99	96	94	93	91	15.4
**MA191_B**	100	97	98	96	89	88	15.4

HPLC chromatograms

Run A

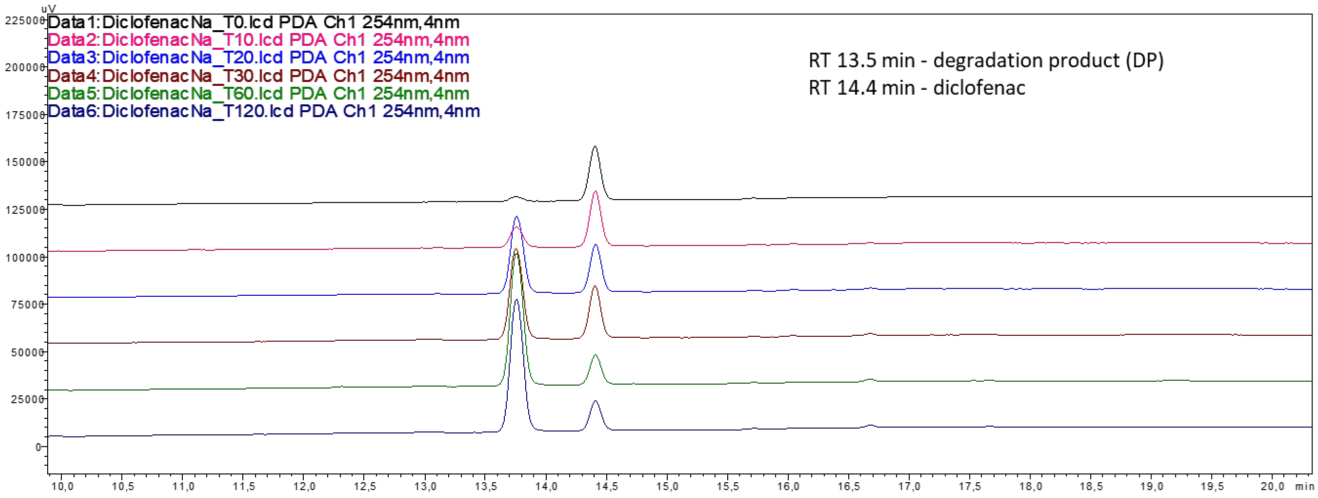

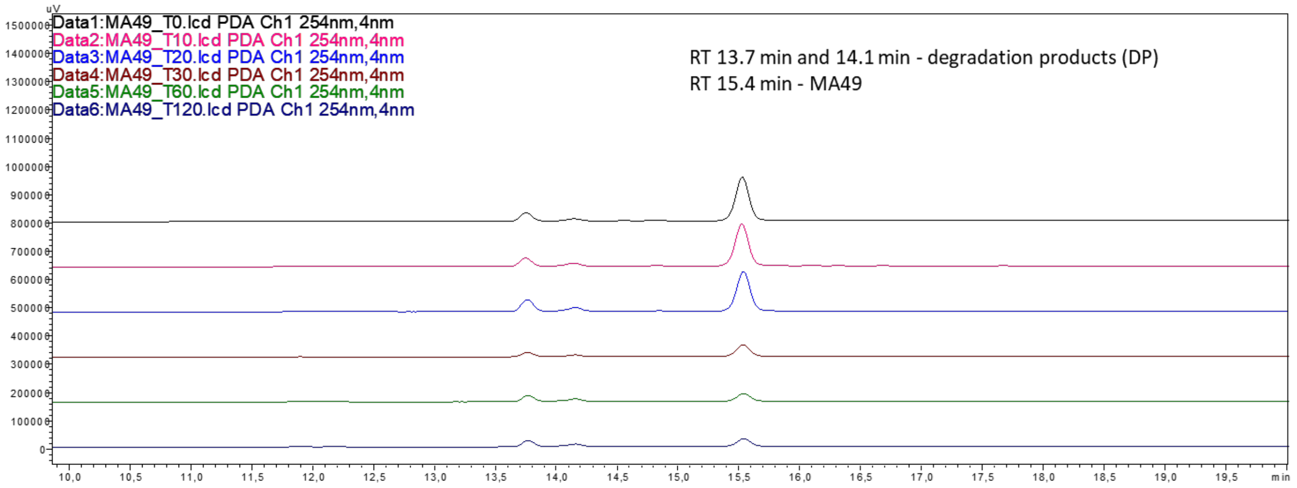

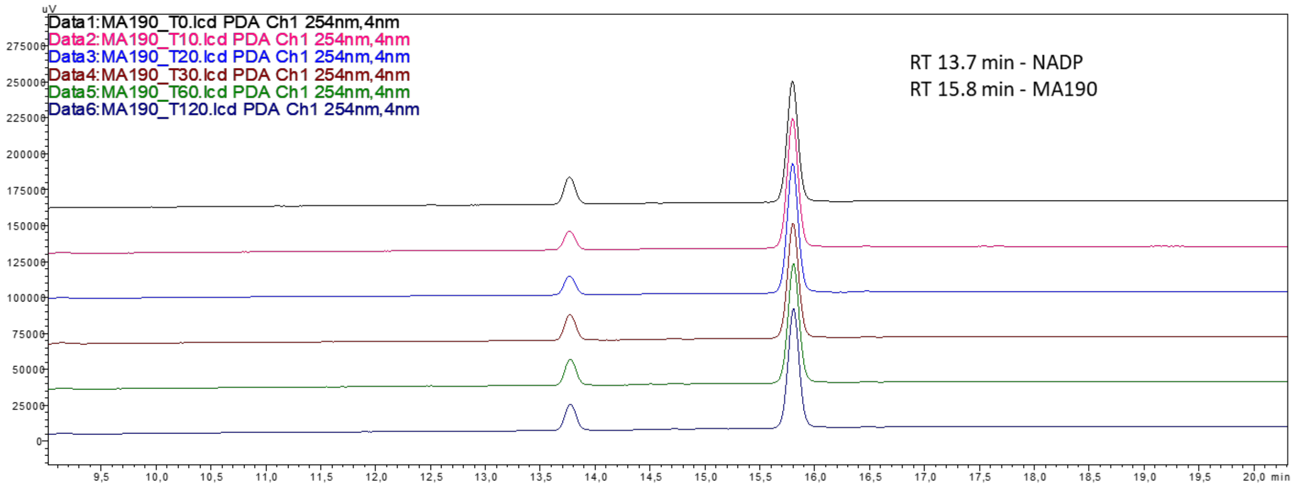

Data1:MA190_T0.lcd PDA Ch1 254nm,4nm
Data2:MA190_T10.lcd PDA Ch1 254nm,4nm
Data3:MA190_T20.lcd PDA Ch1 254nm,4nm
Data4:MA190_T30.lcd PDA Ch1 254nm,4nm
Data5:MA190_T60.lcd PDA Ch1 254nm,4nm
Data6:MA190_T120.lcd PDA Ch1 254nm,4nm
RT 13.7 min - NADP
RT 15.8 min - MA190

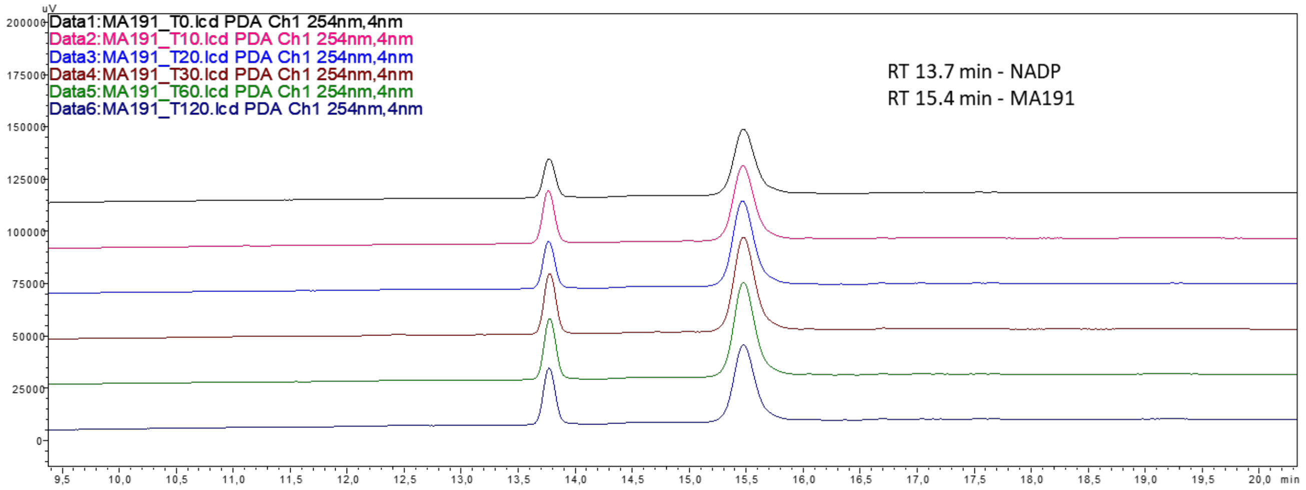

Data1:MA191_T0.lcd PDA Ch1 254nm,4nm
Data2:MA191_T10.lcd PDA Ch1 254nm,4nm
Data3:MA191_T20.lcd PDA Ch1 254nm,4nm
Data4:MA191_T30.lcd PDA Ch1 254nm,4nm
Data5:MA191_T60.lcd PDA Ch1 254nm,4nm
Data6:MA191_T120.lcd PDA Ch1 254nm,4nm
RT 13.7 min - NADP
RT 15.4 min - MA191

Run B

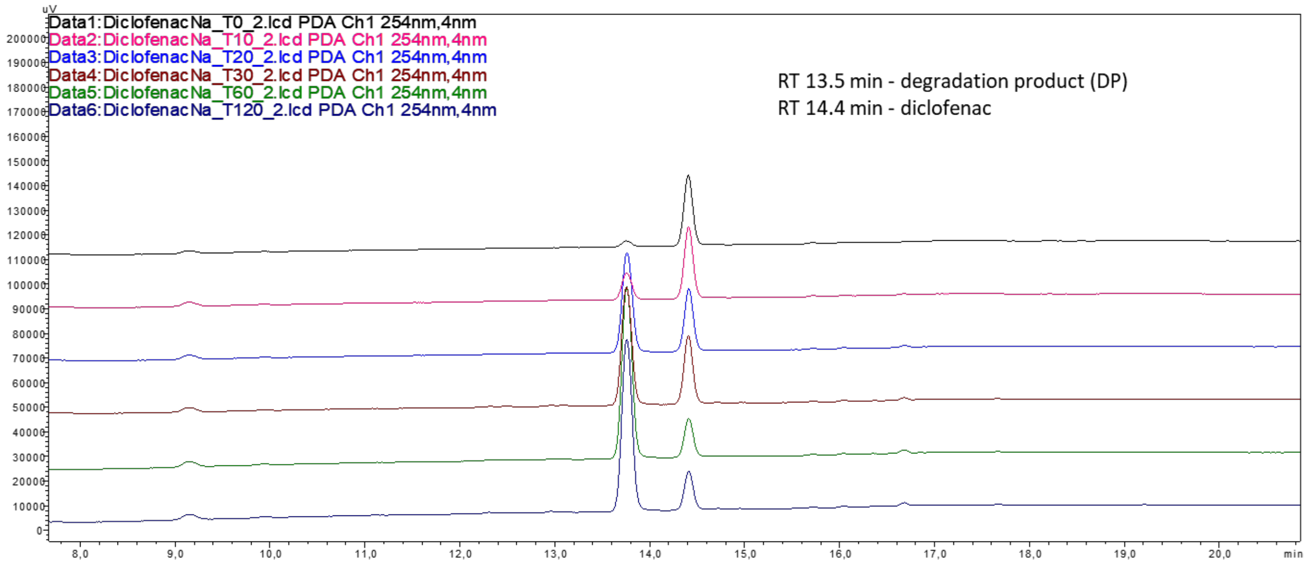

Data1:DiclofenacNa_T0_2.lcd PDA Ch1 254nm,4nm
Data2:DiclofenacNa_T10_2.lcd PDA Ch1 254nm,4nm
Data3:DiclofenacNa_T20_2.lcd PDA Ch1 254nm,4nm
Data4:DiclofenacNa_T30_2.lcd PDA Ch1 254nm,4nm
Data5:DiclofenacNa_T60_2.lcd PDA Ch1 254nm,4nm
Data6:DiclofenacNa_T120_2.lcd PDA Ch1 254nm,4nm
RT 13.5 min - degradation product (DP)
RT 14.4 min - diclofenac

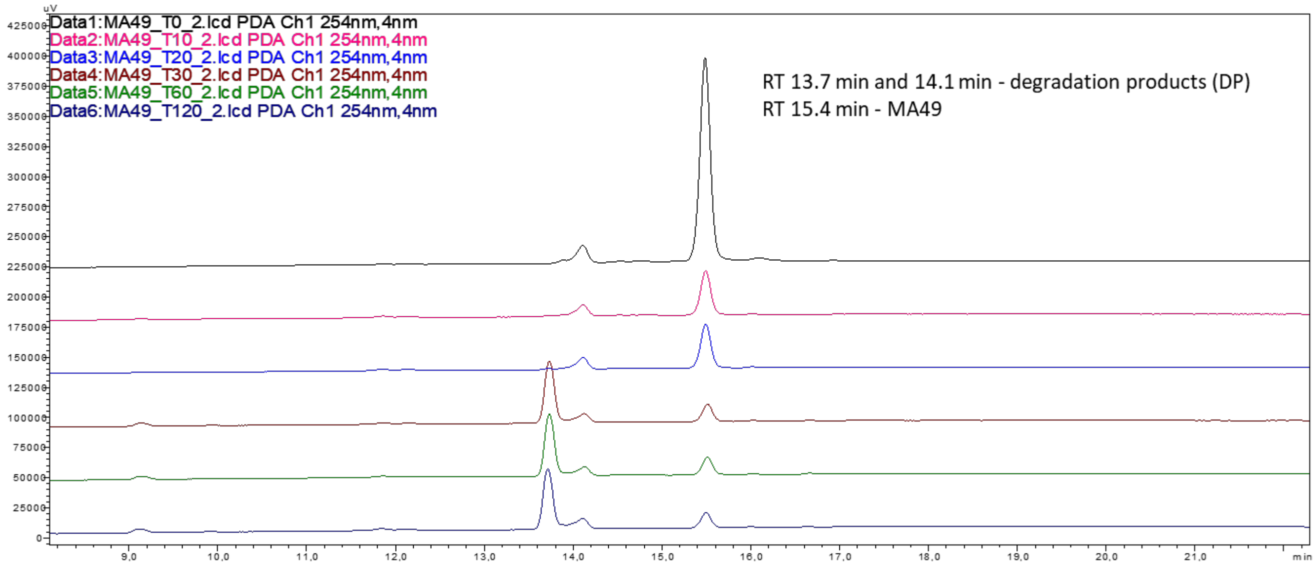

Data1:MA49_T0_2.lcd PDA Ch1 254nm,4nm
Data2:MA49_T10_2.lcd PDA Ch1 254nm,4nm
Data3:MA49_T20_2.lcd PDA Ch1 254nm,4nm
Data4:MA49_T30_2.lcd PDA Ch1 254nm,4nm
Data5:MA49_T60_2.lcd PDA Ch1 254nm,4nm
Data6:MA49_T120_2.lcd PDA Ch1 254nm,4nm
RT 13.7 min and 14.1 min - degradation products (DP)
RT 15.4 min - MA49

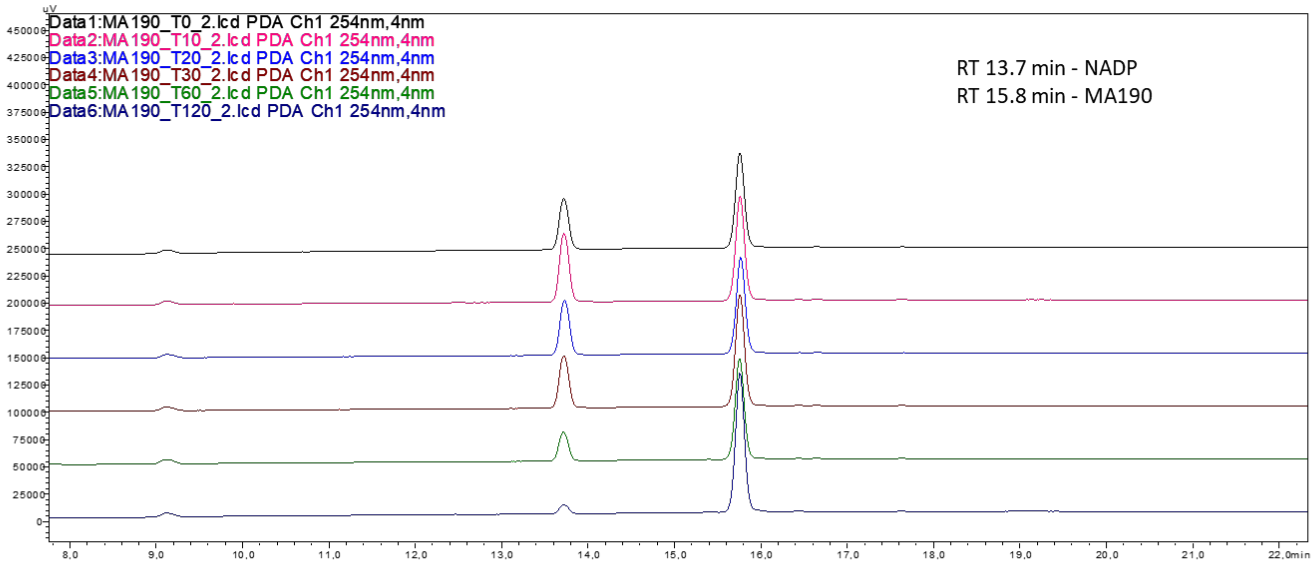

Data1:MA190_T0_2.lcd PDA Ch1 254nm,4nm
Data2:MA190_T10_2.lcd PDA Ch1 254nm,4nm
Data3:MA190_T20_2.lcd PDA Ch1 254nm,4nm
Data4:MA190_T30_2.lcd PDA Ch1 254nm,4nm
Data5:MA190_T60_2.lcd PDA Ch1 254nm,4nm
Data6:MA190_T120_2.lcd PDA Ch1 254nm,4nm
RT 13.7 min - NADP
RT 15.8 min - MA190

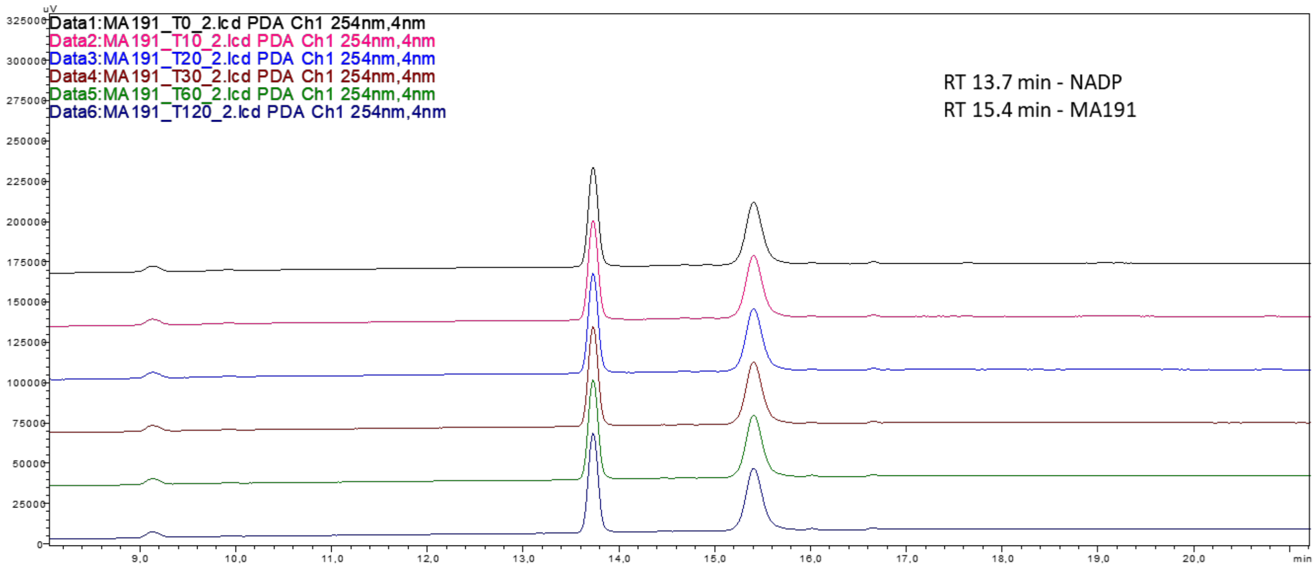

uV
Data1:MA191_T0_2.lcd PDA Ch1 254nm,4nm
Data2:MA191_T10_2.lcd PDA Ch1 254nm,4nm
Data3:MA191_T20_2.lcd PDA Ch1 254nm,4nm
Data4:MA191_T30_2.lcd PDA Ch1 254nm,4nm
Data5:MA191_T60_2.lcd PDA Ch1 254nm,4nm
Data6:MA191_T120_2.lcd PDA Ch1 254nm,4nm
RT 13.7 min - NADP
RT 15.4 min - MA191
min
